# New 1,2,3-triazole linked ciprofloxacin-chalcones induce DNA damage by inhibiting human topoisomerase I& II and tubulin polymerization

**DOI:** 10.1080/14756366.2022.2072308

**Published:** 2022-05-11

**Authors:** Hamada H. H. Mohammed, Amer Ali Abd El-Hafeez, Kareem Ebeid, Aml I. Mekkawy, Mohammed A. S. Abourehab, Emad I. Wafa, Suhaila O. Alhaj-Suliman, Aliasger K. Salem, Pradipta Ghosh, Gamal El-Din A. Abuo-Rahma, Alaa M. Hayallah, Samar H. Abbas

**Affiliations:** a Department of Medicinal Chemistry, Faculty of Pharmacy, Minia University, Minia, Egypt; b Department of Pharmaceutical Chemistry, Faculty of Pharmacy, Sohag University, Sohag, Egypt; c Department of Cellular and Molecular Medicine, University of California San Diego, La Jolla, CA, USA; d Cancer Biology Department, Pharmacology and Experimental Oncology Unit, National Cancer Institute, Cairo University, Cairo, Egypt; e Department of Pharmaceutics, Faculty of Pharmacy, Minia University, Minia, Egypt; f Department of Pharmaceutical Sciences and Experimental Therapeutics, College of Pharmacy, University of Iowa, Iowa City, IA, USA; g Department of Pharmaceutics, Faculty of Pharmacy and Pharmaceutical Manufacturing, Deraya University, New Minia City, Minia, Egypt; h Department of Pharmaceutics and Clinical Pharmacy, Faculty of Pharmacy, Sohag University, Sohag, Egypt; i Department of Pharmaceutics, Faculty of Pharmacy, Umm Al Qura University, Makkah, Saudi Arabia; j Holden Comprehensive Cancer Center, University of Iowa, Iowa City, IA, USA; k Department of Medicine, University of California San Diego, La Jolla, CA, USA; l Rebecca and John Moore Comprehensive Cancer Center, University of California San Diego, La Jolla, CA, USA; m Veterans Affairs Medical Center, La Jolla, CA, USA; n Department of Pharmaceutical Chemistry, Faculty of Pharmacy, Deraya University, Minia, Egypt; o Pharmaceutical Organic Chemistry Department, Faculty of Pharmacy, Assiut University, Assiut, Egypt; p Pharmaceutical Chemistry Department, Faculty of Pharmacy, Sphinx University, New Assiut, Egypt

**Keywords:** Click synthesis, ciprofloxacin, chalcone, cytotoxicity, HCT116 cells, topoisomerase I and II inhibitors, tubulin polymerisation inhibition, DNA damage

## Abstract

A series of novel 1,2,3-triazole-linked ciprofloxacin-chalcones **4a-j** were synthesised as potential anticancer agents. Hybrids **4a-j** exhibited remarkable anti-proliferative activity against colon cancer cells. Compounds **4a-j** displayed IC_50_s ranged from 2.53-8.67 µM, 8.67–62.47 µM, and 4.19–24.37 µM for HCT116, HT29, and Caco-2 cells; respectively, whereas the doxorubicin, showed IC_50_ values of 1.22, 0.88, and 4.15 µM. Compounds **4a, 4b, 4e, 4i,** and **4j** were the most potent against HCT116 with IC_50_ values of 3.57, 4.81, 4.32, 4.87, and 2.53 µM, respectively, compared to doxorubicin (IC_50_ = 1.22 µM). Also, hybrids **4a, 4b, 4e, 4i,** and **4j** exhibited remarkable inhibitory activities against topoisomerase I, II, and tubulin polymerisation. They increased the protein expression level of γH2AX, indicating DNA damage, and arrested HCT116 in G2/M phase, possibly through the ATR/CHK1/Cdc25C pathway. Thus, the novel ciprofloxacin hybrids could be exploited as potential leads for further investigation as novel anticancer medicines to fight colorectal carcinoma.

## Graphical Abstract

**Figure:**
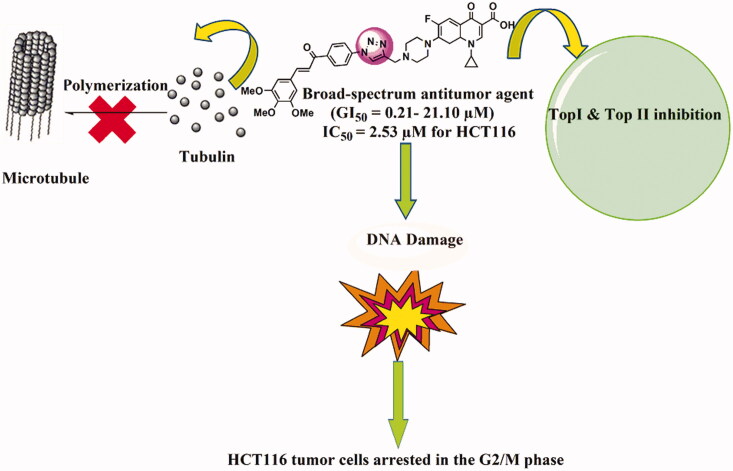

## 1. Introduction

**Figure 1.**
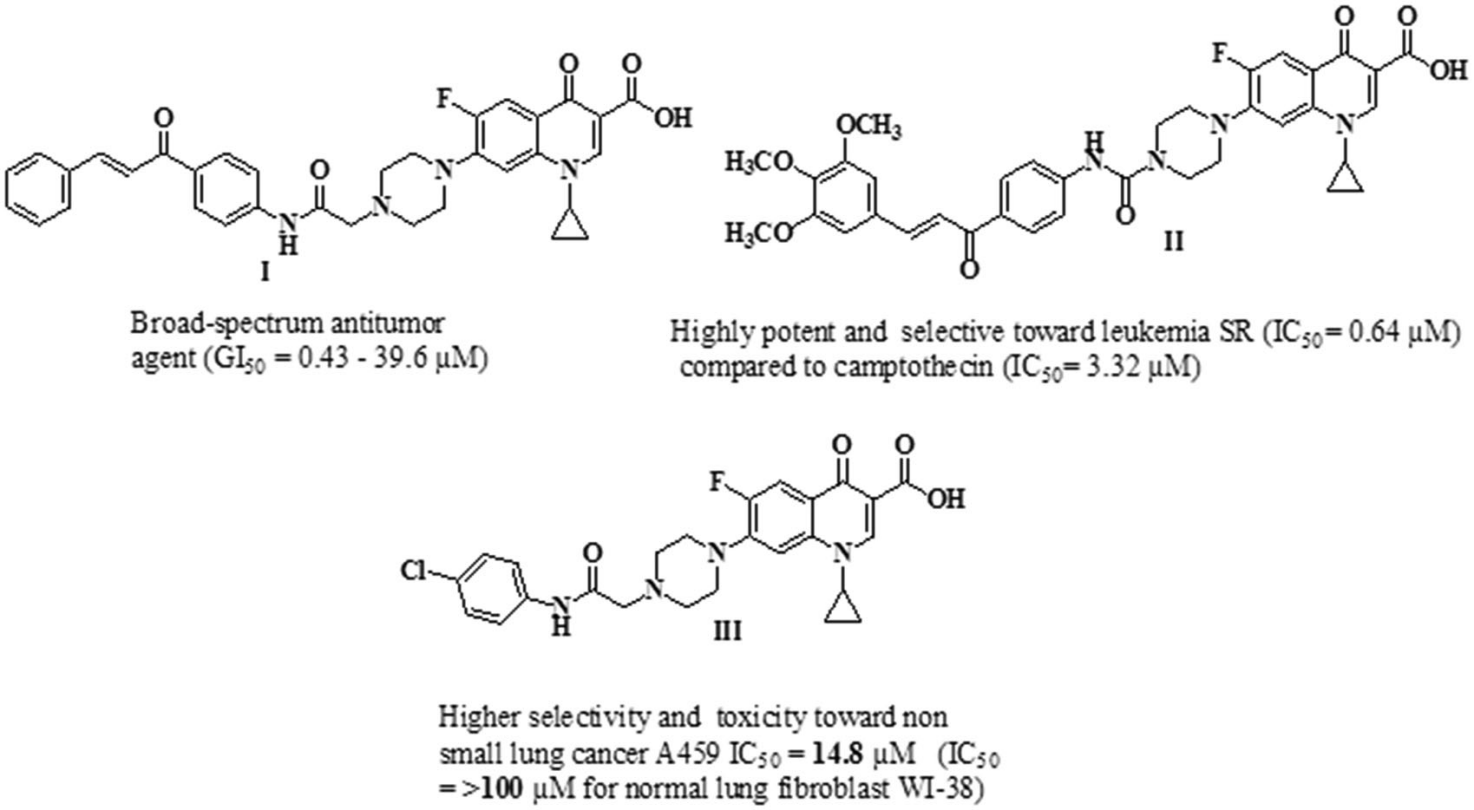
Chemical structures of several examples of some reported *N*-4 piperazinyl ciprofloxacin derivatives with anti-proliferative activities.

Cancer is a complicated pathological condition characterised by uncontrolled cell growth^1^. As the 2nd leading cause of mortality, cancer accounts for 21% of annual deaths globally^2–4^. By 2040, the number of new cases is estimated to reach 29.4 million per year, compared to 18.1 million in 2018^5^. Colorectal Cancer (CRC) was the second most deadly cancer and the third most common malignancy in 2020, which caused 1.9 million incidence cases and 0.9 million deaths globally. The Predictable number of new CRC cases worldwide in 2040 will be 3.2 million cases^6^. Surgery, chemotherapy, and radiotherapy are currently available cancer treatment options^7^. Although single target chemotherapy achieves some therapeutic benefits, its use is limited by poor efficacy, serious toxic effects and the development of resistance^8^^,^^9^. Two strategies for multi-target therapeutics are being used in preclinical or clinical practice to overcome such problems^10^. The first one is combination therapy; the clinical use of this approach is widely utilised for the treatment of various types of cancer. Still, some combinations may be limited due to undesirable drug-drug interactions^11^. The second strategy is based on using smart hybrids or multi-target directed ligands (MTDLs). Two or more pharmacophore fragments are linked together to form a single molecule that can simultaneously modulate multiple oncoproteins^12^^,^^13^. Thus, the use of multi-target directed ligands can improve efficacy, reduce serious adverse effects, and prevent the development of drug resistance^14^^,^^15^. Understanding various molecular pathways utilised in the development and formation of tumours can aid in the discovery of novel antitumor agents^16^. Ciprofloxacin (CP) is a broad-spectrum antibacterial agent that acts by poisoning bacterial DNA gyrase (topoisomerase II) and topoisomerase IV^17^^,^^18^, which are fundamental enzymes that substantially contribute to the process of DNA replication^19–21^. CP has recently gained considerable attention as an anti-proliferative agent^21^^,^^22^. It was reported that poisoning topoisomerase II, arresting cell cycle arrest, and inducing apoptosis are the mechanisms responsible for the anti-proliferative activity of CP^23–25^. Accordingly, shifting the biological profile of CP from antibacterial to anticancer candidate appears to be possible due to its profound apoptotic and anti-proliferative potential^26^. In this regard, the structural modifications required for anticancer activity of CP have been clearly discussed^1^^,^^27^. Bio-isosteric replacement of the carboxylic group at C-3 of CP with tetrazole heterocyclic ring markedly improves its anti-proliferative activity^1^^,^^28^. Also, one key modification was carried out by substituting its *N*-4 piperazine heterocycle moiety with an aryl or heteroaryl bulky group^1^^,^^27^. Introducing a substituent on the *N*-4-piperazinyl moiety of CP affects the physicochemical characteristics and/or significantly improves the anti-proliferative activity^29–31^. The nature of the *N*-4-piperazinyl substituent remarkably affects the capacity of CP derivatives to suppress the activity of DNA topoisomerases^32^. In addition, the potential selectivity of CP derivatives to human topoisomerases was found to be significantly improved via modification of the *N*-4-piperazine heterocycle^31^. Consequently, several CP derivatives have been prepared, characterised, and widely explored for their antitumor properties^33^^,^^34^. Recent studies have revealed that the presence of different chalcones on the *N*-4-piperazine moiety of CP gave rise to new CP hybrids with potent anti-proliferative activities targeting multiple oncoproteins (Figure 1)^16^^,^^21^. Furthermore, the introduction of the aryl sulfamoyl group into the *N*-4 piperazine of CP gave rise to new fluoroquinolone derivatives with anticancer activity^35^. Substitution of *N*-4 piperazine of CP with 4-chlorophenylcarbamoyl led to a new CP derivative with potent anti-proliferative activity against non-small cell lung cancer (Figure 1)^36^. On the other hand, natural and synthetic chalcones represent a vital and central component of various compounds with diverse biological properties^37^. Chalcone is a familiar scaffold in medicinal chemistry that is widely used for the development of a large number of molecules with potential anticancer activities^38^^,^^39^. The anticancer potential of chalcone-containing compounds was attributed to apoptosis induction, angiogenesis inhibition, DNA damage, kinases inhibition, and tubulin inhibition^40^^,^^41^. Chalcones have gained considerable attention from drug hunters due to their ease of synthesis, exploring novel scaffolds with potential biological activities^42^^,^^43^.

**Figure 2.**
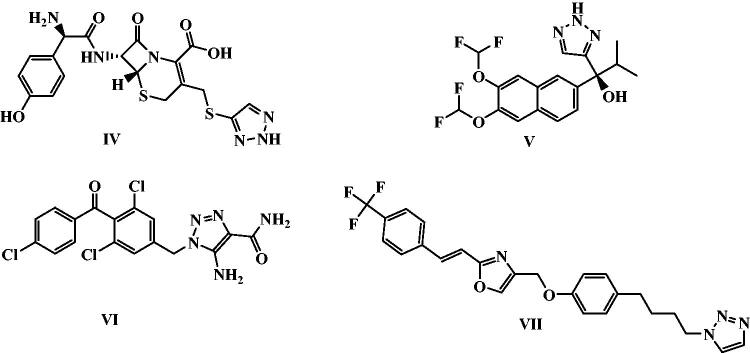
Potential anticancer drugs based on 1,2,3-triazole nucleus in active clinical trials.

Furthermore, 1,2,3-Triazoles are essential scaffolds in medicinal chemistry that are widely used in many drug-design protocols as bio-isosteres of ester, amide, and other heterocycles^44^^,^^45^. Compounds containing 1,2,3-triazole heterocycle can form different non-covalent interactions such as hydrogen bonds, dipole-dipole bonds, hydrophobic interactions, and van der Waals forces with various biological targets, thus they possess various biological effects (e.g., anticancer^46–49^, antibacterial^50^^,^^51^, antifungal^52^^,^^53^, antiviral^54^^,^^55^, antimalarial^56^^,^^57^, and antitubercular^58^^,^^59^ activities). Additionally, 1,2,3-Triazole is a favourable basic unit in the finding of new anticancer agents, and some of its derivatives have now been in clinics or under clinical trials for combating against cancers^45^. 1,2,3‐Triazoles exhibited their anticancer effects via different modes of action. They exerted their anti-proliferative effects through Tubulin polymerisation inhibition or inhibition of some kinases such as epidermal growth factor receptor, c-Met Kinase, and vascular endothelial growth factor. They also caused inhibition of vital enzymes such as aromatase, tryptophan 2,3‐dioxygenase, carbonic anhydrases, and thymidylate synthase^60^. Also, many drugs have 1,2,3-triazole moiety, such as cefatrizine **IV**, seviteronel **V**, carboxyamidotriazole **VI**, and mubritinib **VII** entered active clinical trials in 2021 (Figure 2)^61^.

**Figure 3.**
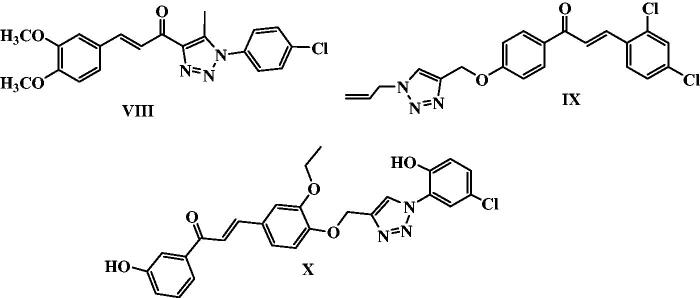
Some recently reported anti-proliferative 1,2,3-triazole/chalcone hybrids.

Moreover, several 1,2,3-triazole chalcone hybrids were reported as anticancer agents aiming for synergistic activity in the last decade. For example, 1,2,3-triazole-chalcone hybrid **VIII** exhibited nanomolar IC_50_ values against six cancer cell lines, HCT-116 colon, RPMI-8226 leukaemia, MCF7 breast, SR leukaemia, M14 melanoma, and K-562 leukaemia cancer cell lines, and sensitive cell lines. It caused induction of apoptosis in a dose-dependent manner through activation of caspases 3, 7 & 9 and Bax/Bcl-2 ratio elevation^62^. Additionally, Chalcone/1,2,3-triazole hybrid **IX** was reported as an orally tubulin polymerisation inhibitor. Compound **IX** inhibited proliferation and migration in HepG2 cells growth in a dose-dependent manner^63^. Furthermore, 1,2,3-triazole tethered chalcone hybrid **X** showed potent cytotoxic activity on MCF-7 cancer cell lines with IC_50_ of 1.27 µM and 0.02 µM at 24 and 48 h; respectively^64^ (Figure 3).

**Figure 4.**
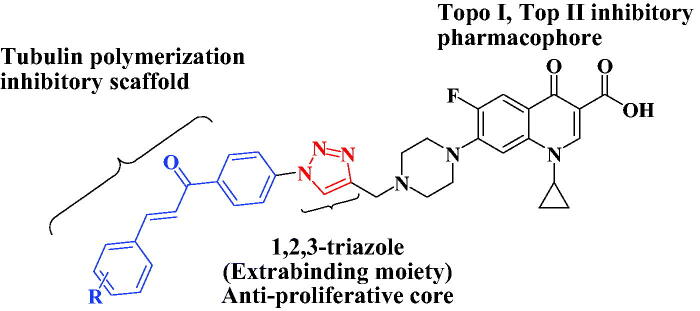
Pharmacophore of target compounds **4a-j**.

Inspired by the above-mentioned aspects, the purpose of our study was to develop a new series of 1,2,3-triazole linked CP-chalcone hybrids **4a-j** as possible multi-target-directed ligands with potential anti-proliferative activities. Their *in-vitro* cytotoxic activity was evaluated using National Cancer Institute (NCI) aganist59 cancer cell lines. Additionally, the IC_50_s of the new CP derivatives **4a-j** against three different colon cancer cell lines (Caco-2, HT29, and HCT116) were determined using the MTT assay. Furthermore, as possible molecular targets, the ability of the most active hybrids to inhibit tubulin polymerisation and the catalytic activities of both topoisomerases I and topoisomerases II were examined. Also, the ability of the most promising hybrids to induce DNA double strand breaks and cell cycle arrest in HCT116 cancer cells in addition to their effect on the protein expression levels of γH2AX was evaluated. Moreover, the docking studies of new hybrids were investigated at the TopI (**1T8I**) and Top II (**6ZY7**) active sites. To the best of our knowledge, this is the first report that gathers these three important pharmacophores; Ciprofloxacin, Chalcone and 1,2,3-triazole in one compact unit aiming for synergistic anticancer activity through acting on different molecular targets (Figure 4).

## 2. Results and discussion

### 2.1. Chemistry

**Scheme 1.**
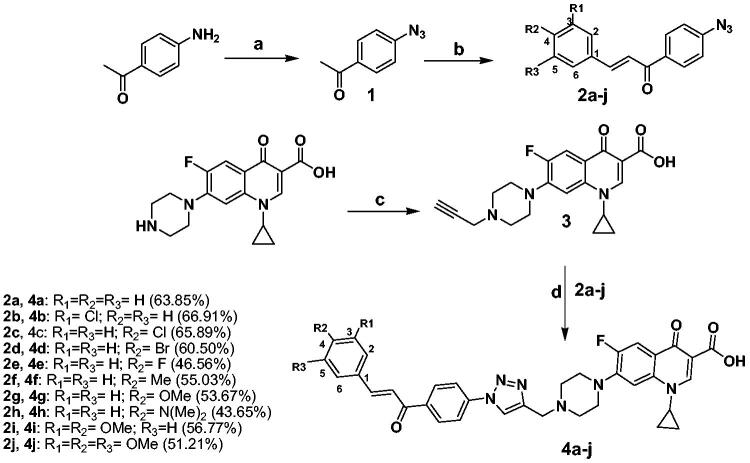
Reagents and conditions: (a) Conc. H_2_SO_4_, NaNO_2_, NaN_3_, 0–5 °C; (b) Proper aromatic aldehydes, 60% sodium hydroxide, ethanol, stirred overnight at 0–5 °C; (c) Propargyl bromide, NaHCO_3_, DMF, stirred overnight at 80 °C; (d) CuSO_4_·5H_2_O, Sodium ascorbate, DMF, CH_2_Cl_2_, MeOH, H_2_O (3:2:1:1) rt stirring 24 h.

Both new compounds and their intermediates were synthesised as drawn in Scheme 1. In particular, the intermediate 1-(4-azidophenyl)ethanone **1** was prepared following the previously published procedures^65^^,^^66^. Chalcone derivatives **2a-j** were synthesised by condensation of 1-(4-azidophenyl)ethanone **1** with the appropriate aromatic aldehyde in ethanol in presence of sodium hydroxide^65^^,^^66^. Compound **3** was prepared by alkylation of CP with propargyl bromide in dimethylformamide (DMF) in the presence of NaHCO_3_^67^_._ The final compounds **4a-j** were synthesised by coupling chalcone derivatives **2a-j** with CP derivative **3** in DMF using sodium ascorbate and copper sulphate as a catalyst. Structure elucidation of our target compounds was carried out via IR, ^1^H NMR, ^13 ^C NMR, elemental analysis, and mass spectrometry. The IR spectra of compounds **4a**, **4e**, and **4i** as representative examples showed a broad stretching bands at 3200–3048 cm^−1^ assigned to OH of the carboxylic group, in addition to three carbonyl stretching bands appeared at 1719–1715 cm^−1^, 1658–1657 cm^−1^, and 1625–1622 cm^−1^ assigned to C = O of the carboxylic, carbonyl of the chalcone and the 4-keto functionality, respectively. The pattern of parent CP and chalcone scaffolds was confirmed by ^1^H NMR. Chemical shifts (*δ*) at 1.19–1.35 ppm and 3.45–3.85 ppm (two multiplet signals) represent the protons of cyclopropyl and piperazine moieties, respectively. Also, aromatic protons (3) and carboxylic proton of CP appeared at *δ* = 7.56–8.66 ppm and 15.05–15.17 ppm, respectively. The two chalcone protons appeared at *δ* = 7.58–8.03 ppm. One characteristic singlet for the triazolyl *H* appeared at *δ* 8.83–8.93 ppm. Moreover, ^13 ^C NMR showed a characteristic signal at *δ* = 186.77–188.90 ppm which corresponds to the carbonyl group of the chalcone scaffold.

#### 2.2. Biological investigations

##### 2.2.1. Cytotoxic studies

###### 2.2.1.1. *In-vitro* single-dose experiment

For screening of anticancer properties, all the developed compounds were chosen by the (NCI) for *in-vitro* antitumor evaluation. The assay was carried out using 59 human cancer cell lines obtained from nine tumour subpanels (breast, ovarian, melanoma, leukaemia, renal, CNS, lung, prostate, and colon cancer cell lines). Compounds were assessed at a 10 µM concentration. The results of anticancer screening (as shown in Table 1 in Supplementary Data**)** indicated that all the synthesised CP derivatives produced remarkable anti-proliferative effects against leukaemia (RPMI-8226 and SR) and colon (HCT116) tumour cells with a growth percent ranged from 47.83 to −53.36%. Among the tested compounds, CP hybrid **4j** exhibited a strong and wide range of anti-proliferative activities in the majority of cancer cell lines with a growth percent ranged from 67.46 to −39.69%. Also, hybrids **4a, 4b, 4e, 4i** and **4j** produced significant anti-proliferative effect in several cancer cells, particularly in leukaemia (RPMI-8226) and colon (HCT116) cells with growth percent of 47.87, −14.59, −24.56, −24.42, −15.84, −9.74, −53.36, −10.26, −31.29, and −39.51%, respectively.

###### 2.2.1.2 *In-vitro* five-dose experiment

Showing promising results in the single-dose assay, compound **4j** was advanced for further investigation against a panel of 59 human cancer cell-lines at a five-dose levels. Those cell lines represent nine different tumours, and they were all incubated with our compound at 5 increasing concentrations (0.01, 0.1, 1, 10 and 100 µM). The *In-vitro* five-dose assay results were listed in Table 1, as GI_50_ which is the concentration causes 50% inhibition of cell proliferation and it was expressed in µM. Moreover, Total growth inhibition concentration (TGI) in µM was calculated and listed in Table 1. Results showed that compound **4j** displayed high activity and a wide range of antitumor effects with no selectivity towards all cancer cell lines with GI_50_ values between 0.21 µM and 21.10 µM with selectivity ratio from 0.67 to 1.69 µM at GI_50_ level, as shown in Table 1. Furthermore, compound **4j** displayed the capacity to achieve total growth inhibition (TGI) against thirty-one cell lines with TGI values between 0.54 and 45.20 µM.

**Table 1.** GI_50_ in µM and total growth inhibition (TGI) in µM of compound **4j** against 59 cell lines of nine cancer panels evaluated using NCI's *in-vitro* 5-dose antitumor assay.

Panel/Cell line	GI_50_ (µM)	TGI (µM)	Panel/Cell line	GI_50_ (µM)	TGI (µM)
Leukaemia			Melanoma		
CCRF-CEM	2.63	>100	LOX IMVI	1.57	nd
HL-60(TB)	3.18	>100	MALME-3M	2.59	8.41
K-562	0.97	>100	M14	2.49	nd
MOLT-4	3.32	>100	MDA-MB-435	1.49	4.67
RPMI-8226	0.40	4.46	SK-MEL-2	21.10	>100
SR	0.43	>100	SK-MEL-28	2.77	10.40
Non-small cell lung cancer			SK-MEL-5	2.64	7.74
A549/ATCC	2.98	>100	UACC-257	6.34	>100
EKVX	4.24	>100	UACC-62	2.83	8.84
HOP-62	2.49	>100		1.73	3.43
HOP-92	11.70	38.30	Ovarian Cancer		
NCI-H226	2.39	6.63	IGROV1	1.64	4.04
NCI-H23	2.97	22.30	OVCAR-3	1.84	4.93
NCI-H322M	3.58	>100	OVCAR-4	3.94	15.50
NCI-H460	2.40	6.66	OVCAR-5	3.70	25.70
NCI-H522	4.25	>100	OVCAR-8	4.03	>100
Colon cancer			NCI/ADR-RES	>100	>100
COLO 205	5.08	>100	SK-OV-3	7.09	>100
HCC-2998	1.73	4.08	Renal Cancer		
HCT-116	0.21	0.54	786-0	1.67	3.39
HCT-15	2.25	>100	A498	4.18	14.40
HT29	1.24	>100	ACHN	3.50	45.20
KM12	1.57	4.46	CAKI-1	5.10	19.00
SW-620	1.37	6.98	SN12C	2.66	10.00
CNS cancer			TK-10	5.61	>100
SF-268	3.09	>100	UO-31	1.73	9.01
SF-295	2.91	>100	Breast cancer		
SF-539	1.91	4.39	MCF7	0.39	>100
SNB-19	4.52	0.67	MDA-MB231/ATCC	3.20	>100
SNB-75	1.49	4.29	HS 578 T	3.91	>100
U251	1.48	>100	BT-549	1.71	3.76
Prostate cancer			T-47D	3.16	>100
PC-3	2.54	15.70	MDA-MB-468	1.96	5.24
DU-145	3.67	34.90			

###### 2.2.1.3. *In-vitro* anti-proliferative study using HCT116, HT29, and Caco-2 cells

As the one dose experiment showed remarkable growth inhibition of the synthesised compounds against the colon cancer cells, we determined the IC_50s_ of the target hybrid compounds **4a-j** against three human colon cancer cell lines (Caco-2, HT-29, and HCT116) using the MTT assay after 24 h incubation period. Results in Table 2 showed that the new CP hybrids **4a-j** had stronger anti-proliferative effects in HCT116 cells (IC_50_ range 2.53–8.67 µM) than H29 cells (IC_50_ range 8.67–62.47 µM) and Caco-2 cells (IC_50_ range 4.19–24.37 µM). Compounds **4a**, **4b**, **4e**, **4i**, and **4j** displayed noticeable anti-proliferative effects against the colon cancer cells. Among the tested compounds, the unsubstituted (**4a**) and 3,4,5-trimethoxy (**4j**) derivatives exhibited the strongest anti-proliferative effect. Also, the results indicated that biological activity is not affected by the electronic effect of the substituents. However, the **4j** showed the highest cytotoxic effect against HCT116 cells, we noticed difference in the IC_50_ values of **4j** between using the SRB assay (IC_50_ = 0.2 µM) and the MTT assay (IC_50_ = 2.53 µM). We assume this difference is due to the SRB assay is more sensitive than the MTT assay as previously described in the literature^68^^,^^69^. Simple structure-activity relationship (SAR) based on HCT-116 cytotoxicity data showed that the absence of substituents on the aromatic ring is favourable to any substituted except 3,4,5-trimethoxy substituents (compound **4j**). For monosubstituted derivatives, the fluorine atom in position 4 is optimal for activity over other donating groups or halogens at the same position. Additionally, the anti-proliferative activity increased with decreasing the halogen size as follows 4-Br < 4-Cl < 4-F.

**Table 2:** Cytotoxic effects of CP hybrids, **4a-j** and doxorubicin against 3 colon cancer cell lines (Caco-2, HT-29, and HCT116).

Compound	HCT116	HT-29	Caco-2
**4a**	3.57 ± 0.59	22.35 ± 3.25	8.39 ± 2.46
**4b**	4.81 ± 1.21	42.91 ± 5.14	24.37 ± 3.07
**4c**	6.45 ± 1.84	15.34 ± 1.54	11.73 ± 2.95
**4d**	8.67 ± 2.15	62.47 ± 7.10	7.19 ± 1.69
**4e**	4.32 ± 0.98	8.67 ± 2.94	4.19 ± 1.13
**4f**	7.22 ± 1.45	14.71 ± 1.81	10.05 ± 2.82
**4g**	6.11 ± 1.03	34.01 ± 3.69	11.41 ± 3.19
**4h**	5.09 ± 2.74	21.74 ± 2.74	6.39 ± 1.41
**4i**	4.87 ± 0.78	18.60 ± 3.19	11.28 ± 2.39
**4j**	2.53 ± 0.31	13.24 ± 2.40	7.14 ± 2.48
Doxorubicin	1.22 ± 0.27	0.88 ± 0.58	4.15 ± 1.08

###### 2.2.1.4. Effect of ciprofloxacin-chalcone hybrids on non-cancerous cells

**Figure 5.**
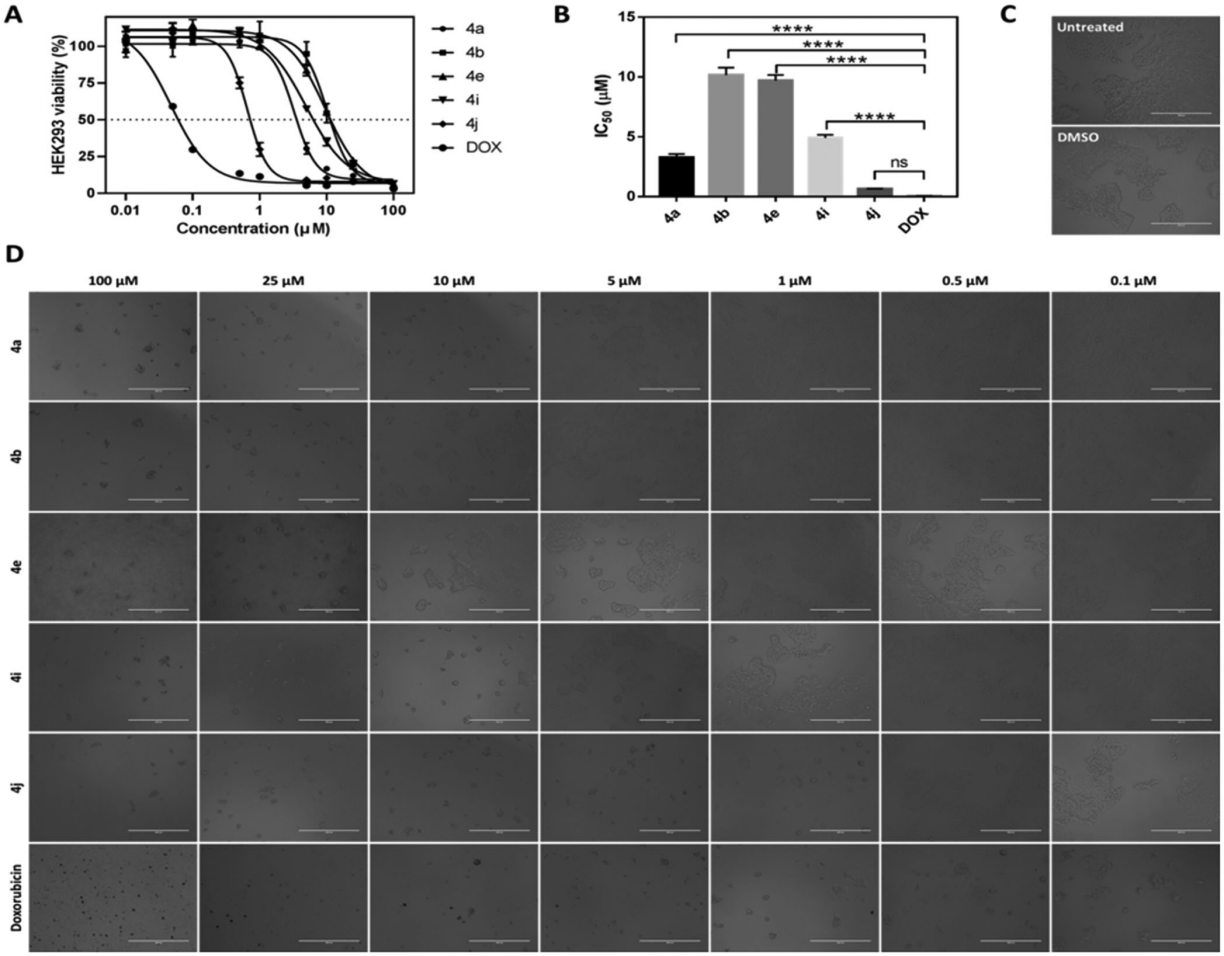
Cytotoxic effects of CP hybrids (**4a**, **4b**, **4e**, **4i**, and **4j**) and doxorubicin against non-cancerous cells (HEK293) after 72 h incubation period. **(**A) Concentration-cell viability curves of CP hybrids and doxorubicin treated HEK293 cells using different concentrations (0.01–100 µM, *n* = 3) and 72 h incubation period. (B) IC_50_ values were estimated by fitting the cytotoxicity data into a linear regression model using GraphPad Prism software. Data were statistically compared by one-way ANOVA followed by Tuckey’s multiple comparisons test (****p < 0.0001, ns = not significant). (C and D). Microscopic images of HEK293 cells treated with different concentrations of CP hybrids (**4a**, **4b**, **4e**, **4i**, and **4j**) and doxorubicin for 72 h (20× objective lens, total magnification = 200×). Scale bar = 400 µM.

To evaluate the selectivity and safety profile of the synthesised CP hybrids, a cytotoxicity experiment of compounds **4a**, **4b**, **4e**, **4i**, and **4j** was performed using normal Human Embryonic Kidney (HEK) 293 cells. Results obtained from cytotoxicity assay showed that CP hybrids **4a**, **4b**, **4e**, **4i**, and **4j** caused cytotoxic effects in HEK293 cells to different extents after 72 h incubation period with IC_50_s ranging from 0.6523 µM to10.1600 µM, but their cytotoxicity were much lower than that of doxorubicin cytotoxicity in HEK293 which had IC_50_ equal to 0.0475 µM (Figure 5(A)). CP hybrid **4j** had the steepest concentration-cell viability curve of any other ciprofloxacin-chalcone hybrids against HEK293 cells, suggesting that the **4j** compound had stronger cytotoxic activity than other ciprofloxacin-chalcone hybrids. However, the concentration-cell viability curve of **4j** was shallower than the dose-response curve of doxorubicin (positive control). This demonstrates that our synthesised CP hybrids are less cytotoxic against non-cancerous cells (HEK293) when compared to the commonly used chemotherapy (doxorubicin). Further analysis of the cytotoxicity data revealed that CP hybrids (**4a**, **4b**, **4e**, and **4i**) had significantly larger IC_50_ values (*p* ≤ 0.0001, Figure 5(B)). Also, the **4j** compound had a higher IC_50_ value than doxorubicin albeit not significantly different. These findings were further supported by microscopic images of cells treated with different concentrations of these compounds (Figure 5(C,D)). The cytotoxic effects of CP hybrids in HEK293 may be due to the long incubation period compared to the incubation period of HCT116 cells. Furthermore, compound **4j**, a more cytotoxic hybrid, had a selectivity index six-fold better than that of the drug Doxorubicin.

#### 2.2.2. Effects of compounds 4a, 4b, 4e, 4i, and 4j on topoisomerase I/II inhibitory activity

**Figure 6.**
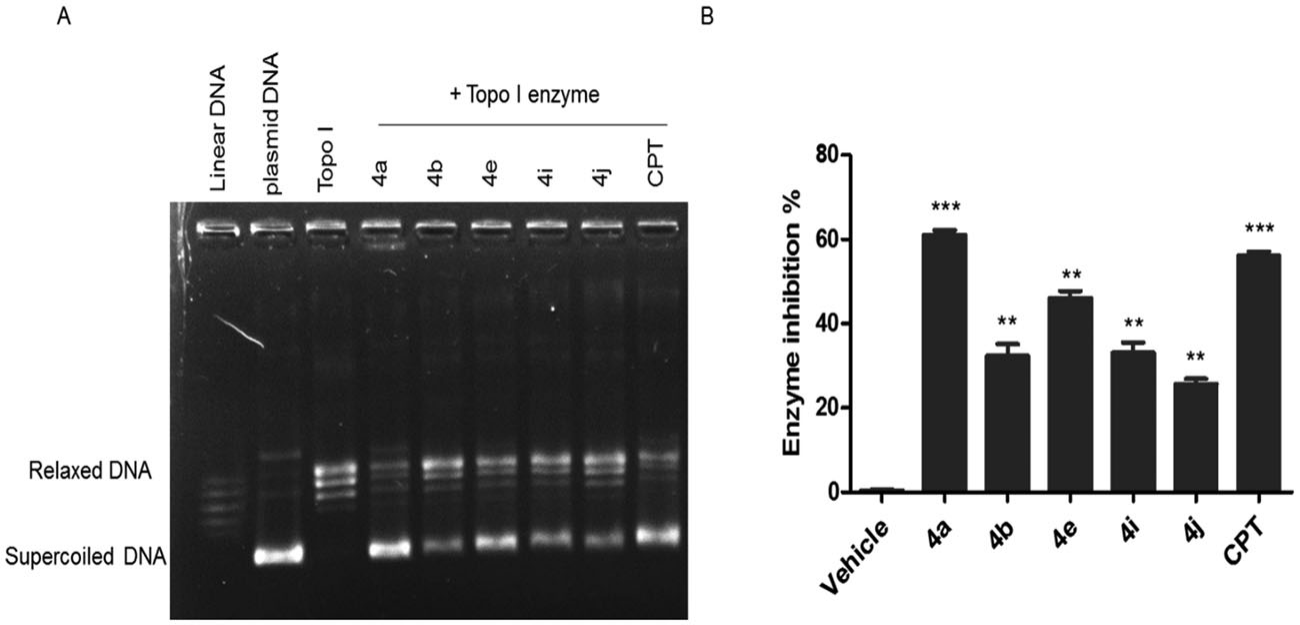
The effect of IC_50_ values of CP hybrids **4a, 4b, 4e, 4i, 4j** and camptothecin (CPT, positive control in Topo-I assay**)** on the activity of human topoisomerase I. (A) DNA relaxation assay. (B) *in-vitro* enzyme assay. Data are plotted as means ± SEM of 3 experiments. ^∗∗^ *p* < 0.01 and ^∗∗∗^ *p* < 0.001 refer to the statistically significant differences in comparison to DMSO (vehicle, negative control treatment).

Based on our design, we hypothesised that the new CP hybrids **4a-j** could act as multi-target ligands that can block the catalytic activity of topoisomerases I (Topo-I), topoisomerases (Topo-II), and inhibit tubulin polymerisation as possible targets for the anti-proliferative activity. First, the Topo-I activity was tested by the DNA relaxation assay. The supercoiled plasmid has been utilised as a substrate in the relaxation assay to study the inhibition of DNA Topo-I activity. The relaxed isomers migrate in the gel more slowly than the supercoiled isomers; hence they can be distinguished from each other. If the synthesised compounds have an inhibitory effect on the catalytic activity of Topo-I, the DNA molecules will remain in the supercoiled form, a faster moving single band. As shown in (Figure 6(A)), we determined that **4a**, **4b**, **4e**, **4i**, and **4j** could inhibit Topo-I catalytic activity, with **4a** the most potent Topo-I inhibitor.

Moreover, the Topo-I enzymatic activity inhibition by the synthesised compounds was confirmed using the Human DNA Topoisomerase I Assay Kit (ProFoldin, Hudson, MA, USA) (Figure 6(B)). The results of Human DNA Topoisomerase I Assay as shown in (Figure 6(B)) indicated that compound **4a** had the highest Top-I inhibitory activity which was slightly higher than that of the reference camptothecin. On the other hand, compound **4j** was the less potent Top-1 inhibitor.

The remarkable Top-I inhibitory activities of the synthesised hybrids; **4a**, **4b**, **4e**, **4i**, and **4j** are in agreement with our hypotheses of these hybrid as Top-I inhibitors.

**Figure 7.**
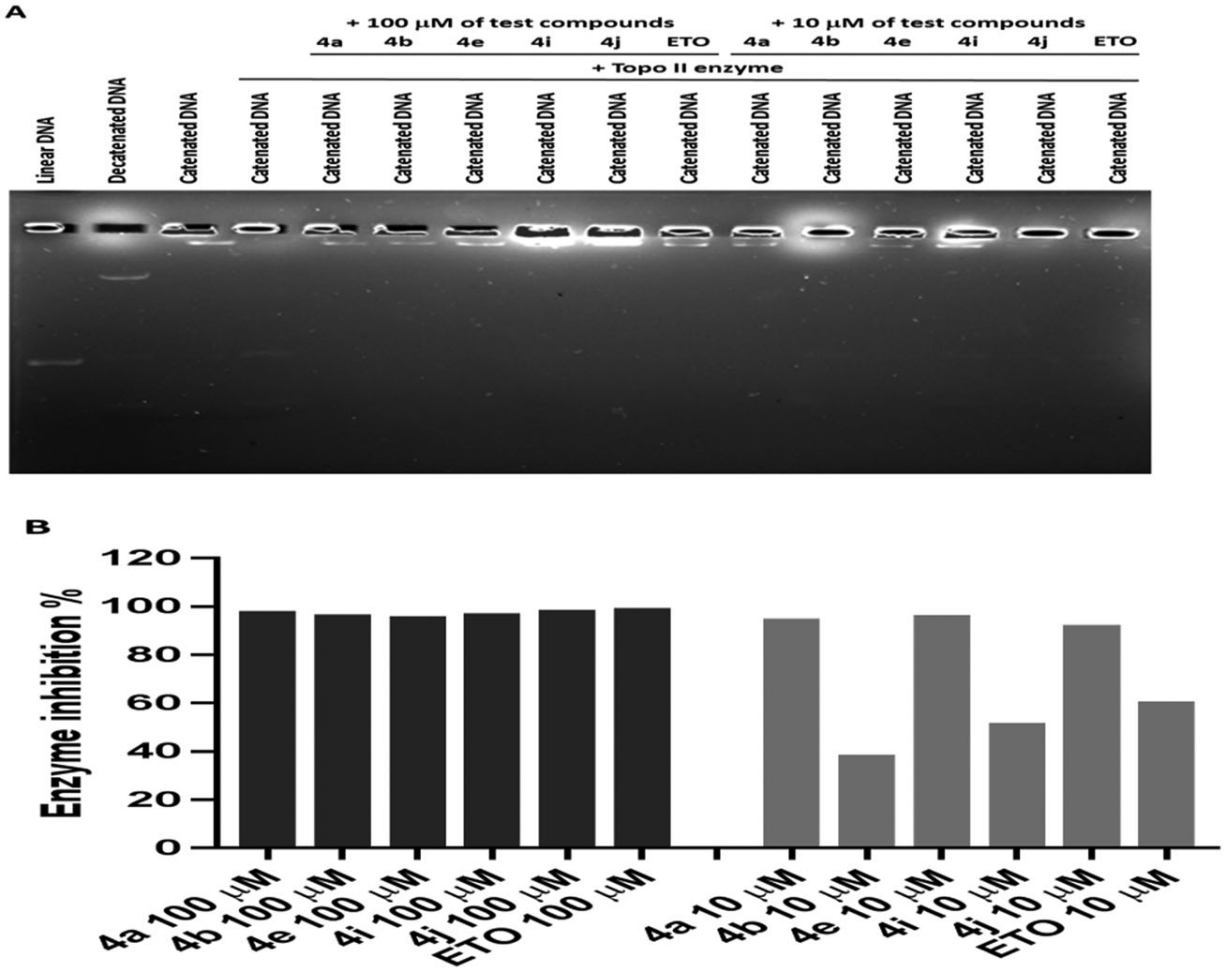
The effect of ciprofloxacin hybrids **4a, 4b, 4e, 4i, 4j**, and **etoposide** (**ETO**, positive control in Topo-II assay) on the activity of human topoisomerase II. (A). Agarose gel electrophoresis of human topoisomerase II assay. (B). Topo-II inhibitory activity of ciprofloxacin hybrids.

Second, the inhibitory activity of Topo IIα was evaluated for compounds **4a**, **4b**, **4e**, **4i**, and **4j** utilising Human Topoisomerase II assay kit (TG1001, Topogen Inc.). Etoposide, known inhibitor of Topo IIα, was used as reference.Top-II assay results indicated that the synthesised CP hybrids exhibited strong Topo-II inhibitory activity when compared to the positive control, etoposide (ETO) (Figure 7(A)). Based on the analysis of band intensity using ImageJ software, it was found that all CP hybrids inhibited Topo-II to a similar magnitude in comparison to ETO when 100 µM of each compound was used (Figure 7(B)). However, when a lower concentration (10 µM) was tested, it was found that three ciprofloxacin hybrids (**4a, 4e**, and **4j**) had stronger inhibitory activity than **4b**, **4i**, and ETO. The above results are compatible with our design of these hybrids as Top-II inhibitors. According to these results, CP hybrid **4a** had the strongest inhibitory activity on both Topo-I and Topo-II enzymes.

#### 2.2.3. Effects of compounds 4a, 4b, 4e, 4i, and 4j on tubulin polymerisation

**Figure 8.**
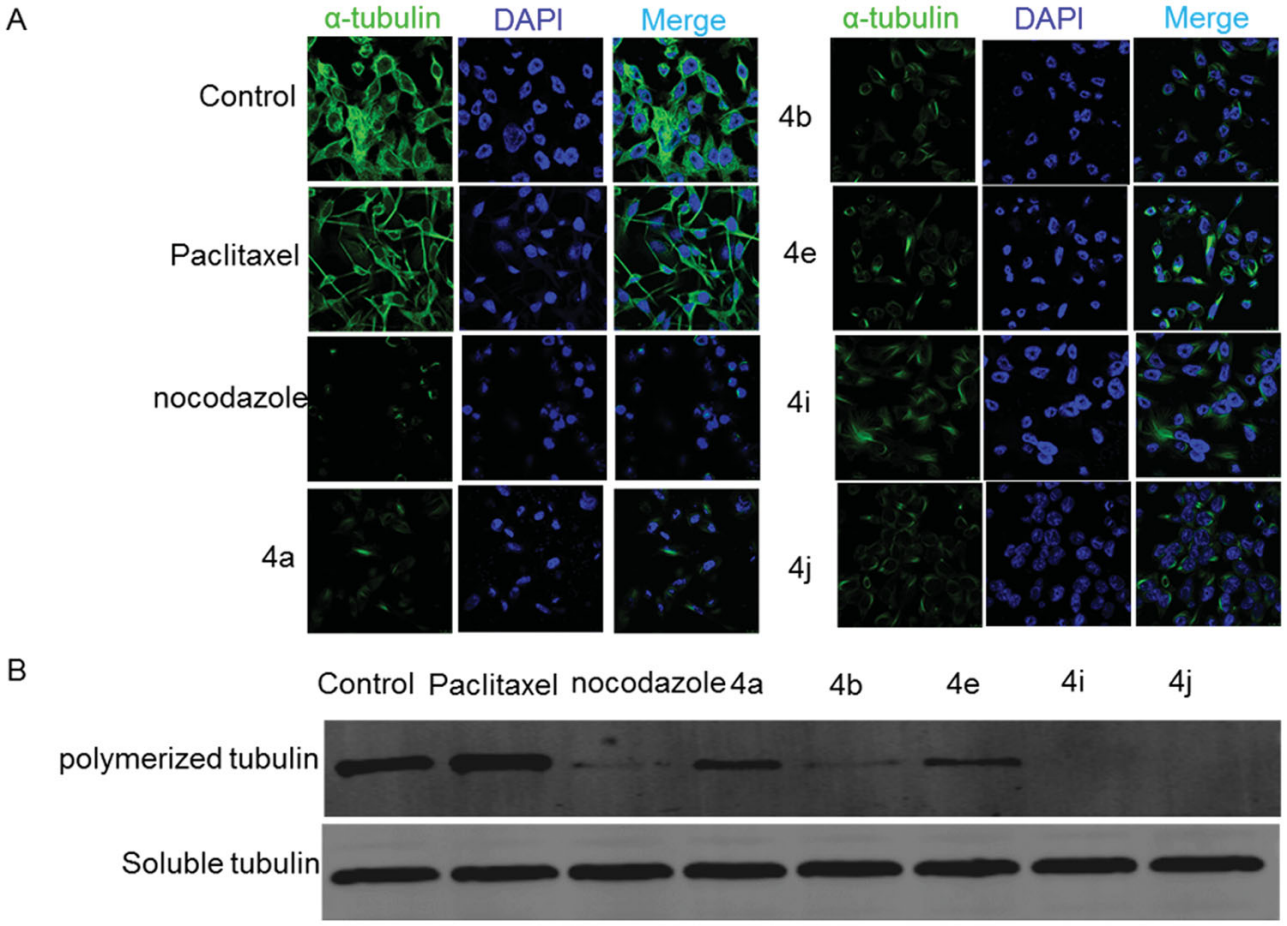
Effect of ciprofloxacin derivatives on tubulin polymerisation. HCT116 cells were untreated (control) and treated with IC_50_ values of compounds **4a, 4b, 4e, 4i, 4j**, paclitaxel or nocodazole for 36 h. (A) Morphological changes of HCT116 cells visualised by immunofluorescence microscopy. Tubulin were shown in green and the nuclei were in blue. (B) Western blotting analysis.

The process of tubulin polymerisation in the cells is important for the completion of mitosis and is targeted for cancer treatment. It has been reported that the suppression of tubulin polymerisation will arrest the cells at the G2/M and it eventually leads to cell death. To test the tubulin-disruptive effect of the CP hybrids, HCT116 cells were analysed by immunocytochemistry. HCT116 cells were incubated for 36 h with IC_50_ of the CP hybrids (**4a**, **4b**, **4e**, **4i**, and **4j)**, nocodazole (a well-known tubulin polymerisation inhibitor, as a positive control), DMSO or Paclitaxel (a well-known tubulin polymerisation inducer, as a negative control), and tubulin was visualised by immunofluorescence microscopy. An antibody for α-tubulin was used. Untreated and Paclitaxel treated HCT116 cells showed a spider-web-like structure (Figure 8). Morphological changes of HCT116 cells treated with either CP hybrids (**4a**, **4b**, **4e**, **4i**, and **4j)** or nocodazole showed a significant tubulin polymerisation inhibition (Figure 8(A)). Moreover, a Western blot was performed to verify the tubulin polymerisation inhibition ability of the synthesised compounds (Figure 8(B)). The level of polymerised tubulin was decreased in HCT116 cells treated with IC_50_ values of CP hybrids **4a**, **4b**, **4e**, **4i**, **4j**, or nocodazole. Additionally, compounds **4i** and **4j** showed potent inhibition of tubulin polymerisation (Figure 8) and a similar weak effect on human topoisomerase I activity which may suggest that the tubulin inhibition mechanism is the primary mechanism of action for these compounds. The above-mentioned findings indicated that the synthesised compounds could act as multi-targets; Topo-I, TopII, and tubulin polymerisation inhibitors as intended by our design.

#### 2.2.4. DNA damage induction upon treatment with compounds 4a, 4b, 4e, 4i, and 4j

**Figure 9.**
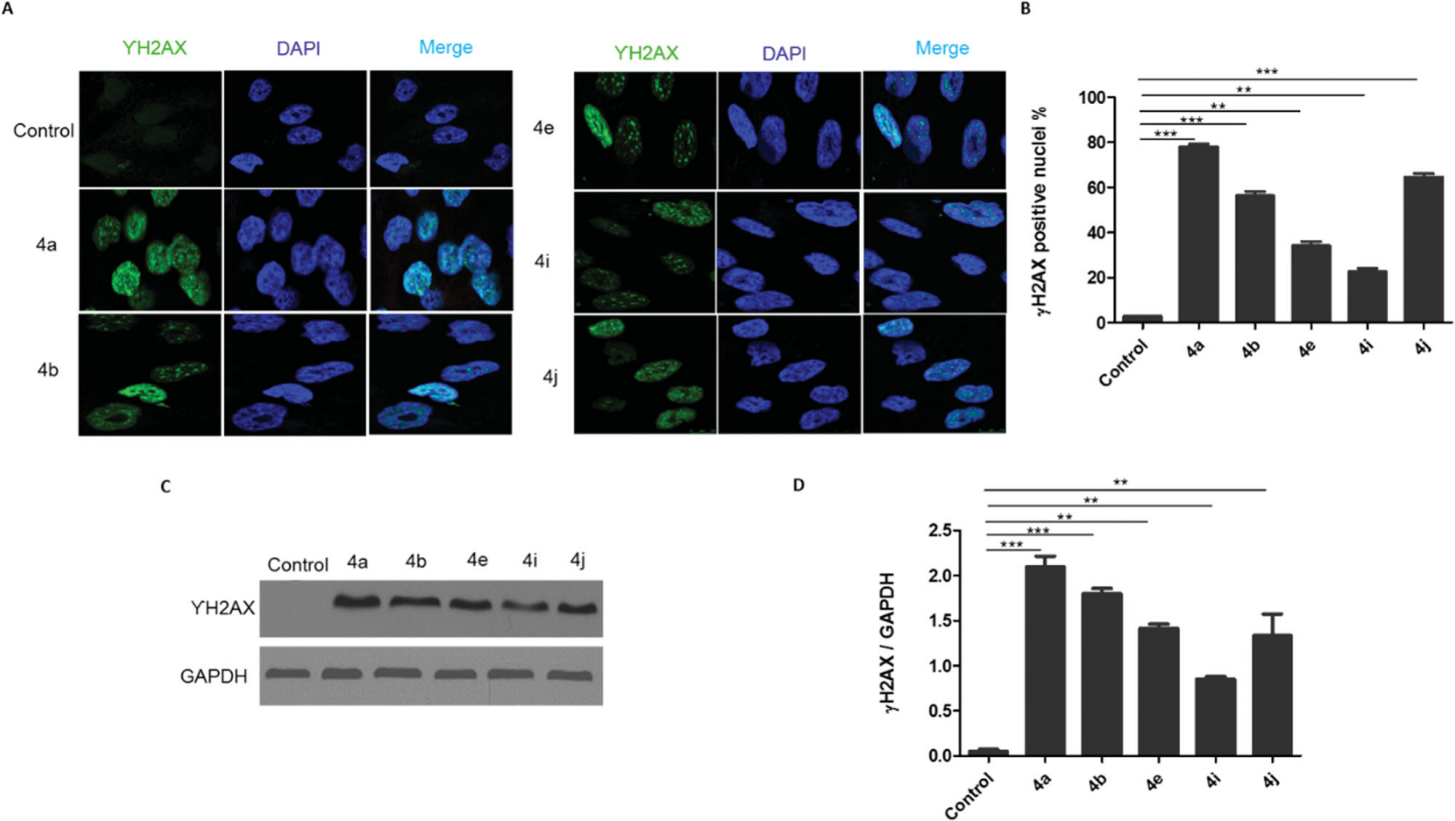
CP hybrids induce DNA damage. (A) γH2AX staining in HCT116 cells treated with IC_50_ values of CP hybrids **4a, 4b, 4e, 4i, 4j, or DMSO** (negative control). (B) γH2AX-positive nuclei %. (C) Western blot revealed that levels of γH2AX were increased in HCT116 cells treated with CP hybrids **4a, 4b, 4e, 4i, 4j, DMSO** (negative control), and GAPDH (loading control). (D) The relative protein level of γ-H2AX, in comparison to GAPDH was measured by ImageJ. Data are plotted as means ± SEM of 3 experiments. ^∗^ *p* < 0.05, ^∗∗^ *p* < 0.01, and ^∗∗∗^ *p* < 0.001 refer to the statistically significant differences in comparison to DMSO.

DNA damage is a major reason for inhibiting proliferation and inducing cytotoxicity in cancer cells as well as the main downstream cascade for the Topo-I, Top-II, and tubulin polymerisation inhibition. The phosphorylation of the histone H2AX always occurs after DNA damage forming double-stranded breaks (DSBs). Therefore, we tested the capability of the potent prepared compounds to trigger DNA double strand breaks through detection of the phosphorylation of γH2AX using two different techniques, Immunofluorescence, and western blotting. Results in (Figure 9(A,B)) indicated that treatment of HCT116 cells with the IC_50_ of the potent compounds (**4a**, **4b**, **4e**, **4i**, and **4j**) induced γH2AX foci formation. Also, more γH2AX-positive cells were observed in comparison to the control cells. Similarly, the western blotting analysis (Figure 9(C,D)) showed increases in the γH2AX protein expression in cells treated with the IC_50_ of the target compounds (**4a**, **4b**, **4e**, **4i,** and **4j**) in comparison with the control cells. These results clearly show that the 1,2,3-Triazole linked CP hybrids induce DNA damage in colon cancer cells.

#### 2.2.5. Effects of compounds 4a, 4b, 4e, 4i, and 4j on cell cycle analysis

**Figure 10.**
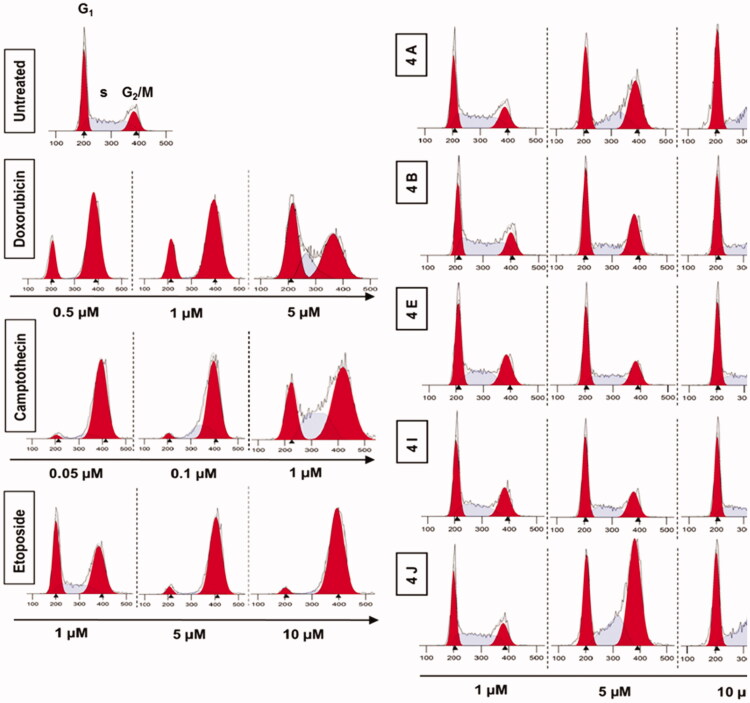
Analysis of cell cycle. HCT116 cells were treated with different concentrations of ciprofloxacin hybrids **4a, 4b, 4e, 4i**, and **4j** for 24 h. Doxorubicin, camptothecin, and etoposide were used as assay controls. The X-axis displays the fluorescence intensity corresponding to the DNA content per HCT116 cell. DNA histograms were analysed by ModFit LT software.

Generally, the mammalian cells counteract the DNA damage by different mechanisms including the cell cycle arrest to allow the cell to repair the damage. There are 2 checkpoints that control DNA damage. The first checkpoint is at the G1/S transition while the other checkpoint is at the G2/M transition. To cover the mechanisms of the synthesised compounds in inhibiting cell proliferation, we analysed the distribution of cell cycle of HCT116 cells following the treatment with different doses of **4a**, **4b**, **4e**, **4i**, and **4j**. The result showed that **4a**, **4b**, **4e**, **4i**, and **4j** induced G2/M phase cell cycle arrest in a concentration-dependent manner (Figure 10). Our results suggest that 1,2,3-Triazole linked CP-Chalcone hybrids arrest the cell cycle of colon tumour cells in G2/M phase.

#### 2.2.6. Effects of compounds 4a, 4b, 4e, 4i, and 4j on activation of the ATR-Chk1-Cdc25C Cascade

**Figure 11.**
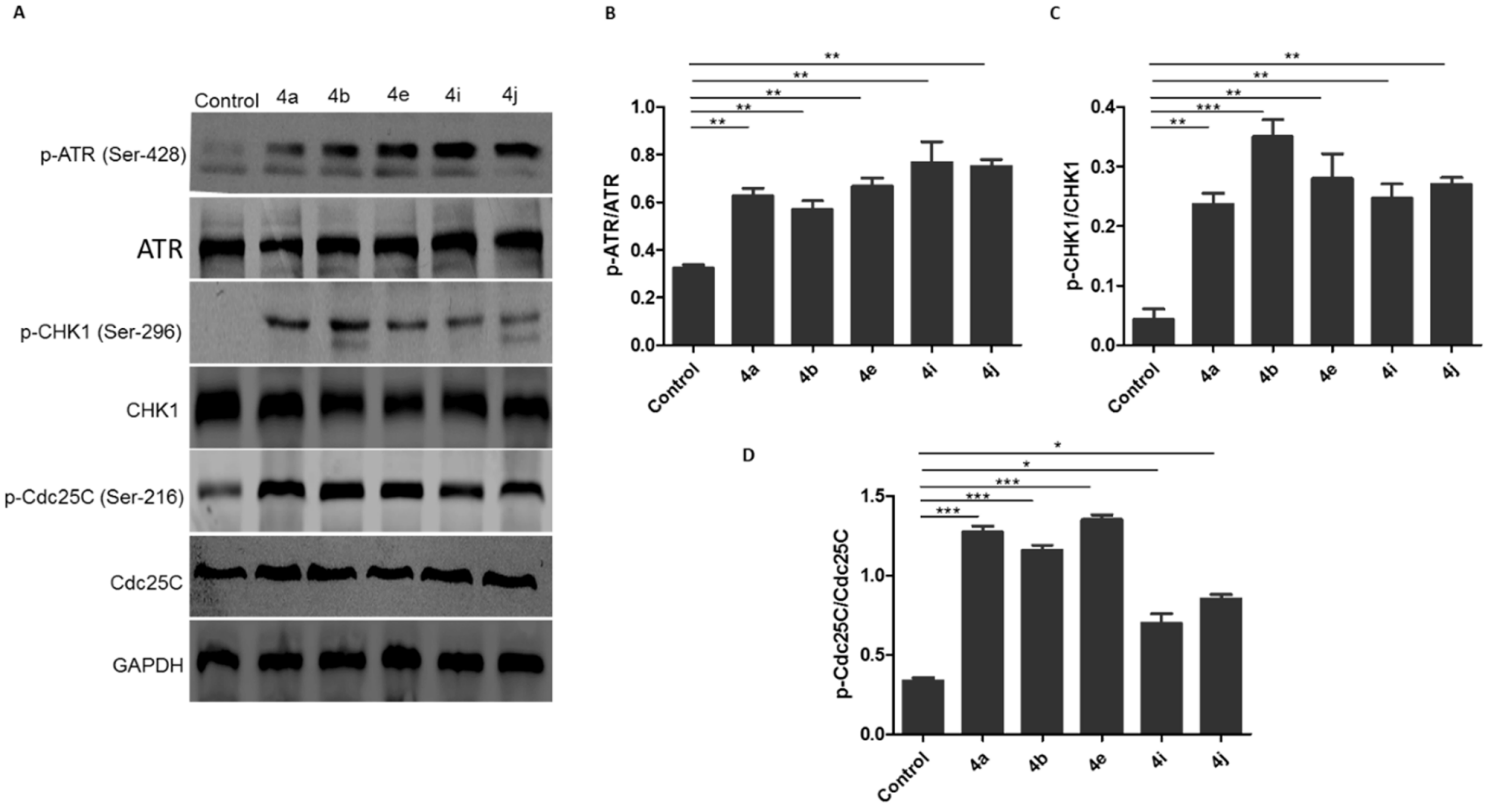
Ciprofloxacin hybrids **4a, 4b, 4e, 4i, and 4j** induce G2/M cell cycle arrest possibly via ATR/CHK1/Cdc25C pathway A. Western blot revealed that expressions of p-ATR, p-CHK1, and p-Cdc25C were increased in HCT116 cells treated with Ciprofloxacin hybrids **4a, 4b, 4e, 4i, 4j, DMSO** (negative control), and GAPDH (loading control) B. The relative protein levels of p-ATR, p-CHK1, and p-Cdc25C, compared to their total form ATR, CHK1, and Cdc25C, respectively, were measured by ImageJ. Data are plotted as means ± SEM of 3 experiments. * p < 0.05, ** *p* < 0.01, and *** *p* < 0.001 refer to the statistically significant differences in comparison to DMSO.

To clarify the molecular mechanism responsible for inducing G2/M phase arrest in HCT116 following the treatment with the synthesised compounds, the signalling in the checkpoint pathway that regulates the G_2_/M transition was investigated. The mammalian cells respond to the DNA damage through 2 different kinase-signalling cascades (ATR-Chk1 and ATM-Chk2). First, the effect of the synthesised compounds on ATM and ATR phosphorylation was tested. It was found that the ATM phosphorylation on Ser-1981 was unchanged in the comparison with the control (data not shown) after treatment with the synthesised compounds, while p-ATR at Ser-428 markedly increased (Figure 11(A,B)). Then, the phosphorylation of downstream effectors of ATM/ATR, phospho-Chk1-S269, and phospho-Chk2-T68 kinases was explored. It was observed that the synthesised compounds upregulated the phosphorylation of Chk1 at S269 (Figure 11(A,C)) without affecting the phosphorylation of Chk2 at Thr-68 (data not shown). It is well known that *in vitro* activation of Chk1 phosphorylates Cdc25C on serine-216. Therefore, the impact of the synthesised compounds on the Cdc25C phosphorylation at Ser-216 was investigated. Results showed that the levels of Cdc25C phosphorylation at Ser-216 were substantially elevated in triazole-treated HCT116 cells (Figure 11(A,D)). Overall, these results show that the synthesised hybrids induce G_2_/M cell cycle arrest possibly via ATR/CHK1/Cdc25C pathway.

### 2.3. Molecular docking studies of ciprofloxacin hybrids 4a, 4b, 4e, 4i, and 4j on Top-I and Top-II active sites

#### 2.3.1. Docking Studies of ciprofloxacin hybrids 4a, 4b, 4e, 4i, and 4j in human Topo I enzyme (PDB ID: 1T8I)

Based on the potent inhibitory Top-I activities of the synthesised hybrids, we study *in silico* molecular docking simulations using MOE software for the most active compounds **4a, 4b, 4e, 4i, and 4j** into human Top-I enzyme (PDB ID: **1T8I**) in comparison to camptothecin to examine their binding modes. The validation of the docking method was made by re-docking of the co-crystallized ligand and calculation of RMSD. The RMSD was 0.4626 which means the validity of the docking method. All the tested hybrids had a strong binding affinity to the enzyme as the binding free energy (ΔG) values ranged from −10.905 to −13.267 Kcal/mole which were higher than that of camptothecin (ΔG = −9.761 Kcal/mole), (Table 3).

**Table 3.** **ΔG** values of the tested compounds **‎4a, 4b, 4e, 4i, 4j,** and camptothecin in the active binding site of Topo I enzyme (PDB ID**: 1T8I**).

Compound	ΔG values (kcal/mol)
**4a**	−10.905
**4b**	−11.622
**4e**	−11.154
**4i**	−11.738
**4j**	−13.267
Camptothecin	−9.761

**Figure 12.**
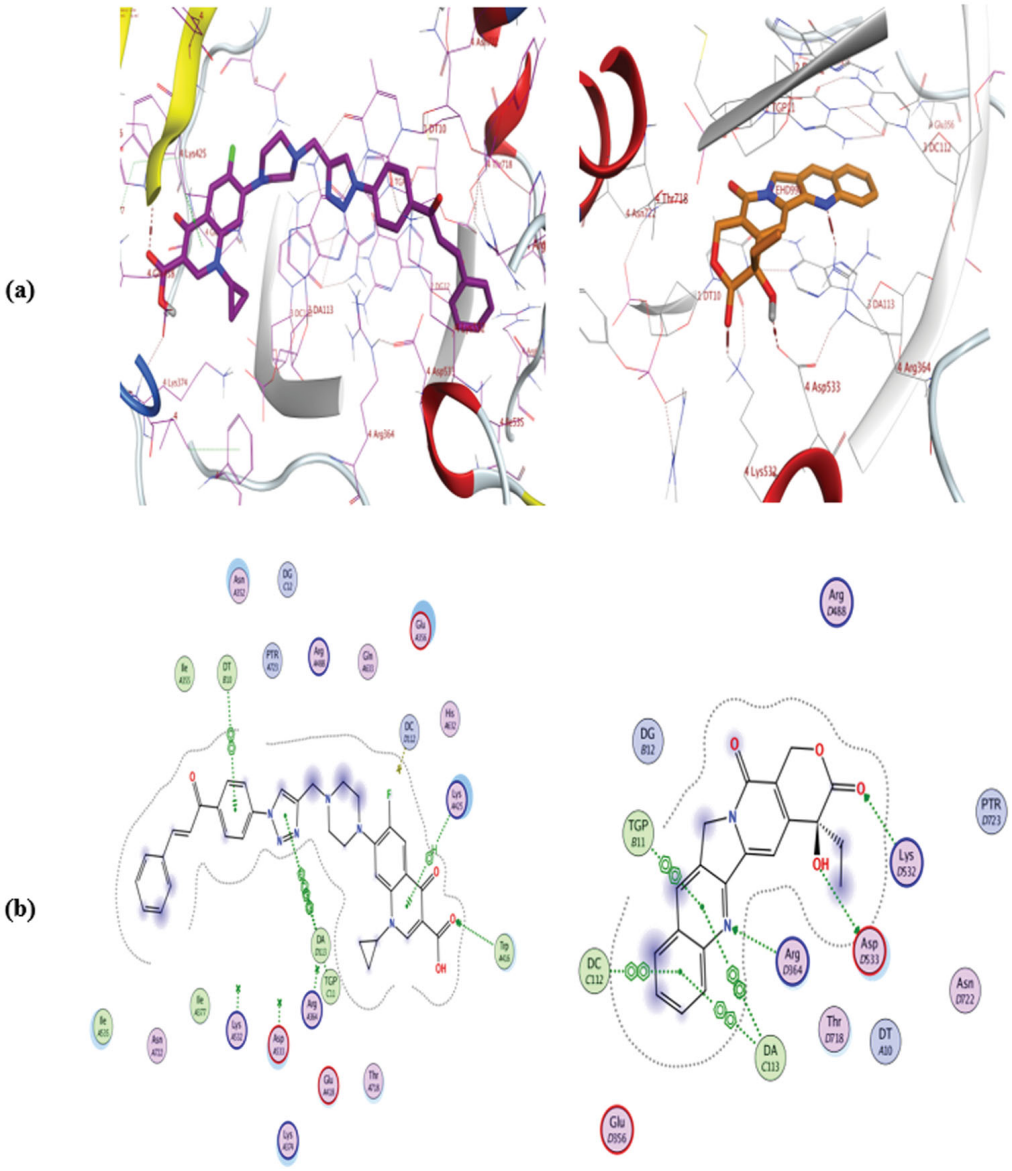
Binding mode and H-bonds interactions of compound **4a** (left) and camptothecin (right) within ‎**1T8I** ‎active site: (a) 3 D structure and (b) 2D interactions.

The Docking results (as shown in Table 2 and Figures 1–3, in Supplementary Data) are in agreement with Top-1 inhibitory activity results and indicate that compounds **4a**, **4e**, and **4i** have comparable binding interactions with Top-I enzyme (PDB ID: **1T8I**) to that of camptothecin which forms 3 H-bond interactions with Asp533, Lys532, and Arg364 and four π…π interactions with DA 113, DC 112, and TGP 111, (Figure 12). On the contrast, compounds **4b** and **4j** have fewer binding interactions with Top-I enzyme (PDB ID: **1T8I**) than that of camptothecin. Concerning compound **4a** which had the most potent Top-I inhibitory activity even higher than that of camptothecin, its docking pose shows one H-bonding interaction with Trp 416, one cation…π interaction with Lys 425 and three π…π interactions with DT 10, TGP 111, and DA 113, as shown in (Figure 12). Additionally, compound **4e**, which had Topo-I inhibitory activity comparable to camptothecin, has two H-bonding interactions with Arg 488 & His 632 and three π…cation interactions with Trp416, DT 10, and DG 12. On the other side, compound **4j** which was the less potent Top-1 inhibitor among the tested hybrids, it has only one cation…π interaction with Glu 356 and one π…π interaction with DC112. See Supplementary Data. The above docking results indicated that although some of the new hybrids have comparable binding interactions as camptothecin, none of these new compounds have H-bond interactions with the same amino acid residues or have three H-bonds like camptothecin, Additionally, none of them have the same number of π…π interactions with the same DNA residues of Top-I enzyme such as camptothecin even the hybrid **4a** which was more potent as Top-1 inhibitor than camptothecin which means these hybrids could have a slight distinct interactions with Top-I enzyme.

#### 2.3.2. Docking studies of ciprofloxacin hybrids 4a, 4b, 4e, 4i, and 4j in human Topo II enzyme (PDB ID: 6ZY7)

Upon relying on the results of the Top II assay, docking of the most active hybrids; **4a, 4b, 4e, 4i,** and **4j**, and the positive control Etoposide; was performed with the active binding site of into human Topo II enzyme (PDB ID: **6ZY7**) to elucidate their effective binding modes and concentrate on their similarity to the standard ligand binding modes. The docking method validation was examined by re-docking of the co-crystallized Etoposide and calculation of RMSD. The RMSD was 0.4063 which means the docking method is valid. All the tested hybrids had a strong binding affinity to the enzyme as the binding free energy (ΔG) values ranged from −9.511 to −9.906 Kcal/mole which were lower than that of Etoposide (ΔG = −10.162 Kcal/mole), (Table 4).

**Table.4.** **ΔG** values of the tested compounds **‎4a, 4b, 4e, 4i, 4j,** and Etoposide in the active binding site of Topo II enzyme (PDB ID**: 6ZY7**).

Compound	ΔG values (kcal/mol)
**4a**	−9.846
**4b**	−9.738
**4e**	−9.511
**4i**	−9.690
**4j**	−9.906
Etoposide	−10.162

**Figure 13.**
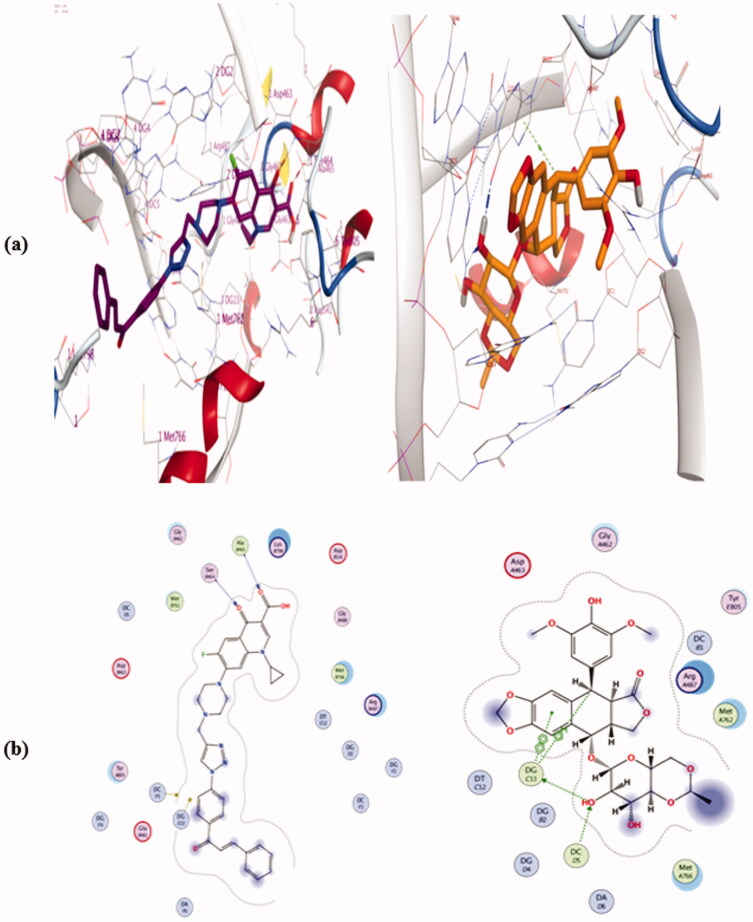
Binding mode and H-bonds interactions of compound **4a** (left) and etoposide (right) within ‎**6ZY7** ‎active site: (a) 3 D structure and (b) 2 D interactions.

From the docking, results (as shown in Table 3 and Figures 4–6, in Supplementary Data), we can say that the tested hybrids form different interactions than that of Etoposide which forms four interaction; two hydrogen bonds with DG 13 & DG 5 residues, one π…cation interaction with DG13, and one π…π interaction with DT 12, (Figure 13). Despite of compounds **4a**, **4e**, and **4j** were more potent as Top-II inhibitors than etoposide at 10 µM concentrations. They interact with the **6ZY7** binding site in a different manner with different amino acid residues than that of etoposide. For example, compound **4a** forms only two hydrogen bonds with Ala 465 and Ser 464, (Figure 13). Additionally, compound **4e** docking pose shows two hydrogen bonds with Ala 801 & Ser 802 and two π…cation interactions with DT 12 and DG 13. Moreover, **4j** forms only three interactions with the **6ZY7** binding site; two hydrogen bonds with Ser 800 and DC 3 residue and one π…cation interaction with DC 1 residue. On the other hand, compound **4b** which was less potent Top-II inhibitor among the tested compound at 10 µM forms only one hydrogen bond with Lys- 489.

## 3. Conclusions

We were successfully able to synthesise novel triazole-linked CP chalcone hybrids **4a-j** with remarkable anti-proliferative activity against leukaemia (RPMI-8226 and SR) and colon (HCT116) tumour cells with a growth percent ranged from 47.87 to −53.36%. Among the tested compounds, CP hybrid **4j** displayed potent and wide-range anti-proliferative effects in the majority of cancer lines with a growth percent range of 67.46 to −39.69%. Also, hybrids **4a, 4b, 4e, 4i,** and **4j** induced significant anti-proliferative effect in several cancer cells, particularly in leukaemia (RPMI-8226) and colon (HCT116) cell lines with a growth percent values 47.87, −14.59, −24.56, −24.42, −15.84, −9.74, −53.36, −10.26, −31.29, and −39.51%, respectively. Generally, the anti-proliferative activity of hybrids **4a-j** was more potent against colon cancer (HCT116) than leukaemia SR (RPMI-8226 and SR) cell lines. Compounds **4a, 4b, 4e, 4i,** and **4j** displayed the strongest anti-proliferative effect in HCT116 cells with IC_50_ = 3.57, 4.81, 4.32, 4.87 and 2.53 µM; respectively compared to doxorubicin (IC_50_ = 1.22 µM). Additionally, Compounds **4a, 4b, 4e, 4i,** and **4j** exhibited much lower toxicity than that of doxorubicin against normal Human Embryonic Kidney (HEK) 293 cells after 72 h incubation period. Also, hybrids **4a, 4b, 4e, 4i**, and **4j** exhibited remarkable inhibitory activities against Topo-I, Top-II, and tubulin polymerisation compared with the used references. They increased the protein expression level of γH2AX indicating DNA damage (double strand breaks). Furthermore, they arrested the HCT116 cell cycle at the G2/M phase possibly through ATR/CHK1/Cdc25C pathways as a result of DNA damage. Thus, the new CP hybrids have the capacity to be used as promising leads and advanced for further investigation as novel multi-target anticancer agents to combat colorectal carcinoma.

## 4. Experimental section

### 4.1. Chemistry

Thin-layer chromatography (Merck Grade-9385 precoated aluminium TLC plate silica gel 60, size 5 × 20 cm with 0.2 mm thickness) was used to monitor the chemical reaction. Plates were exposed to UV light at a wavelength of 254 nm to detect the spots. In addition, Stuart Electrothermal Melting Point Apparatus was used to determine the melting points (values were un-corrected). Also, 100 MHz for ^13 ^C and 400 MHz for ^1^H NMR spectra were obtained on Bruker AM400 spectrometer using DMSO-*d*_6_ (solvent) and tetramethylsilane (internal standard). In this work, δ (chemical shifts) and *J* (coupling constants) were reported in ppm and Hz, respectively. The following abbreviations were used to describe the NMR peak multiplicity: singlet = s, doublet = d, doublet of doublets = dd, triplet = t, quartette = q, multiplet = m, broad signal, brs. Shimadzu’s GC/MS-QP5050A (Regional Centre for Mycology and Biotechnology, Al-Azhar University, Cairo, Egypt) was used for elemental analyses while Advion’s compact mass spectrometer (Nawah Scientific Centre for Research, Almokattam, Cairo, Egypt) was used to obtain mass spectra (m/z).

### 4.1.1. Synthesis of the intermediates; 1, 2a-j, and 3

The intermediate 1^65^^,^^66^, **2a-j**^65^^,^^66^^,^^70^**, 3**^67^ were prepared according to the reported methods in the previous studies.

#### 4.1.2. Synthesis of compounds 4a-j

Our target compounds **4a-j** were synthesised by the reaction between Ciprofloxacin alkyne derivative **3** (0.5 g, 1.35 mmol.) and the appropriate azide **2a-j** (0.37 g, 1.48 mmol.) under the catalysis of sodium ascorbate (0.4 eq) and copper (II) sulphate pentahydrate (0.2 eq) as reported procedure^71^. See Supplementary Appendix A

##### 4.1.2.1. (*E*) 7-(4-((1–(4-Cinnamoylphenyl)-1*H*-1,2,3-triazol-4-yl)methyl)piperazin-1-yl)-1-cyclopropyl-6-fluoro-4-oxo-1,4-dihydroquinoline-3-carboxylic acid 4a

Yellowish white powder; (0.53 g, 63.85% yield); mp: 240–242 °C; IR (KBr) ν_max_ = broad band 3223-3074 cm^−1^ (OH of COOH), 1715 cm^−1^ (C = O of COOH), 1657 cm^−1^ (C = O of CH = CH–C = O) and 1622 (C = O of 4-keto gp of CP); ^1^H NMR (400 MHz, DMSO-d_6_) *δ* ppm: 1.16–1.22 (2H, m, cyclopropyl-*H*), 1.28–1.36 (2H, m, cyclopropyl-*H*), 2.70-2.80 (4H, m, piperazinyl-*H*), 3.36-3.40 (4H, m, piperazinyl-*H*), 3.77–3.85 (3H, m, *N*–C*H*_2_ and cyclopropyl-1*H*), 7.45–7.60 (4H, m, C8-*H* and Ar-*H*), 7.79 (1H, d, *J*_trans_ = 16 Hz, =C*H*), 7.83 (1H, d, *J*_HF_*_ortho_* = 16 Hz, C5-*H*), 7.90–7.92 (2H, m, Ar-*H*), 7.99 (1H, d, *J*_trans_ = 16 Hz, =C*H*), 8.15 (2H, d, *J* = 8 Hz, Ar-*H*), 8.35 (2H, d, *J* = 8 Hz, Ar-*H*), 8.62 (1H, s, C2-*H*), 8.93 (1H, s, triazole *H*), 15.14 (1H, brs, COO*H*); ^13 ^C NMR (100 MHz, CDCl_3_) *δ* ppm: 8.21, 35.26, 49.51, 51.42, 52.48, 52.90, 104.91, 108.23, 112.48 (C-5, d, *^2^J*_CF_*_ortho_* = 23 Hz, C-F), 120.12, 121.43, 126.64, 128.57, 129.06, 130.29, 130.91, 134.64, 138.18, 139.08, 140.92, 145.58, 145.81, 146.38, 147.42, 148.43, 153.66 (C-6, d, *^1^J*_CF_*_ipso_* = 250 Hz, C-F), 166.89, 177.09, 188.84; ESI-MS (*m/z*): Calcd. 618.24, found 641.60 [M + Na]^+^. Anal. Calcd. For C_35_H_31_FN_6_O_4_: C, 67.95%; H, 5.05%; N, 13.58%. Found: C, 67.84%; H, 5.21%; N, 13.74%.

##### 4.1.2.2. (*E*)-7-(4-((1–(4-(3-(3-chlorophenyl)acryloyl)phenyl)-1*H*-1,2,3-triazol-4-yl)methyl) piperazin-1-yl)-1-cyclopropyl-6-fluoro-4-oxo-1,4-dihydroquinoline-3-carboxylic acid 4b

Yellow powder; (0.59 g, 66.91% yield); mp: 254–247 °C; ^1^H NMR (400 MHz, DMSO-d_6_) *δ* ppm: 1.18–1.24 (2H, m, cyclopropyl-*H*), 1.31–1.35 (2H, m, cyclopropyl-*H*), 2.67–2.73 (4H, m, piperazinyl-*H*), 3.35–3.39 (4H, m, piperazinyl-*H*), 3.79–3.85 (3H, m, *N*–C*H*_2_ and cyclopropyl-1*H*), 7.53–7.60 (2H, m, Ar-*H* and C8-*H*), 7.77 (1H, d, *J*_trans_ = 16 Hz, =C*H*), 7.84–7.91 (2H, m, Ar-*H* and C5-*H*), 8.09-8.18 (3H, m, =C*H* and, Ar-*H*), 8.17 (2H, d, *J* = 8 Hz, Ar-*H*), 8.42 (2H, d, *J* = 8 Hz, Ar-*H*), 8.65 (1H, s, C2-*H*), 8.92 (1H, s, triazole *H*), 15.17 (1H, brs, COO*H*); ^13 ^C NMR (100 MHz, CDCl_3_) *δ* ppm: 7.22, 34.31, 48.34, 50.44, 51.43, 51.85, 103.99, 107.17, 111.43 (C-5, d, *^2^J*_CF_*_ortho_* = 23 Hz, C-F), 119.17, 121.59, 125.94, 127.03, 129.33, 129.70, 134.14, 135.49, 136.87, 138.08, 138.91, 139.33, 139.84, 142.98, 144.58, 144.76, 146.43, 147.55, 152.64 (C-6, d, *^1^J*_CF_*_ipso_* = 250 Hz, C-F), 165.88, 176.05, 187.40; ESI-MS (*m/z*): Calcd. 652.20, found 675.80 [M + Na]^+^. Anal. Calcd. For C_35_H_30_ClFN_6_O_4_: C, 64.37%; H, 4.63%; N, 12.87%. Found: C, 64.53%; H, 4.80%; N, 13.09%.

##### 4.1.2.3. (*E*)-7-(4-((1-(4-(3-(4-chlorophenyl)acryloyl)phenyl)-1*H*-1,2,3-triazol-4-yl)methyl) piperazin-1-yl)-1-cyclopropyl-6-fluoro-4-oxo-1,4-dihydroquinoline-3-carboxylic acid 4c

Yellow powder; (0.58 g, 65.89% yield); mp: 257–259 °C; ^1^H NMR (400 MHz, DMSO-d_6_) *δ* ppm: 1.17–1.22 (2H, m, cyclopropyl-*H*), 1.30–1.35 (2H, m, cyclopropyl-*H*), 2.70–2.76 (4H, m, piperazinyl-*H*), 3.36–3.40 (4H, m, piperazinyl-*H*), 3.80–3.82 (3H, m, *N*-C*H*_2_ and cyclopropyl-1*H*), 7.55 (2H, d, *J* = 8 Hz, Ar-*H*), 7.57(1H, d, *J*_HF_*_meta_* = 8 Hz, C8-*H*), 7.79 (1H, d, *J*_trans_ = 16 Hz, =C*H*), 7.90 (1H, d, *J*_HF_*_ortho_* = 12 Hz, C5-*H*), 7.97 (2H, d, *J* = 8 Hz, Ar-*H*), 8.03 (1H, d, *J*_trans_ = 16 Hz, =C*H*), 8.16 (2H, d, *J* = 8 Hz, Ar-*H*), 8.39 (2H, d, *J* = 8 Hz, Ar-*H*), 8.66 (1H, s, C2-*H*), 8.94 (1H, s, triazole *H*), 15.17 (1H, brs, COO*H*); ^13 ^C NMR (100 MHz, CDCl_3_) *δ* ppm: 8.20, 35.28, 49.44, 52.45, 52.88, 104.92, 108.18, 112.42 (C-5, d, *^2^J*_CF_*_ortho_* = 24 Hz, C-F), 120.12, 121.18, 121.79, 126.33, 129.36, 129.70, 130.27, 133.12, 136.88, 137.97, 139.06, 139.85, 144.22, 144.51, 145.59, 147.40, 153.62 (C-6, d, *^1^J*_CF_*_ipso_* = 252 Hz, C-F), 166.85, 177.04, 188.50; ESI-MS (*m/z*): Calcd. 652.20, found 675.80 [M + Na]^+^. Anal. Calcd. For C_35_H_30_ClFN_6_O_4_: C, 64.37%; H, 4.63%; N, 12.87%. Found: C, 64.51%; H, 4.89%; N, 13.04%.

##### 4.1.2.4. (*E*)-7-(4-((1-(4-(3-(4-bromophenyl)acryloyl)phenyl)-1*H*-1,2,3-triazol-4-yl) methyl)piperazin-1-yl)-1-cyclopropyl-6-fluoro-4-oxo-1,4-dihydroquinoline-3-carboxylic acid 4d

Pale yellow powder; (0.57 g, 60.50% yield); mp: 247–249 °C; ^1^H NMR (400 MHz, DMSO-d_6_) *δ* ppm: 1.17–1.21 (2H, m, cyclopropyl-*H*), 1.31–1.35 (2H, m, cyclopropyl-*H*), 2.69–2.75 (4H, m, piperazinyl-*H*), 3.38–3.43 (4H, m, piperazinyl-*H*), 3.81–3.83 (3H, m, *N*-C*H*_2_ and cyclopropyl-1*H*), 7.57(1H, d, *J*_HF_*_meta_* = 8 Hz, C8-*H*), 7.68 (2H, d, *J* = 8 Hz, Ar-H), 7.76 (1H, d, *J*_tran_*_s_* = 16 Hz, =C*H*), 7.88 (2H, d, *J* = 8 Hz, Ar-*H*), 7.91 (1H, d, *J*_HF_*_ortho_* = 12 Hz, C5-*H*), 8.02 (1H, d, *J*_tran_*_s_* = 16 Hz, =C*H*), 8.15 (2H, d, *J* = 8 Hz, Ar-*H*), 8.38 (2H, d, *J* = 8 Hz, Ar-*H*), 8.66 (1H, s, C2-*H*), 8.91 (1H, s, triazole *H*), 15.05 (1H, brs, COO*H*); ^13 ^C NMR (100 MHz, CDCl_3_) *δ* ppm: 8.22, 35.30, 49.62, 51.41, 52.22, 104.86, 105.18, 108.18, 112.42 (C-5, d, *^2^J*_CF_*_ortho_* = 23 Hz, C-F), 120.22, 121.89, 125.24, 129.90, 130.70, 131.08, 131.54, 132.33, 133.55, 138.06, 139.05, 139.74, 144.31, 145.15, 145.81, 153.61 (C-6, d, *^1^J*_CF_*_ipso_* = 260 Hz, C-F), 166.90, 177.02, 188.50; ESI-MS (*m/*z): Calcd. 696.15, found 719.50 [M + Na]^+^. Anal. Calcd. For C_35_H_30_BrFN_6_O_4_: C, 60.26%; H, 4.34%; N, 12.05%. Found: C, 60.53%; H, 4.52%; N, 12.31%.

##### 4.1.2.5. (*E*)-1-cyclopropyl-6-fluoro-7–(4-((1–(4-(3-(4-fluorophenyl)acryloyl) phenyl)-1*H*-1,2,3-triazol-4-yl)methyl)piperazin-1-yl)-4-oxo-1,4-dihydroquinoline-3-carboxylic acid 4e

Yellow powder; (0.40 g, 46.56% yield); mp: 203–205 °C; IR (KBr) ν_max_= broad band 3283–3066 cm^−1^ (OH of COOH), 1718 cm^−1^ (C = O of COOH), 1658 cm^−1^ (C = O of CH = CH–C = O) and 1624 (C = O of 4-keto gp of CP); ^1^H NMR (400 MHz, DMSO-d_6_) *δ* ppm: 1.17–1.21 (2H, m, cyclopropyl-*H*), 1.31–1.34 (2H, m, cyclopropyl-*H*), 2.75–2.80 (4H, m, piperazinyl-*H*), 3.39–3.45 (4H, m, piperazinyl-*H*), 3.82–3.87 (3H, m, *N*-C*H*_2_ and cyclopropyl-1*H*), 7.30 (2H, t, *J*_HF_ = 8 Hz, Ar-*H*), 7.57(1H, d, *J*_HF_*_meta_* = 8 Hz, C8-*H*), 7.79 (1H, d, *J*_trans_ = 16 Hz, =C*H*), 7.86–7.93 (2H, m, =C*H* and C5-*H*), 7.97 (2H, d, *J* = 8 Hz, Ar-*H*), 8.13 (2H, d, *J* = 8 Hz, Ar-*H*), 8.35 (2H, d, *J* = 8 Hz, Ar-*H*), 8.66 (1H, s, C2-*H*), 8.86 (1H, s, triazole *H*), 15.05 (1H, brs, COO*H*); ^13 ^C NMR (100 MHz, CDCl_3_) *δ* ppm: 6.41, 33.50, 47.43, 50.58, 50.98, 103.22, 106.42, 110.64 (C-5, d, *^2^J*_CF_*_ortho_* = 23 Hz, C-F), 113.67, 114.46 (C_Ar_, d, *^2^J*_CF_*_ortho_* = 23 Hz, C-F), 118.35, 119.38, 119.74, 128.43, 128.67, 128.75, 129.14, 136.36, 137.27, 137.97, 142.62, 143.64, 145.62, 151.82 (C-6, d, *^1^J*_CF_*_ipso_* = 251 Hz, C-F), 162.53 (C_Ar_, d, *^1^J*_CF_*_ipso_* = 252 Hz, C-F), 165.01, 175.23, 186.77; ESI-MS (*m/z*): Calcd. 636.23, found 659.03 [M + Na]^+^. Anal. Calcd. For C_35_H_30_F_2_N_6_O_4_: C, 66.03%; H, 4.75%; N, 13.20%. Found: C, 65.94%; H, 4.89%; N, 12.98%.

##### 4.1.2.6. (*E*)-1-cyclopropyl-6-fluoro-4-oxo-7-(4-((1-(4-(3-(*p*-tolyl)acryloyl)phenyl)-1*H*-1,2,3-triazol-4-yl)methyl)piperazin-1-yl)-1,4-dihydroquinoline-3-carboxylic acid 4f

Yellow white powder; (0.47 g, 55.03% yield); mp: 259–261 °C; ^1^H NMR (400 MHz, DMSO-d_6_) *δ* ppm: 1.15–1.23 (2H, m, cyclopropyl-*H*), 1.29–1.34 (2H, m, cyclopropyl-*H*), 2.37 (3H, s, Ar-C*H*_3_), 2.71–2.75 (4H, m, piperazinyl-*H*), 3.39-3.46 (4H, m, piperazinyl-*H*), 3.79–3.83 (3H, m, *N*-C*H*_2_ and cyclopropyl-1*H*), 7.30 (2H, d, *J* = 8 Hz, Ar-*H*), 7.56 (1H, d, *J*_HF_*_meta_* = 8 Hz, C8-*H*), 7.75–7.82 (3H, m, =C*H* and Ar-*H*), 7.89–7.99 (2H, m, C5-*H* and = C*H*), 8.14 (2H, d, *J* = 8 Hz, Ar-*H*), 8.35 (2H, d, *J* = 8 Hz, Ar-*H)*, 8.66 (1H, s, C2-*H*), 8.96 (1H, s, triazole *H*), 15.05 (1H, brs, COO*H*); ^13 ^C NMR (100 MHz, CDCl_3_) *δ* ppm: 8.20, 21.50, 35.26, 49.38, 50.46, 52.41, 52.83, 104.96, 108.27, 112.48 (C-5, d, *^2^J*_CF_*_ortho_* = 23 Hz, C-F), 120.11, 120.47, 124.77, 128.60, 129.03, 129.79, 130.21, 131.94, 138.41, 139.07, 139.70, 141.55, 145.48, 145.89, 147.41, 148.32, 153.64 (C-6, d, *^1^J*_CF_*_ipso_* = 252 Hz, C-F), 166.79, 177.06, 188.90; ESI-MS (*m/z*): Calcd. 632.25, found 655.40 [M + Na]^+^. Anal. Calcd. For C_36_H_33_FN_6_O_4_: C, 68.34%; H, 5.26%; N, 13.28%. Found: C, 68.35%; H, 5.40%; N, 13.41%.

##### 4.1.2.7. (*E*)-1-Cyclopropyl-6-fluoro-7-(4-((1-(4-(3-(4-methoxyphenyl)acryloyl) phenyl)-1*H*-1,2,3-triazol-4-yl)methyl)piperazin-1-yl)-4-oxo-1,4-dihydroquinoline-3-carboxylic acid 4 g

Yellow powder; (0.47 g, 53.67% yield); mp: 254–257 °C; ^1^H NMR (400 MHz, DMSO-d_6_) *δ* ppm: 1.18–1.24 (2H, m, cyclopropyl-*H*), 1.29–1.34 (2H, m, cyclopropyl-*H*), 2.70–2.75 (4H, m, piperazinyl-*H*), 3.35–3.40 (4H, m, piperazinyl-*H*), 3.76 (3H, s, OC*H*_3_), 3.81–3.85 (3H, m, N-C*H*_2_, cyclopropyl-*H*), 7.04 (2H, d, *J* = 8 Hz, Ar-*H*), 7.56 (1H, d, *J*_HF_*_meta_* = 8 Hz, C8-*H*), 7.77 (1H, d, *J_trans_* = 16 Hz, =C*H*), 7.85–7.90 (4H, m, =C*H*, Ar-*H* and C5*-H*), 8.14 (2H, d, *J* = 8 Hz, Ar-*H*), 8.36 (2H, d, *J* = 8 Hz, Ar-*H*), 8.66 (1H, s, C2-*H*), 8.93 (1H, s, triazole-*H*), 15.16 (1H, brs, COO*H*); ^13 ^C NMR (100 MHz, CDCl_3_) *δ* ppm: 8.21, 35.25, 49.45, 52.47, 52.90, 55.45, 104.91, 108.30, 112.56 (C-5, d, *^2^J*_CF_*_ortho_* = 23 Hz, C-F), 113.73, 114.58, 119.07, 120.07, 122.97, 127.38, 130.17, 130.42, 131.87, 138.58, 139.07, 139.58, 141.40, 145.69, 147.45, 153.66 (C-6, d, *^1^J*_CF_*_ipso_* = 252 Hz, C-F), 162.09, 166.88, 177.12, 188.83; ESI-MS (*m/z*): Calcd. 648.25, found 671.60 [M + Na]^+^. Anal. Calcd. For C_36_H_33_FN_6_O_5_: C, 66.66%; H, 5.13%; N, 12.96%. Found: C, 66.39%; H, 5.36%; N, 13.18%.

##### 4.1.2.8. (*E*)-1-cyclopropyl-7–(4-((1–(4-(3-(4-(dimethylamino)phenyl)acryloyl) phenyl)-1*H*-1,2,3-triazol-4-yl)methyl)piperazin-1-yl)-6-fluoro-4-oxo-1,4-dihydroquinoline-3-carboxylic acid 4h

Pale yellow powder; (0.39 g, 43.65% yield); mp: 177–179 °C; ^1^H NMR (400 MHz, DMSO-d_6_) *δ* ppm: 1.17–1.21 (2H, m, cyclopropyl-*H*), 1.31–1.35 (2H, m, cyclopropyl-*H*), 2.74–2.78 (4H, m, piperazinyl-*H*), 3.04 (6H, s, N(C*H*_3_)_2_), 3.39–3.45 (4H, m, piperazinyl-*H*), 3.82–3.87 (3H, m, *N*–C*H*_2_ and cyclopropyl-1*H*), 6.79 (2H, d, *J* = 8 Hz, Ar-*H*), 7.58–7.65 (2H, m, C8-*H* and = C*H*), 7.70–7.76 (3H, m, Ar-*H* and = C*H*), 7.91 (1H, d, *J*_HF_*_ortho_* = 12 Hz, C5-*H*), 8.10 (2H, d, *J* = 8 Hz, Ar-*H*), 8.30 (2H, d, *J* = 8 Hz, Ar-*H*), 8.67 (1H, s, C2-*H*), 8.83 (1H, s, triazole *H*), 15.07 (1H, brs, COO*H*); ^13 ^C NMR (100 MHz, CDCl_3_) *δ* ppm: 8.19, 35.28, 40.06, 49.57, 52.49, 52.97, 104.89, 108.15, 111.86, 112.38 (C-5, d, *^2^J*_CF_*_ortho_* = 23 Hz, C-F), 116.02, 119.82, 119.97, 121.04, 122.34, 130.00, 130.65, 139.07, 139.13, 139.31, 144.77, 145.63, 145.72, 146.79, 147.37, 153.63 (C-6, d, *^1^J*_CF_*_ipso_* = 252 Hz, C-F), 166.91, 177.06, 188.79; ESI-MS (*m/z*): Calcd. 661.28, found 684.50 [M + Na]^+^. Anal. Calcd. For C_37_H_36_FN_7_O_4_: C, 67.16%; H, 5.48%; N, 14.82%. Found: C, 67.43%; H, 5.41%; N, 14.70%.

##### 4.1.2.9. (*E*)-1-cyclopropyl-7-(4-((1-(4-(3–(3,4-dimethoxyphenyl)acryloyl) phenyl)-1*H*-1,2,3-triazol-4-yl)methyl)piperazin-1-yl)-6-fluoro-4-oxo-1,4-dihydroquinoline-3-carboxylic acid 4i

Yellow powder; (0.52 g, 56.77% yield); mp: 167–169 °C; IR (KBr) ν_max_= broad band 3234–3067 cm^−1^ (OH of COOH), 1719 cm^−1^ (C = O of COOH), 1658 cm^−1^ (C = O of CH = CH–C = O) and 1625 (C = O of 4-keto gp of CP); ^1^H NMR (400 MHz, DMSO-d_6_) *δ* ppm: 1.18–1.22 (2H, m, cyclopropyl-*H*), 1.29–1.32 (2H, m, cyclopropyl-*H*), 2.70-2.75 (4H, m, piperazinyl-*H*), 3.35–3.42 (4H, m, piperazinyl-*H*), 3.81–3.84 (6H, m, N-C*H*_2_, cyclopropyl-*H,* OC*H*_3_), 3.88 (3H, s, OC*H*_3_), 7.04–7.08 (1H, m, Ar-*H*), 7.43-7.45 (1H, m, Ar-*H*), 7.55–7.60 (2H, m, Ar-*H* and C8*-H*), 7.76 (1H, d, *J_trans_* = 16 Hz, =C*H*), 7.86–7.93 (2H, m, C5-*H* and = C*H*), 8.15 (2H, d, *J* = 8 Hz, Ar-*H*), 8.37 (2H, d, *J* = 8 Hz, Ar-*H*), 8.66 (1H, s, C2-*H*), 8.94 (1H, s, triazole-*H*), 15.17 (1H, brs, COO*H*); ^13 ^C-NMR (100 MHz, CDCl_3_) *δ* ppm: 8.20, 35.29, 49.48, 52.45, 52.89, 55.89, 56.06, 104.92, 108.13, 110.48, 111.32, 112.36 (C-5, d, *^2^J*_CF_*_ortho_* = 23 Hz, C-F), 119.34, 120.07, 121.18, 123.45, 127.62, 130.19, 138.45, 139.06, 139.63, 145.58, 145.98, 147.38, 148.45, 149.44, 151.78, 151.91, 153.62 (C-6, d, *^1^J*_CF_*_ipso_* = 252 Hz, C-F), 166.87, 177.02, 188.87; ESI-MS (m/z): Calcd. 678.26, found 701.50 [M + Na]^+^. Anal. Calcd. For C_37_H_35_FN_6_O_6_: C, 65.48%; H, 5.20%; N, 12.38%. Found: C, 65.69%; H, 5.37%; N, 12.54%.

##### 4.1.2.10. (*E*)-1-cyclopropyl-6-fluoro-4-oxo-7–(4-((1–(4-(3-(3,4,5-trimethoxyphenyl) acryloyl)phenyl)-1*H*-1,2,3-triazol-4-yl)methyl)piperazin-1-yl)-1,4-dihydroquinoline-3-carboxylic acid 4j

Yellow powder; (0.49 g, 51.21% yield); mp: 247–249 °C; ^1^H NMR (400 MHz, DMSO-d_6_) *δ* ppm: 1.18–1.22 (2H, m, cyclopropyl-*H*), 1.27–1.32 (2H, m, cyclopropyl-*H*), 2.69–2.76 (4H, m, piperazinyl-*H*), 3.35–3.45 (4H, m, piperazinyl-*H*), 3.74 (3H, s, OC*H*_3_), 3.78–3.85 (3H, m, N-C*H*_2_, cyclopropyl-*H*), 3.89 (6H, s, 2X OC*H*_3_), 7.26 (2H, s, Ar-*H*), 7.57 (1H, d, *J*_HF_*_meta_* = 8 Hz, C8-*H*), 7.75 (1H, d, *J_trans_* = 16 Hz, =C*H*), 7.87 (1H, d, *J*_HF_*_ortho_* = 12 Hz, C5-*H*), 7.94 (1H, d, *J_trans_* = 16 Hz, =C*H*), 8.15 (2H, d, *J* = 8 Hz, Ar-*H*), 8.37 (2H, d, *J* = 8 Hz, Ar-*H*), 8.65 (1H, s, C2-*H*), 8.94 (1H, s, triazole-*H*), 15.16 (1H, brs, COO*H*); ^13 ^C NMR (100 MHz, CDCl_3_) δ ppm: 8.22, 35.27, 49.15, 49.44, 52.42, 56.35, 61.00, 104.95, 106.11, 107.52, 108.23, 112.46 (C-5, d, *^2^J*_CF_*_ortho_* = 23 Hz, C-F), 120.14, 120.76, 121.29, 130.02, 130.30, 137.38, 138.28, 139.07, 140.51, 141.07, 144.76, 146.00, 147.42, 153.21 (C-6, d, *^1^J*_CF_*_ipso_* = 252 Hz, C-F), 153.62, 166.85, 177.07, 188.87; ESI-MS (*m/z*): Calcd. 707.28, found 730.26 [M + Na]^+^. Anal. Calcd. For C_38_H_37_FN_6_O_7_: C, 64.40%; H, 5.26%; N, 11.86%. Found: C, 64.53%; H, 5.49%; N, 12.03%. HPLC analysis: 94.39%.

### 4.2. Biological investigations

#### 4.2.1. Cell culture experiments and reagents

Colo-rectal cancer cell lines (Caco-2, HT29, and HCT116) were purchased from the American Type Culture Collection (Manassas, VA, USA). Cells were cultured and maintained in appropriate media supplemented with foetal bovine serum (10%) (Sigma-Aldrich, St. Louis, MO, USA), streptomycin (100 μg/mL), and penicillin (100 U/mL) (Life Technologies) and incubated at 37 °C with CO_2_ (5%). Ciprofloxacin derivatives were chemically synthesised (**4a-j**) while doxorubicin was obtained from Sigma-Aldrich. Analytical or cell culture grade chemicals were used throughout this study.

#### 4.2.2. Proliferation assay

The method description for NCI anticancer screening is provided in detail in the standard NCI-60 testing protocol^72^.

#### 4.2.3. 3–(4,5-dimethylthiazol-2-yl)-2,5-diphenyltetrazolium bromide (MTT) assay

The proliferation of colorectal cancer cells (HCT116, HT29 and Caco-2) was assessed using MTT assay as previously reported^73^. Cells were treated with increasing concentrations of 1, 5, 10, 20, 40, 80 or 100 µM of CP hybrids **4a-j**, doxorubicin, or DMSO. See Supplementary Appendix A.

#### 4.2.4. Cytotoxicity assay with non-cancerous cells

To evaluate the selectivity and safety profile of the synthesised ciprofloxacin-chalcone hybrids, a cytotoxicity experiment was performed using a non-cancerous cell line. Briefly, Human Embryonic Kidney (HEK) 293 cells (ATCC, Manassas, VA, USA) were cultured in a Dulbecco’s Modified Eagle Medium (DEMEM) supplemented with 1 mM sodium pyruvate, 10 mM HEPES buffer solution, 2 mM GlutaMAX (Gibco, Grand Island, NY, USA), 50 µg/mL gentamycin sulphate (Dubuque, IA, USA), and 10% foetal bovine serum (ThermoFisher Scientific, USA). Cells were seeded in 96-well plates and incubated overnight in a humidified atmosphere with 5% CO_2_ at 37 °C. After 24 h, cells were treated with different concentrations (0.01–100 µM) of ciprofloxacin-chalcone hybrids. Also, DMSO and doxorubicin were used as negative and positive controls, respectively. After 72 h, old medium containing treatments were removed, and fresh medium and MTS reagents (CellTiter 96® AQueous One Solution Cell Proliferation Assay, Promega, Madison, WI, USA) were added. The plates were then incubated at 37 °C. After 2 h, the absorbance was measured at 490 nm using a Spectra Max plus 384 Microplate reader (Molecular Devices, Sunnyvale, CA, USA) ^74^. Also, before adding the MTS reagents, cells were examined under a cell imaging system (EVOS FL Digital Microscope using a 20x objective lens, total magnification = 200×).

#### 4.2.5. Topoisomerase I inhibition assay

Two different assays were used to examine the inhibitory activity of IC_50_ of the CP hybrids against Human topoisomerase I. Firstly; Gel electrophoresis-based assay (DNA relaxation assay) was performed following the manufacturer's protocol (TG-1015, Topogen Inc., USA). Secondly, the inhibitory activity of compounds **4a, 4b, 4e, 4i, 4j**, and camptothecin to human topoisomerase I was analysed by Human DNA Topoisomerase I Assay Kit (ProFoldin, Hudson, MA, USA) with recombinant human DNA topoisomerase I (Sigma-Aldrich) according to the manufacturer’s protocol.

#### 4.2.6. Topoisomerase II inhibition assay

The inhibitory activity of the selected ciprofloxacin hybrids on human Top-II was tested using a Human Topoisomerase II assay kit (TG1001, Topogen Inc.). In this assay, 1% agarose gel was used, and the compounds were tested at two different concentrations, 10 and 100 µM. Also, etoposide was used as a positive control in this experiment. The assay was performed according to the manufacturer’s protocol. The image of the electrophoretic gel was captured using an imaging system (iBright-CL1500, Thermo Fisher Scientific).

#### 4.2.7. Immunofluorescence microscopy

HCT116 cells were cultured and treated with the IC_50_ of the potent compounds (**4a, 4b, 4e, 4i**, and **4j**) or DMSO (control), fixed, permeabilized, and they were incubated with either γH2AX or α-tubulin antibody as the reported procedure in the literature^75^. Confocal images were captured using a Confocal Microscope with a 63X objective. All images were processed using ImageJ software. See Supplementary Appendix A.

#### 4.2.8. Analysis of cell cycle distribution

The effects of the most potent CP hybrids; **4a, 4b, 4e, 4i**, and **4j,** and assay controls**;** Doxorubicin, camptothecin, and etoposide on cell cycle progression were analysed through propidium iodide staining and subsequent flow cytometry according to the reported procedure^76^. See Supplementary Appendix A.

#### 4.2.9. Western blotting

Western blotting analysis for HCT116 cells was performed according to the reported procedure in the literature^77–79^ to determine the effects of CP hybrids; **4a, 4b, 4e, 4i**, and **4j** on the expression of polymerised tubulin, γH2AX, p-ATR, p-CHK1, and p-Cdc25C by using the appropriate antibody. See Supplementary Appendix A.

### 4.3. Docking studies

Compounds **4a, 4b, 4e, 4i, and 4j** were drawn and docked in the human Topo-I in complex with DNA and camptothecin (PDB ID: 1T8I) and in the human Top II in complex with DNA and Etoposide (PDB ID: **6ZY7**) using Molecular Operating Environment (MOE 2019.01) program as reported in the literature. See Supplementary Appendix A.

## Supplementary Material

Supplemental Material
