# Supplementary material for: New 1,2,3-triazole linked ciprofloxacin-chalcones induce DNA damage by inhibiting human topoisomerase I& II and tubulin polymerization
Source: J Enzyme Inhib Med Chem. 2022 May 11;37(1):1346–63. doi: 10.1080/14756366.2022.2072308 (PMC9116245; doi:10.1080/14756366.2022.2072308)
Supplement: Supplemental Material [file IENZ_A_2072308_SM9571.pdf]

New 1,2,3-Triazole Linked Ciprofloxacin-Chalcones induce DNA Damage by Inhibiting Human Topoisomerase I& II and Tubulin Polymerization.

Hamada H.H. Mohammed#[1,2], Amer Ali Abd El-Hafeez #[3,4], Kareem Ebeid[5,6,7], Aml I. Mekkawy[7,8], Mohammed A.S. Abourehab[9], Emad I. Wafa[7], Suhaila O. Alhaj-Suliman[7], Aliasger K. Salem[*7,10], Pradipta Ghosh[4,11,12,13], Gamal El-Din A Abuo-Rahma*[1,14], Alaa M. Hayallah[15,16], Samar H Abbas*[1].

[1]Department of Medicinal Chemistry, Faculty of Pharmacy, Minia University, Minia 61519, Egypt.

[2]Department of Pharmaceutical Chemistry, Faculty of Pharmacy, Sohag University, Sohag 82524, Egypt

[3]Pharmacology and Experimental Oncology Unit, Cancer Biology Department, National Cancer Institute, Cairo University, Cairo, 11796, Egypt

[4]Department of Cellular and Molecular Medicine, University of California San Diego, La Jolla, CA, USA

[5]Department of Pharmaceutics, Faculty of Pharmacy, Minia University, Minia, Minia 61519, Egypt

[6]Department of Pharmaceutics, Faculty of Pharmacy and Pharmaceutical Manufacturing, Deraya University, New Minia City, Minia 61768, Egypt

[7]Department of Pharmaceutical Sciences and Experimental Therapeutics, College of Pharmacy, University of Iowa, Iowa City, IA 52242, USA

[8]Department of Pharmaceutics and Clinical Pharmacy, Faculty of Pharmacy, Sohag University, Sohag, Sohag 82524, Egypt

[9]Department of Pharmaceutics, Faculty of Pharmacy Umm Al-Qura University, Makkah 21955 Saudi Arabia, maabourehab@uqu.edu.sa

[10]Holden Comprehensive Cancer Center, University of Iowa, Iowa City, IA 52242, USA

[11]Department of Medicine, University of California San Diego, La Jolla, CA, USA

[12]Rebecca and John Moore Comprehensive Cancer Center, University of California San Diego, La Jolla, CA, USA

[13]Veterans Affairs Medical Center, La Jolla, CA, USA

[14]Department of Pharmaceutical Chemistry, Faculty of Pharmacy, Deraya University, Minia, New Minia 61519, Egypt.

[15]Pharmaceutical Organic Chemistry Department, Faculty of Pharmacy, Assiut University, 71526, Egypt

[16]Pharmaceutical Chemistry Department, Faculty of Pharmacy, Sphinx University, New Assiut, Egypt

**To whom correspondence should be addressed:*

*Samar H Abbas: samar_hafez@mu.edu.eg , +201005424005

*G. E-D. A. Abuo -Rahma: gamal.aborahama@mu.edu.eg, +201003069431

*Aliasger K. Salem: aliasger-salem@uiowa.edu

# These authors contributed equally to this work.

Novel Multi-Targeting Anti-proliferative 1,2,3-Triazole Linked Ciprofloxacin-Chalcones Display DNA Damage by Inhibiting Human Topoisomerase I, Topoisomerase II, and Tubulin Polymerization.

Hamada H.H. Mohammed#[1,2,3], Amer Ali Abd El-Hafeez #[4,5], Kareem Ebeid[6,7,8], Aml I. Mekkawy[8,9], Emad I. Wafa[8], Suhaila O. Alhaj-Suliman[8], Aliasger K. Salem[*8,10], Pradipta Ghosh[5,11,12,13], Gamal El-Din A Abuo-Rahma*[1,2], Alaa M. Hayallah[14,15], Samar H Abbas*[1].

[1]Department of Medicinal Chemistry, Faculty of Pharmacy, Minia University, Minia 61519, Egypt.

[2]Department of Pharmaceutical Chemistry, Faculty of Pharmacy, Deraya University, Minia, New Minia 61519, Egypt.

[3]Department of Pharmaceutical Chemistry, Faculty of Pharmacy, Merit University, Sohag, New Sohag 82524, Egypt.

[4]Pharmacology and Experimental Oncology Unit, Cancer Biology Department, National Cancer Institute, Cairo University, Cairo, 11796, Egypt

[5]Department of Cellular and Molecular Medicine, University of California San Diego, La Jolla, CA, USA

[6]Department of Pharmaceutics, Faculty of Pharmacy, Minia University, Minia, Minia 61519, Egypt

[7]Department of Pharmaceutics, Faculty of Pharmacy and Pharmaceutical Manufacturing, Deraya University, New Minia City, Minia 61768, Egypt

[8]Department of Pharmaceutical Sciences and Experimental Therapeutics, College of Pharmacy, University of Iowa, Iowa City, IA 52242, USA

[9]Department of Pharmaceutics and Clinical Pharmacy, Faculty of Pharmacy, Sohag University, Sohag, Sohag 82524, Egypt

[10]Holden Comprehensive Cancer Center, University of Iowa, Iowa City, IA 52242, USA

[11]Department of Medicine, University of California San Diego, La Jolla, CA, USA

[12]Rebecca and John Moore Comprehensive Cancer Center, University of California San Diego, La Jolla, CA, USA

[13]Veterans Affairs Medical Center, La Jolla, CA, USA

[14]Pharmaceutical Organic Chemistry Department, Faculty of Pharmacy, Assiut University, 71526, Egypt

[15]Pharmaceutical Chemistry Department, Faculty of Pharmacy, Sphinx University, New Assiut, Egypt

**To whom correspondence should be addressed:*

*Samar H Abbas: samar_hafez@mu.edu.eg , +201005424005

*G. E-D. A. Abuo -Rahma: gamal.aborahama@mu.edu.eg, +201003069431

*Aliasger K. Salem: aliasger-salem@uiowa.edu

# These authors contributed equally to this work

1 H NMR spectrum of compound 4a

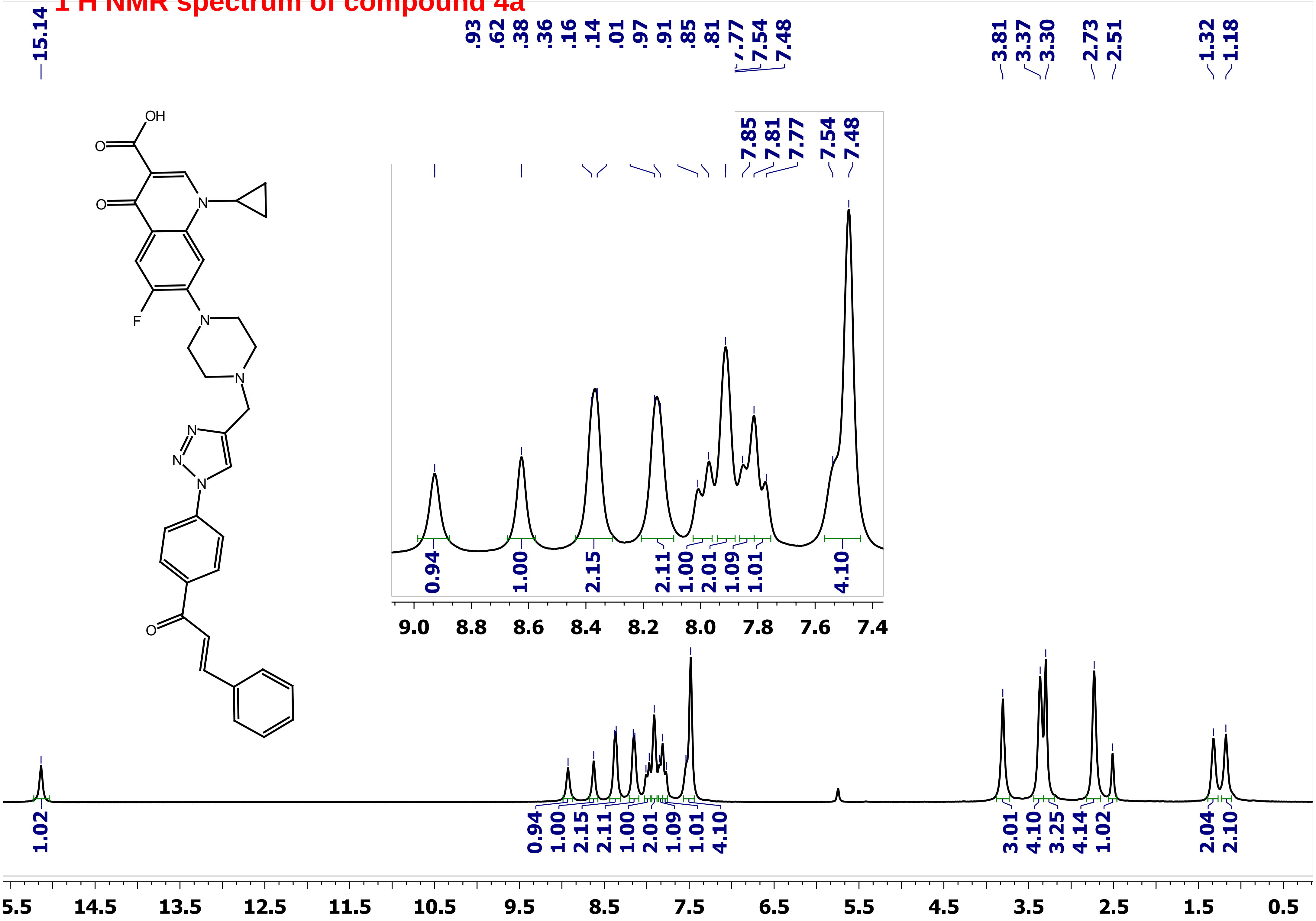

13 C NMR spectrum of compound 4a

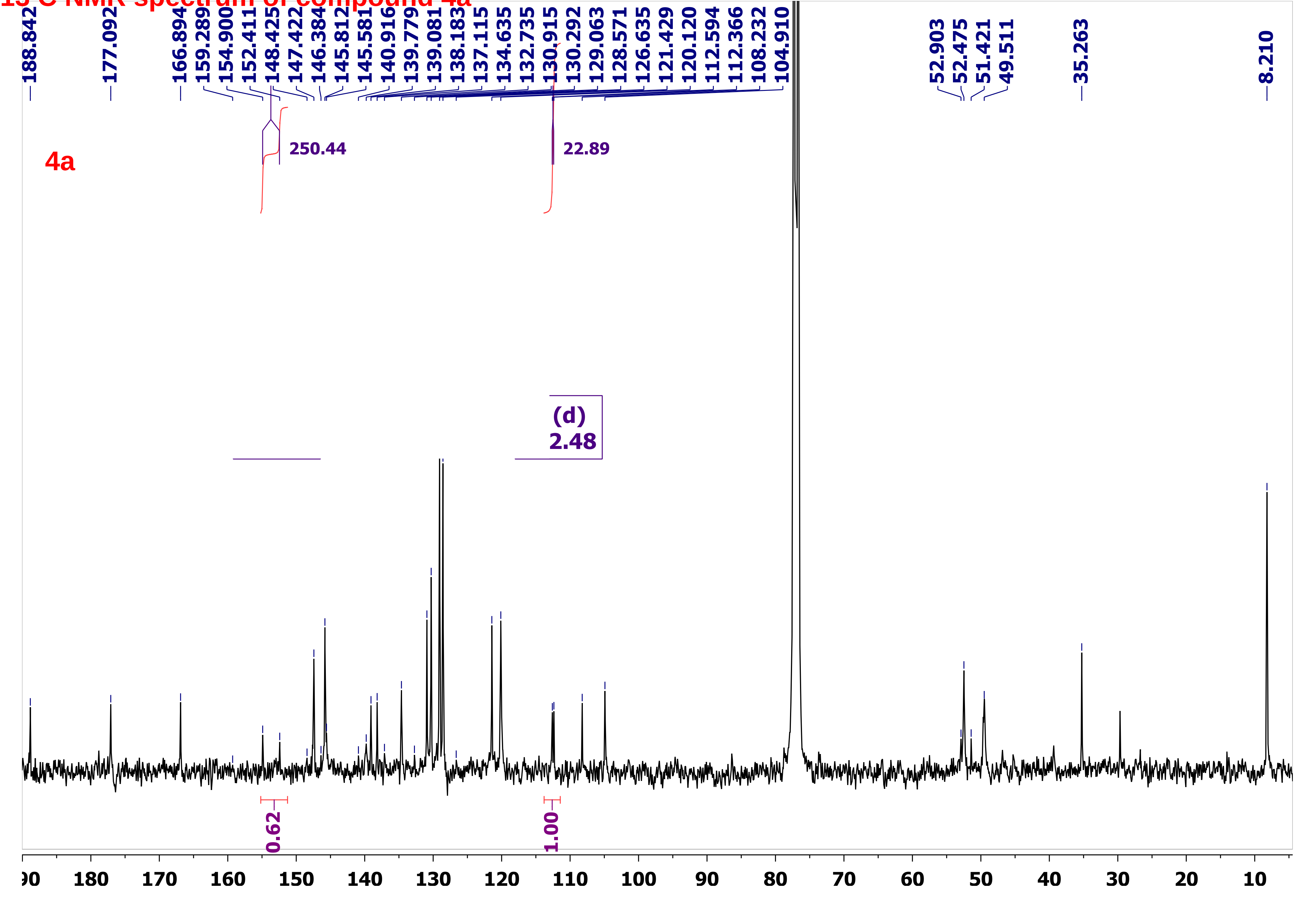

Mass (ESI) spectrum of compound 4a [M+Na] +.

4a

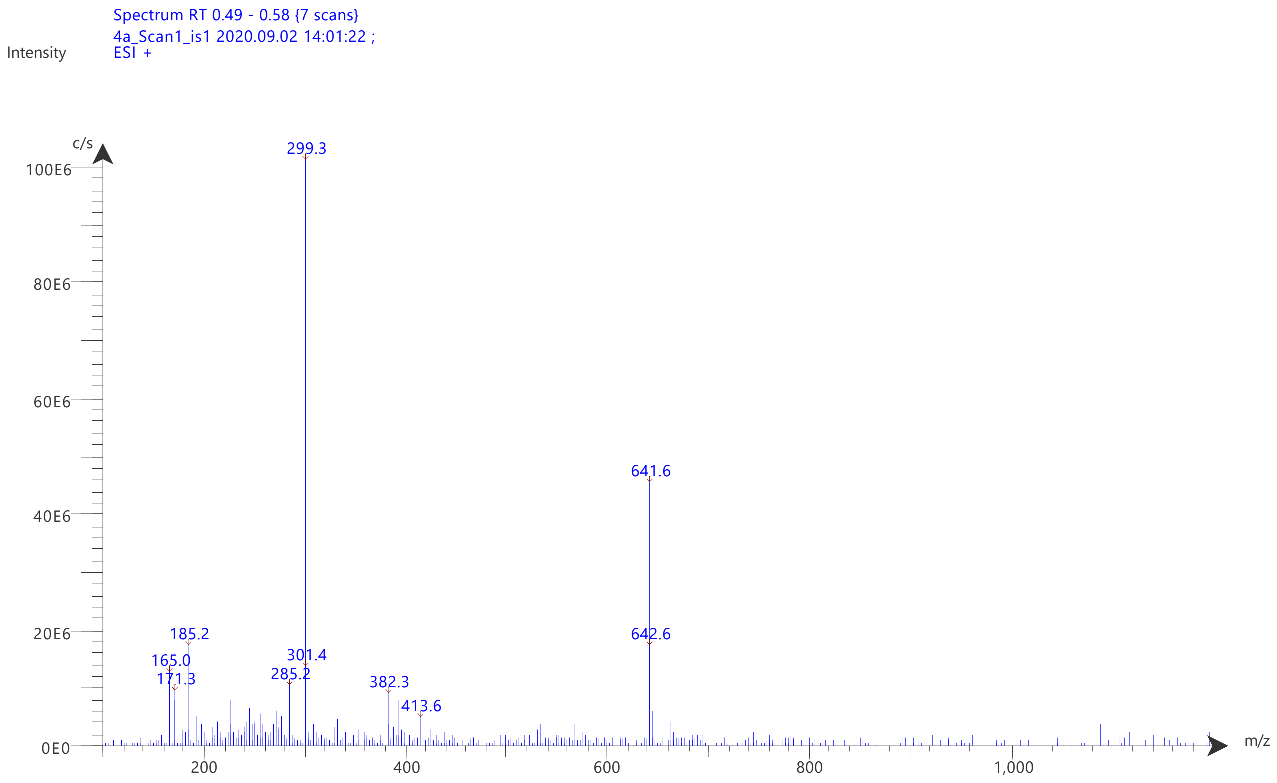

Developmental Therapeutics Program

One Dose Mean Graph 4a

NSC: D-810694 / 1	Conc: 1.00E-5 Molar	Test Date: Dec 10, 2018
Experiment ID: 1812OS42		Report Date: Jan 04, 2019

Panel/Cell Line 4a	Growth Percent
**Leukemia**	
CCRF-CEM	111.09
HL-60(TB)	98.97
K-562	101.05
MOLT-4	102.08
RPMI-8226	47.87
SR	99.60
**Non-Small Cell Lung Cancer**	
A549/ATCC	89.82
EKVX	88.51
HOP-62	78.72
HOP-92	79.94
NCI-H226	69.64
NCI-H23	83.48
NCI-H322M	89.20
NCI-H460	91.19
NCI-H522	89.24
**Colon Cancer**	
COLO 205	109.71
HCC-2998	86.00
HCT-116	-9.74
HCT-15	93.81
HT29	90.79
KM12	84.88
SW-620	90.01
**CNS Cancer**	
SF-268	85.31
SF-295	95.88
SF-539	92.60
SNB-19	98.22
SNB-75	92.69
U251	98.38
**Melanoma**	
LOX IMVI	55.35
MALME-3M	94.94
M14	90.06
MDA-MB-435	96.27
SK-MEL-2	96.38
SK-MEL-28	99.29
SK-MEL-5	84.34
UACC-257	96.69
UACC-62	90.54
**Ovarian Cancer**	
IGROV1	75.32
OVCAR-3	90.44
OVCAR-4	101.95
OVCAR-5	103.37
OVCAR-8	95.11
NCI/ADR-RES	87.92
SK-OV-3	84.92
**Renal Cancer**	
786-0	87.92
A498	76.73
ACHN	96.66
CAKI-1	87.78
RXF 393	72.03
SN12C	102.63
TK-10	102.86
UO-31	63.14
**Prostate Cancer**	
PC-3	83.89
DU-145	103.67
**Breast Cancer**	
MCF7	72.92
MDA-MB-231/ATCC	97.06
HS 578T	80.56
BT-549	102.14
T-47D	96.40
MDA-MB-468	72.51
Mean	87.85
Delta	97.59
Range	120.83

Mean Growth Percent - Growth Percent

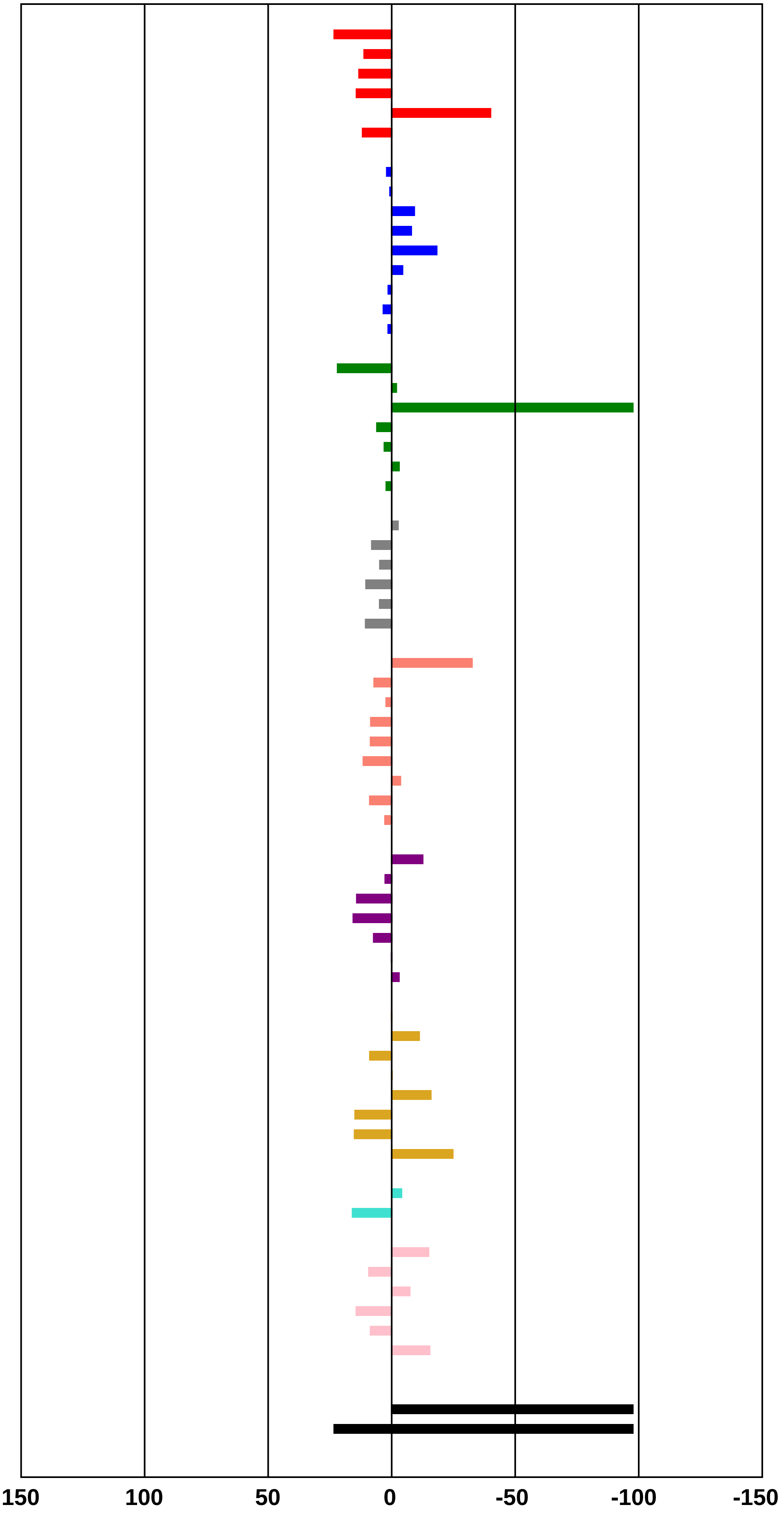

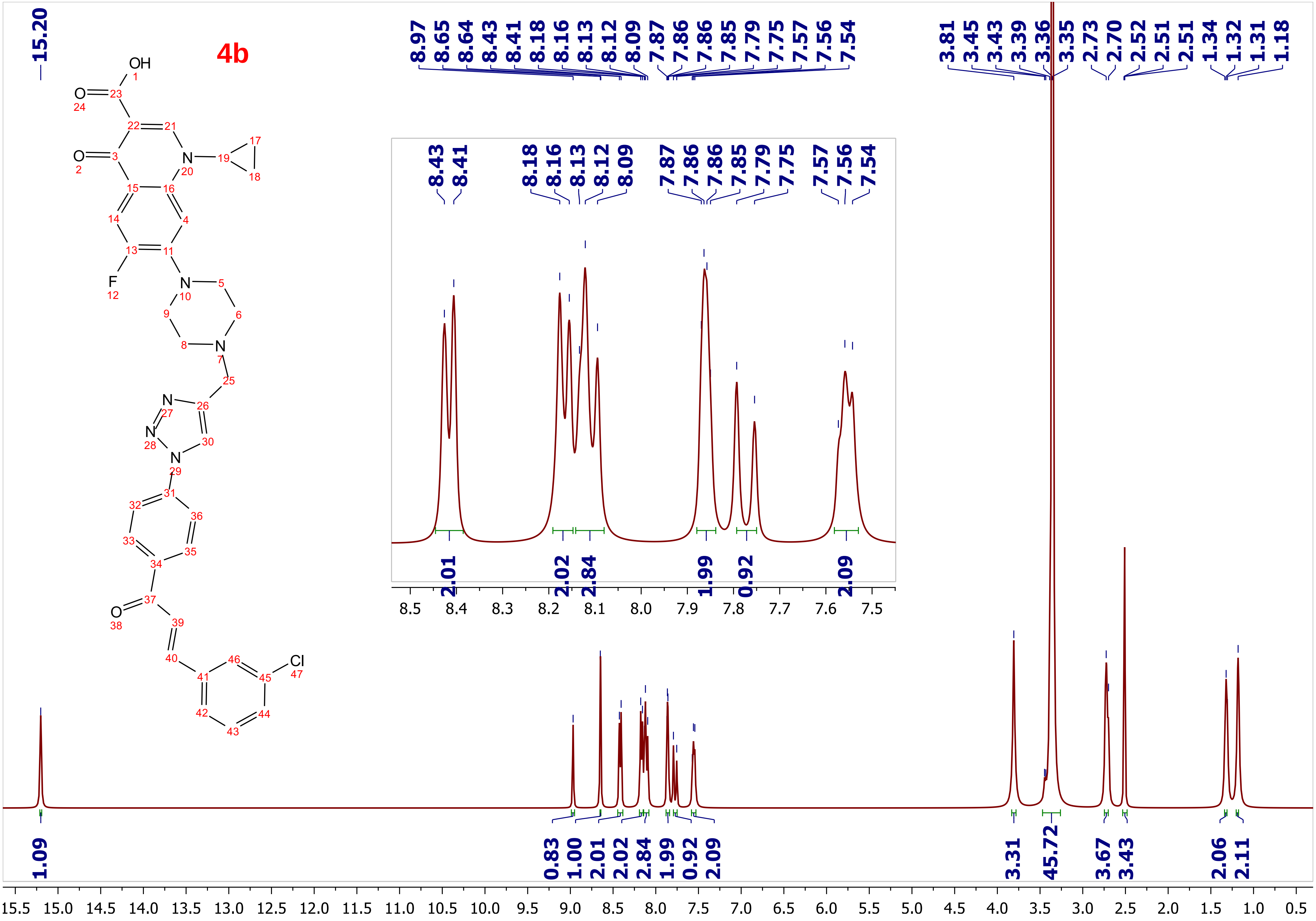
4b
15.20
8.97 8.65 8.64 8.43 8.41 8.18 8.16 8.13 8.12 8.09 7.87 7.86 7.86 7.85 7.79 7.75 7.57 7.56 7.54
3.81 3.45 3.43 3.39 3.36 3.35 2.73 2.70 2.52 2.51 2.51 1.34 1.32 1.31 1.18
1.09 0.83 1.00 2.01 2.02 2.84 1.99 0.92 2.09 3.31 45.72 3.67 3.43 2.06 2.11
15.5 15.0 14.5 14.0 13.5 13.0 12.5 12.0 11.5 11.0 10.5 10.0 9.5 9.0 8.5 8.0 7.5 7.0 6.5 6.0 5.5 5.0 4.5 4.0 3.5 3.0 2.5 2.0 1.5 1.0 0.5
8.43 8.41 8.18 8.16 8.13 8.12 8.09 7.87 7.86 7.86 7.85 7.79 7.75 7.57 7.56 7.54
2.01 2.02 2.84 1.99 0.92 2.09
8.5 8.4 8.3 8.2 8.1 8.0 7.9 7.8 7.7 7.6 7.5

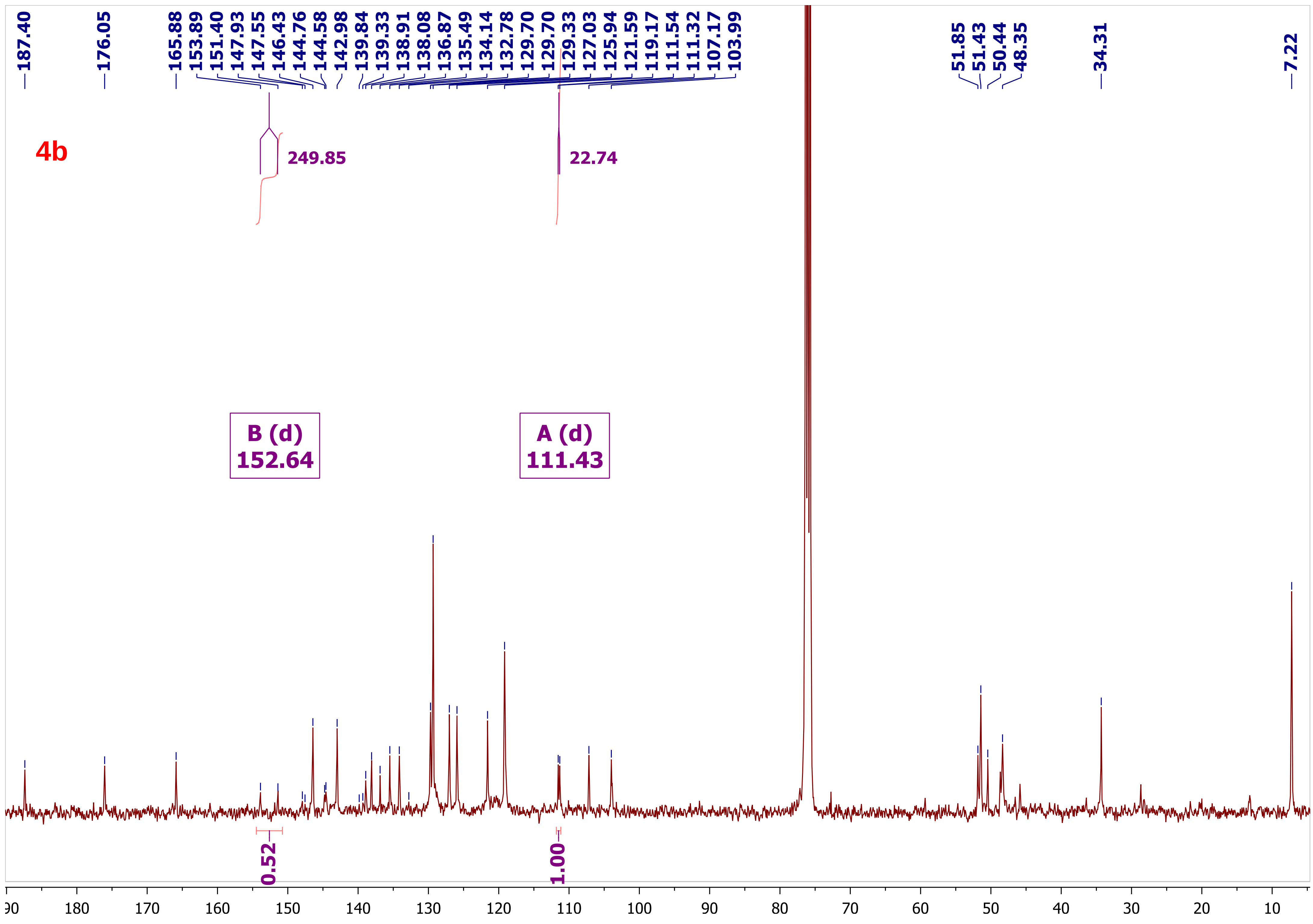

4b
187.40
176.05
165.88
153.89
151.40
147.93
147.55
146.43
144.76
144.58
142.98
139.84
139.33
138.91
138.08
136.87
135.49
134.14
132.78
129.70
129.70
129.33
127.03
125.94
121.59
119.17
111.54
111.32
107.17
103.99
51.85
51.43
50.44
48.35
34.31
7.22
249.85
22.74
B (d)
152.64
A (d)
111.43
0.52
1.00
190
180
170
160
150
140
130
120
110
100
90
80
70
60
50
40
30
20
10

Developmental Therapeutics Program One Dose Mean Graph	NSC: D-810703 / 1	Conc: 1.00E-5 Molar	Test Date: Dec 10, 2018
	Experiment ID: 1812OS42		Report Date: Jan 04, 2019

Panel/Cell Line 4b | **Growth Percent** | **Mean Growth Percent - Growth Percent**

Panel/Cell Line	Growth Percent
Leukemia	
CCRF-CEM	29.87
HL-60(TB)	90.26
K-562	40.53
MOLT-4	68.19
RPMI-8226	-14.59
SR	14.12
Non-Small Cell Lung Cancer	
A549/ATCC	71.61
EKVX	85.00
HOP-62	67.65
HOP-92	79.32
NCI-H226	82.12
NCI-H23	91.39
NCI-H322M	80.23
NCI-H460	66.65
NCI-H522	83.92
Colon Cancer	
COLO 205	106.25
HCC-2998	59.31
HCT-116	-53.36
HCT-15	71.65
HT29	53.31
KM12	29.91
SW-620	85.42
CNS Cancer	
SF-268	56.37
SF-295	80.19
SF-539	60.12
SNB-19	53.84
SNB-75	89.22
U251	34.32
Melanoma	
LOX IMVI	36.92
MALME-3M	94.48
M14	79.03
MDA-MB-435	74.02
SK-MEL-2	93.04
SK-MEL-28	95.79
SK-MEL-5	78.72
UACC-257	87.73
UACC-62	61.16
Ovarian Cancer	
IGROV1	51.84
OVCAR-3	57.34
OVCAR-4	62.88
OVCAR-5	84.72
OVCAR-8	42.70
NCI/ADR-RES	94.24
SK-OV-3	72.31
Renal Cancer	
786-0	53.90
A498	70.85
ACHN	77.70
CAKI-1	52.54
RXF 393	70.85
SN12C	37.63
TK-10	84.89
UO-31	52.64
Prostate Cancer	
PC-3	73.99
DU-145	77.52
Breast Cancer	
MCF7	37.62
MDA-MB-231/ATCC	73.61
HS 578T	73.69
BT-549	90.39
T-47D	75.28
MDA-MB-468	64.77
Mean	64.96
Delta	118.32
Range	159.61

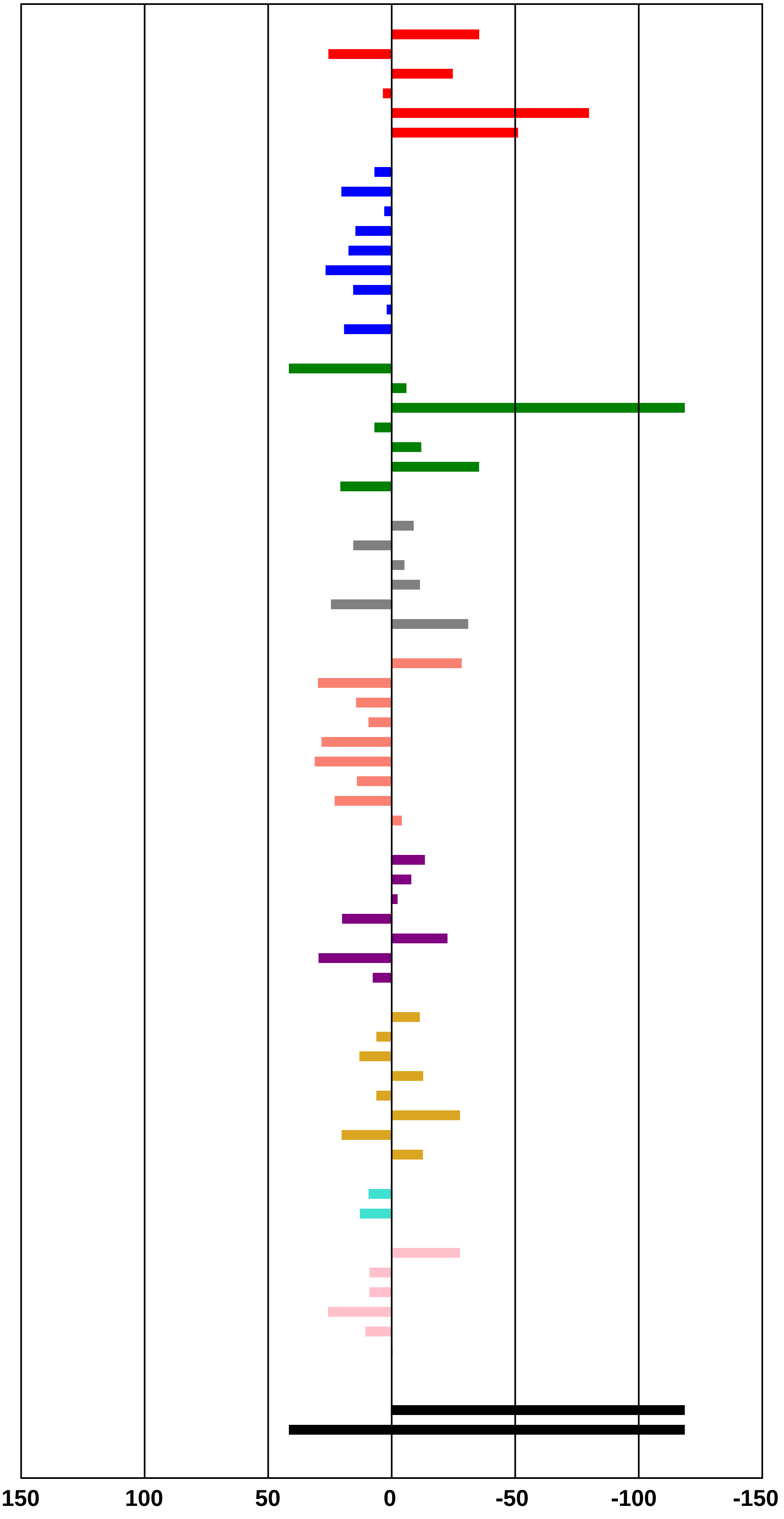

4c

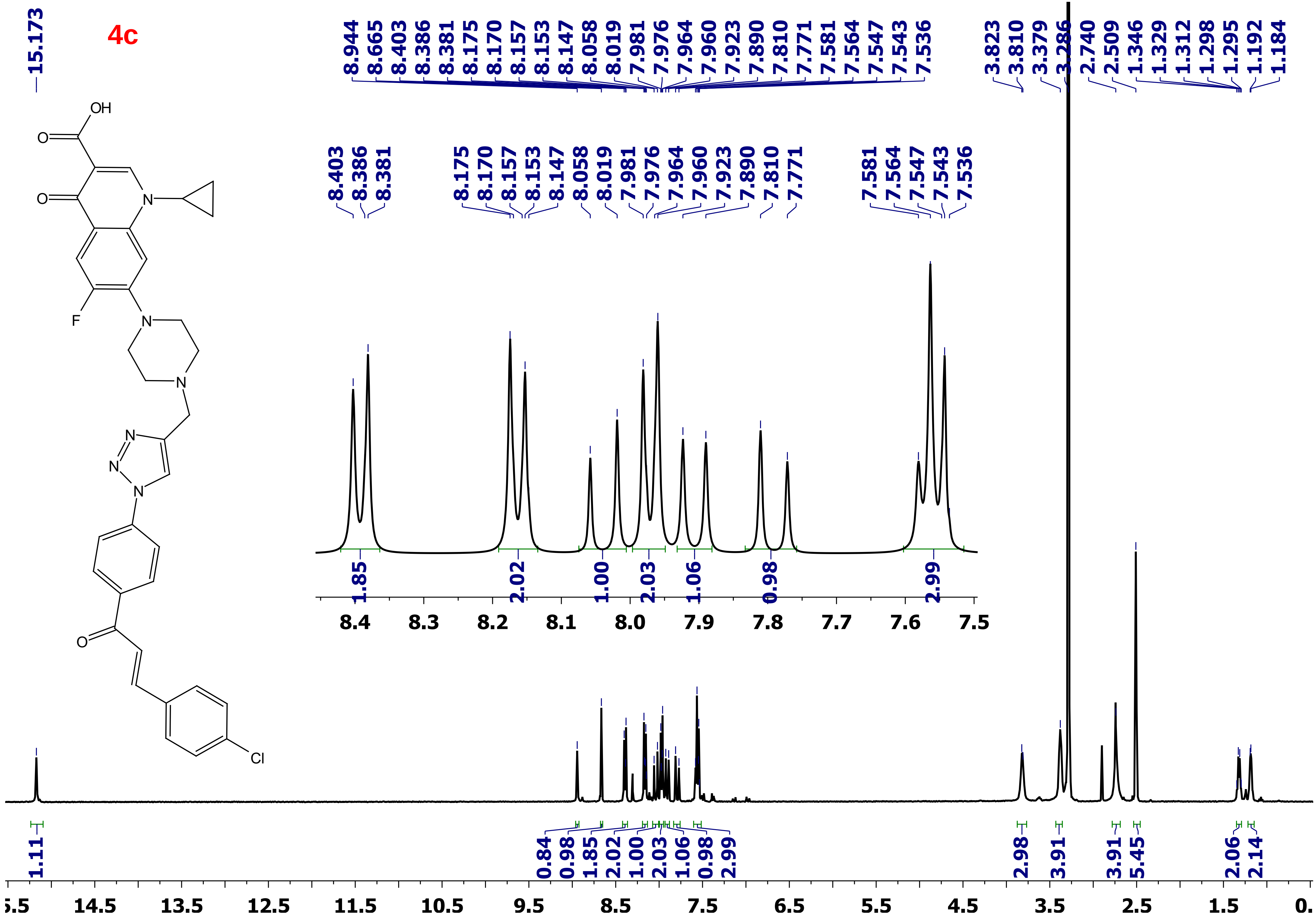

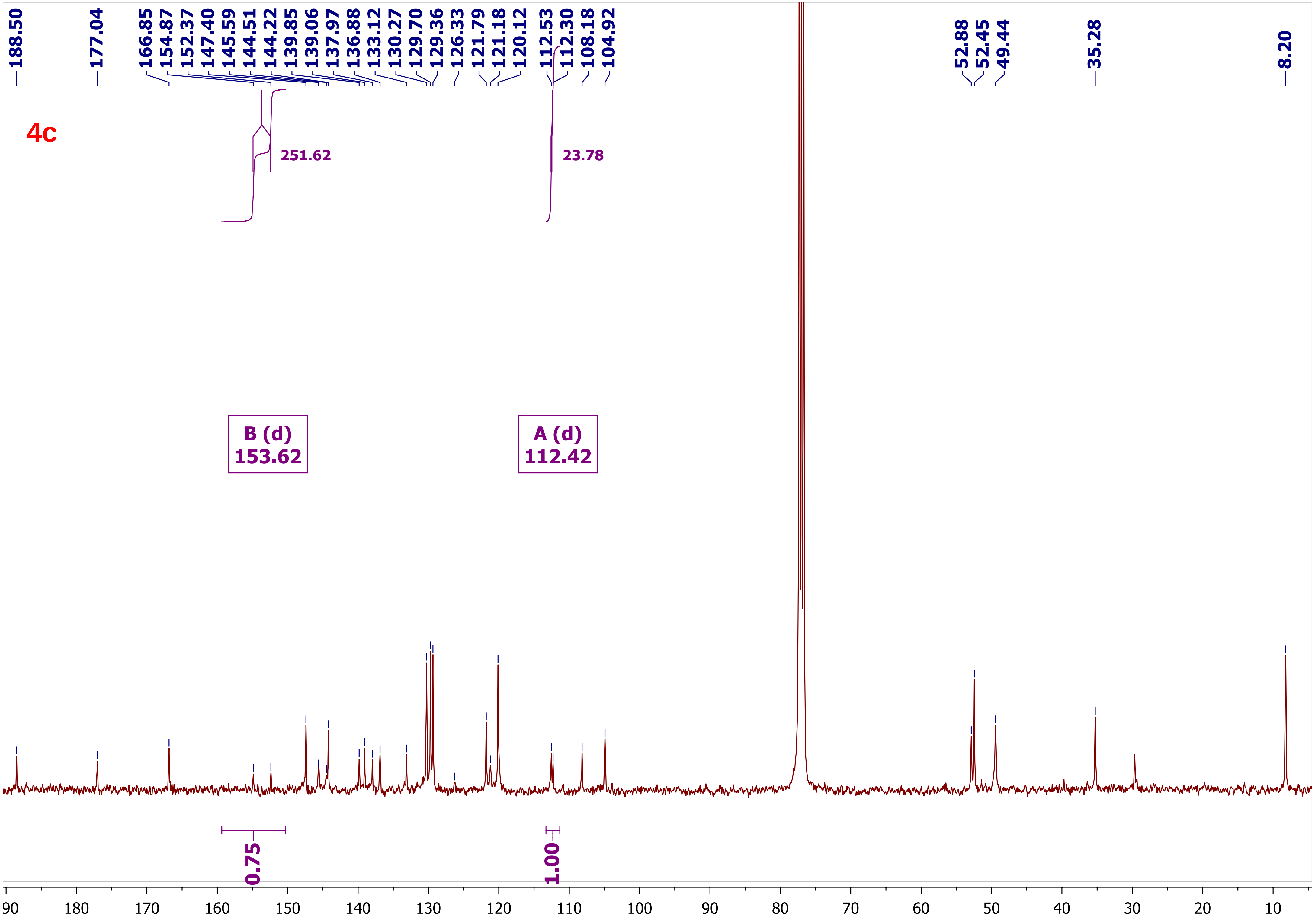

4c
188.50
177.04
166.85
154.87
152.37
147.40
145.59
144.51
144.22
139.85
139.06
137.97
136.88
133.12
130.27
129.70
129.36
126.33
121.79
121.18
120.12
112.53
112.30
108.18
104.92
52.88
52.45
49.44
35.28
8.20
251.62
23.78
B (d)
153.62
A (d)
112.42
0.75
1.00
90
180
170
160
150
140
130
120
110
100
90
80
70
60
50
40
30
20
10

4c

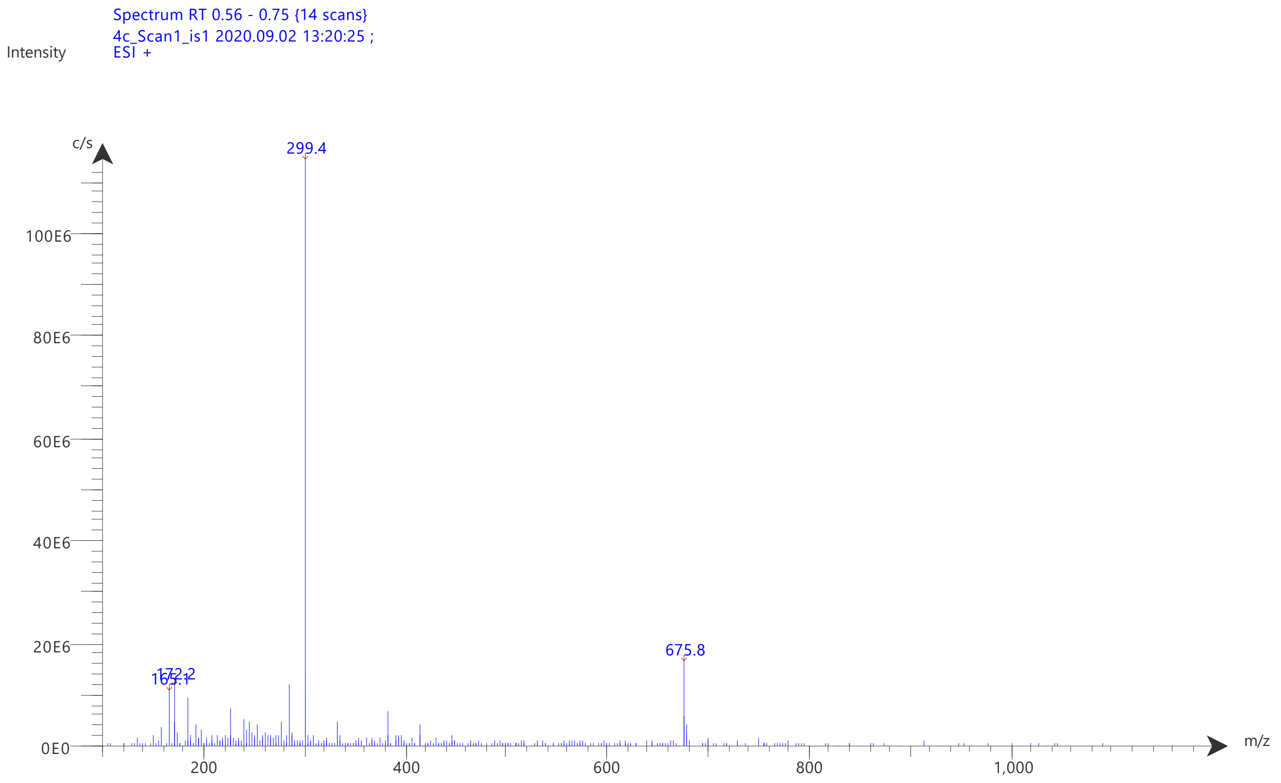
Spectrum RT 0.56 - 0.75 {14 scans}
4c_Scan1_is1 2020.09.02 13:20:25 ;
ESI +
Intensity
c/s
100E6
80E6
60E6
40E6
20E6
0E0
299.4
172.2
165.1
675.8
200
400
600
800
1,000
m/z

Developmental Therapeutics Program
One Dose Mean Graph

NSC: D-810695 / 1	Conc: 1.00E-5 Molar	Test Date: Dec 10, 2018
Experiment ID: 1812OS42		Report Date: Jan 04, 2019

4c

Panel/Cell Line	Growth Percent
**Leukemia**	
CCRF-CEM	64.91
HL-60(TB)	99.09
K-562	55.95
MOLT-4	84.10
RPMI-8226	-0.51
SR	24.05
**Non-Small Cell Lung Cancer**	
A549/ATCC	88.94
EKVX	93.98
HOP-62	79.60
HOP-92	79.56
NCI-H226	72.99
NCI-H23	79.66
NCI-H322M	84.44
NCI-H460	76.09
NCI-H522	80.13
**Colon Cancer**	
COLO 205	107.70
HCC-2998	88.33
HCT-116	6.95
HCT-15	91.63
HT29	79.17
KM12	73.73
SW-620	83.72
**CNS Cancer**	
SF-268	70.36
SF-295	91.67
SF-539	68.97
SNB-19	71.98
SNB-75	72.27
U251	42.15
**Melanoma**	
LOX IMVI	36.24
MALME-3M	91.17
M14	84.41
MDA-MB-435	79.45
SK-MEL-2	96.79
SK-MEL-28	88.18
SK-MEL-5	77.61
UACC-257	91.00
UACC-62	73.84
**Ovarian Cancer**	
IGROV1	83.96
OVCAR-3	91.49
OVCAR-4	89.70
OVCAR-5	92.16
OVCAR-8	62.04
NCI/ADR-RES	87.03
SK-OV-3	80.03
**Renal Cancer**	
786-0	54.18
A498	52.18
ACHN	93.49
CAKI-1	61.40
RXF 393	58.30
SN12C	45.81
TK-10	83.31
UO-31	62.47
**Prostate Cancer**	
PC-3	79.29
DU-145	76.28
**Breast Cancer**	
MCF7	58.18
MDA-MB-231/ATCC	89.42
HS 578T	67.40
BT-549	76.68
T-47D	92.92
MDA-MB-468	66.04
Mean	73.90
Delta	74.41
Range	108.21

Mean Growth Percent - Growth Percent

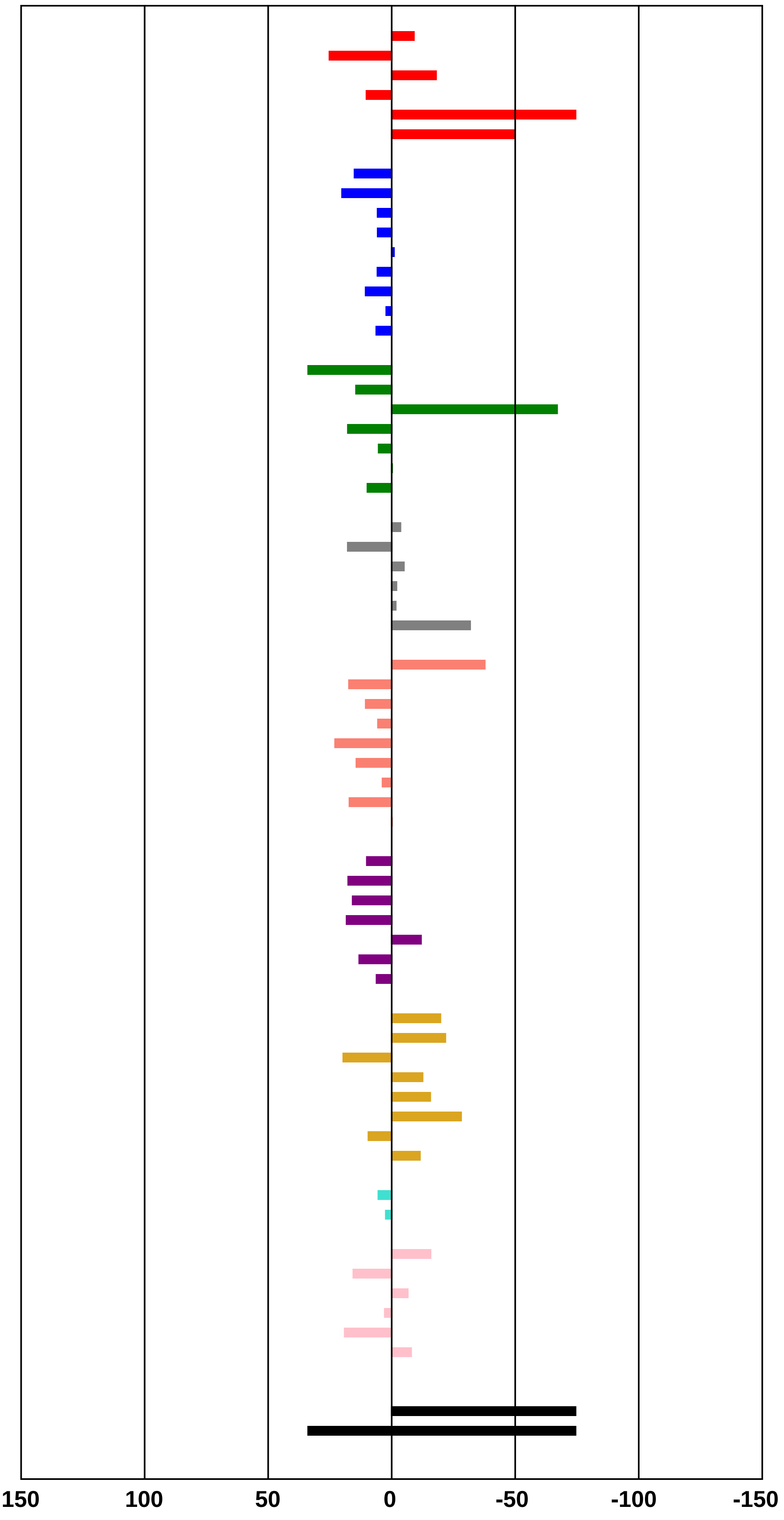

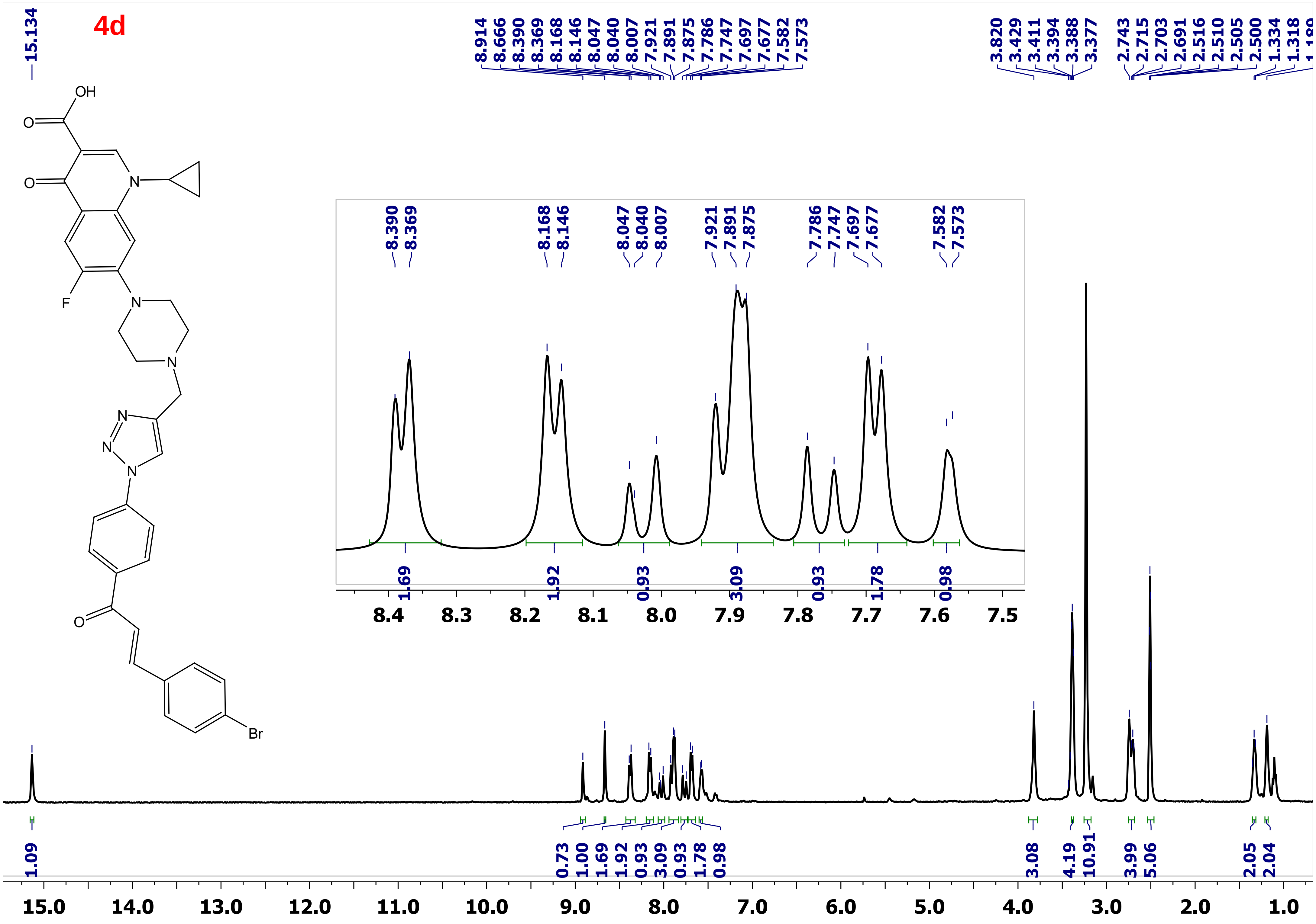
4d
OH
O
O
N
F
N
N
N
N
N
O
Br
15.134
8.914
8.666
8.390
8.369
8.168
8.146
8.047
8.040
8.007
7.921
7.891
7.875
7.786
7.747
7.697
7.677
7.582
7.573
3.820
3.429
3.411
3.394
3.388
3.377
2.743
2.715
2.703
2.691
2.516
2.510
2.505
2.500
1.334
1.318
1.69
1.92
0.93
3.09
0.93
1.78
0.98
8.4
8.3
8.2
8.1
8.0
7.9
7.8
7.7
7.6
7.5
1.09
0.73
1.00
1.69
1.92
0.93
3.09
0.93
1.78
0.98
3.08
4.19
10.91
3.99
5.06
2.05
2.04
15.0
14.0
13.0
12.0
11.0
10.0
9.0
8.0
7.0
6.0
5.0
4.0
3.0
2.0
1.0

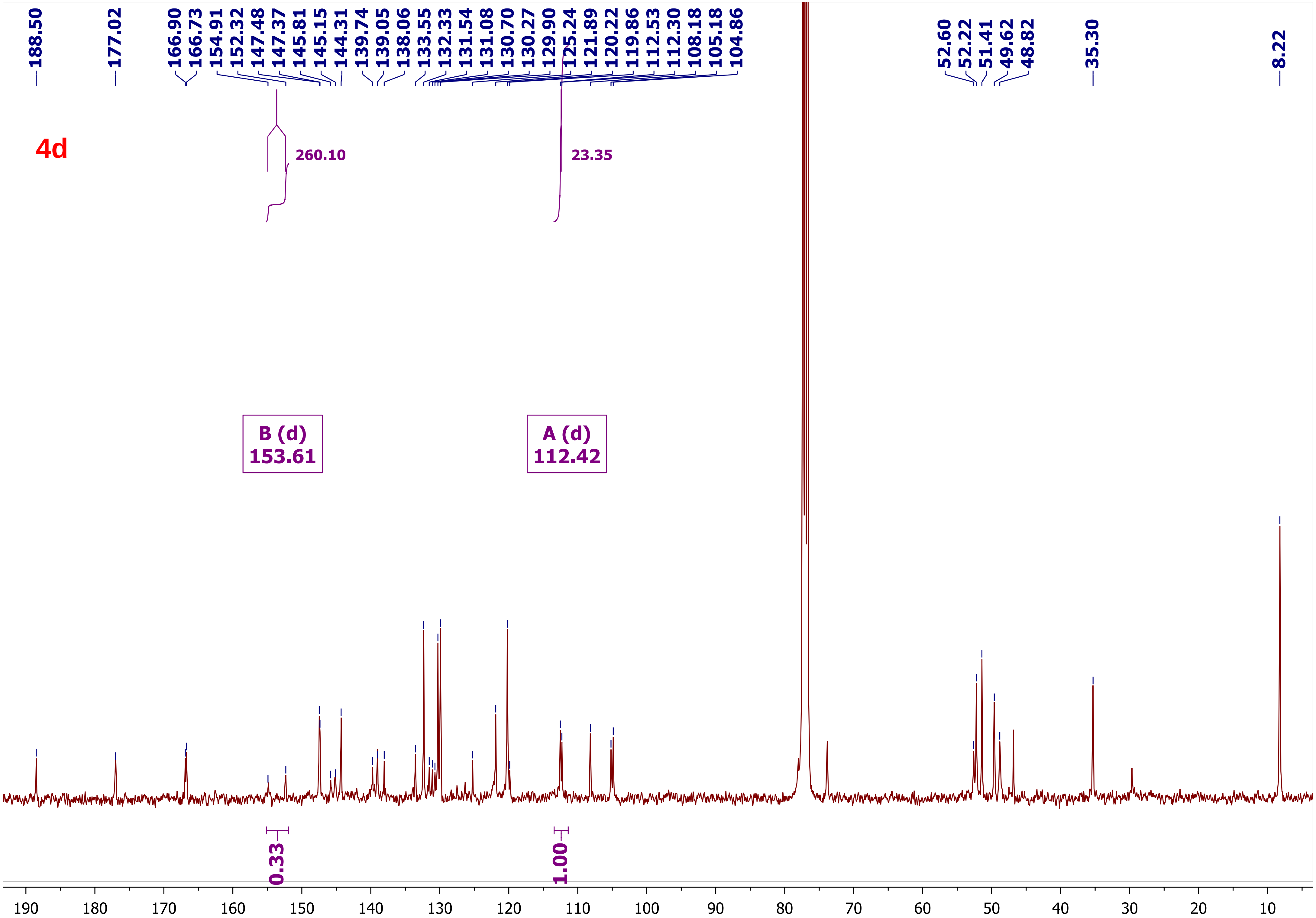

4d
188.50
177.02
166.90
166.73
154.91
152.32
147.48
147.37
145.81
145.15
144.31
139.74
139.05
138.06
133.55
132.33
131.54
131.08
130.70
130.27
129.90
125.24
121.89
120.22
119.86
112.53
112.30
108.18
105.18
104.86
52.60
52.22
51.41
49.62
48.82
35.30
8.22
260.10
23.35
B (d)
153.61
A (d)
112.42
0.33
1.00
190
180
170
160
150
140
130
120
110
100
90
80
70
60
50
40
30
20
10

4d

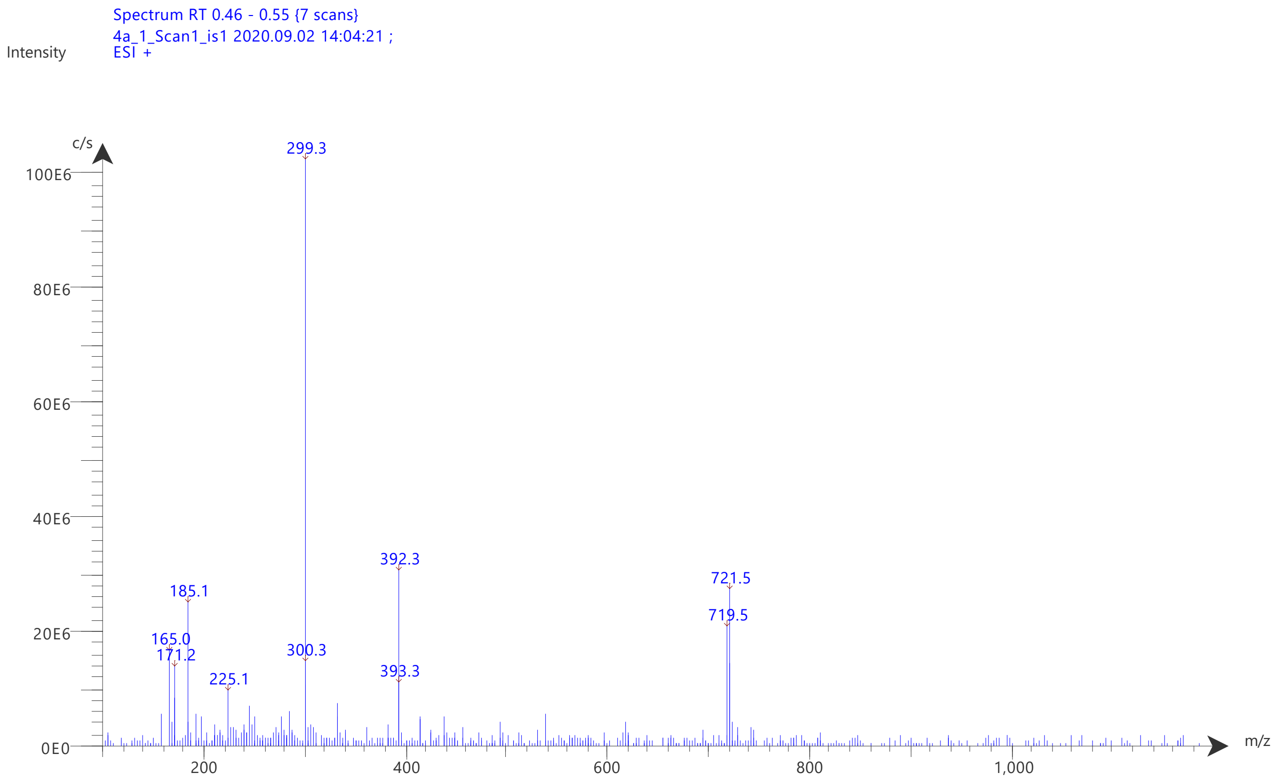
Spectrum RT 0.46 - 0.55 {7 scans}
4a_1_Scan1_is1 2020.09.02 14:04:21 ;
ESI +
Intensity
c/s
100E6
80E6
60E6
40E6
20E6
0E0
299.3
392.3
185.1
721.5
719.5
165.0
171.2
300.3
393.3
225.1
200
400
600
800
1,000
m/z

Developmental Therapeutics Program	NSC: D-810699 / 1	Conc: 1.00E-5 Molar	Test Date: Dec 10, 2018
One Dose Mean Graph	Experiment ID: 1812OS42		Report Date: Jan 04, 2019

4d

Panel/Cell Line	Growth Percent
Leukemia	
CCRF-CEM	34.65
HL-60(TB)	98.82
K-562	60.70
MOLT-4	80.43
RPMI-8226	6.02
SR	21.27
Non-Small Cell Lung Cancer	
A549/ATCC	90.47
EKVX	80.83
HOP-62	85.39
HOP-92	87.96
NCI-H226	74.77
NCI-H23	77.57
NCI-H322M	88.80
NCI-H460	85.94
NCI-H522	87.97
Colon Cancer	
COLO 205	111.68
HCC-2998	83.26
HCT-116	17.31
HCT-15	90.56
HT29	99.25
KM12	87.09
SW-620	85.92
CNS Cancer	
SF-268	77.21
SF-295	81.91
SF-539	82.46
SNB-19	76.67
SNB-75	85.67
U251	68.44
Melanoma	
LOX IMVI	55.72
MALME-3M	87.73
M14	88.12
MDA-MB-435	81.90
SK-MEL-2	102.16
SK-MEL-28	91.98
SK-MEL-5	79.72
UACC-257	90.10
UACC-62	72.76
Ovarian Cancer	
IGROV1	79.36
OVCAR-3	84.94
OVCAR-4	96.72
OVCAR-5	92.67
OVCAR-8	80.72
NCI/ADR-RES	85.63
SK-OV-3	78.97
Renal Cancer	
786-0	72.23
A498	34.90
ACHN	93.40
CAKI-1	68.68
RXF 393	55.69
SN12C	58.84
TK-10	97.80
UO-31	65.40
Prostate Cancer	
PC-3	84.67
DU-145	92.28
Breast Cancer	
MCF7	61.43
MDA-MB-231/ATCC	66.37
HS 578T	71.25
BT-549	92.52
T-47D	100.53
MDA-MB-468	72.48
Mean	77.44
Delta	71.42
Range	105.66

Mean Growth Percent - Growth Percent

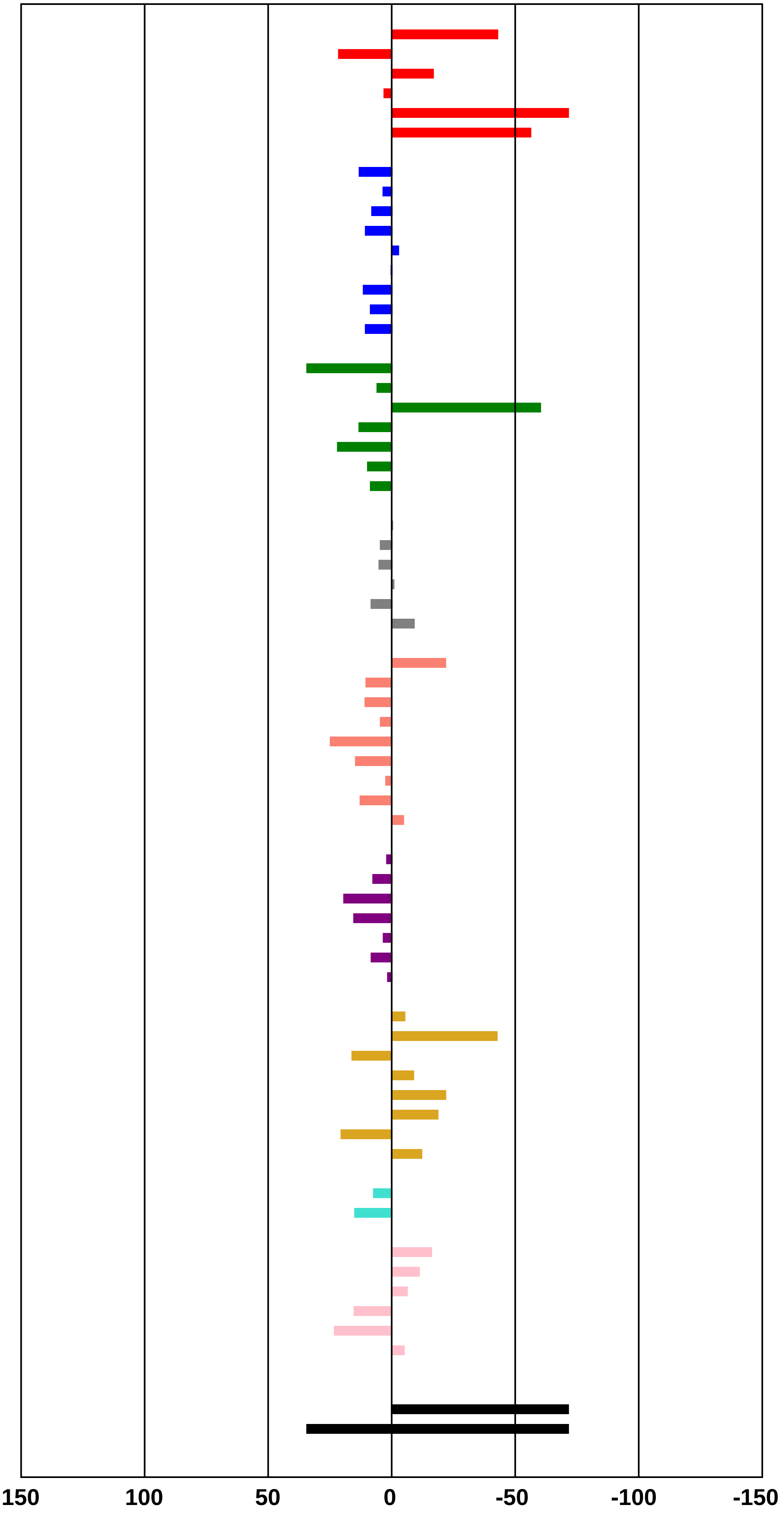

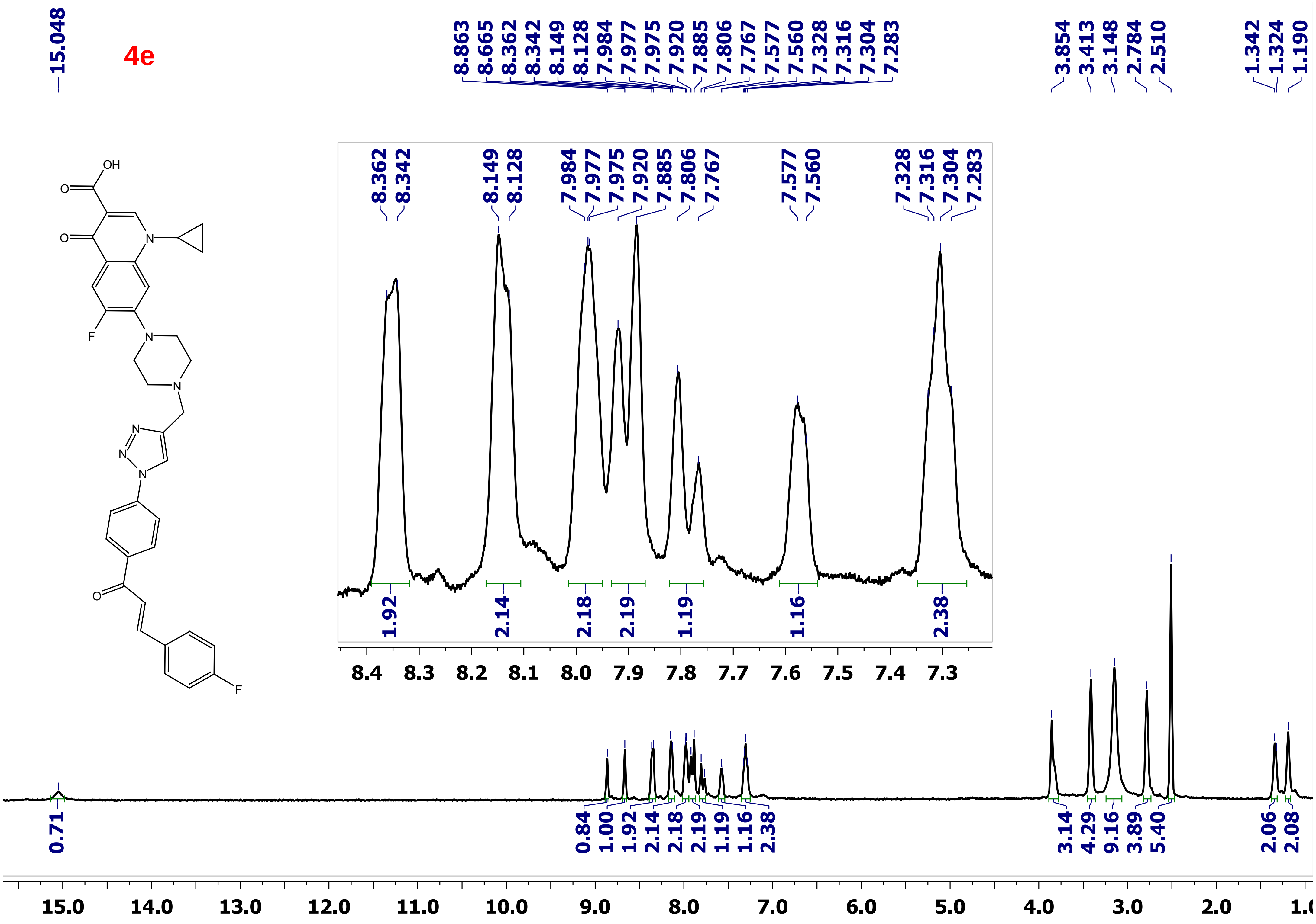

4e
15.048
8.863 8.665 8.362 8.342 8.149 8.128 7.984 7.977 7.975 7.920 7.885 7.806 7.767 7.577 7.560 7.328 7.316 7.304 7.283
3.854 3.413 3.148 2.784 2.510
1.342 1.324 1.190
1.92 2.14 2.18 2.19 1.19 1.16 2.38
8.4 8.3 8.2 8.1 8.0 7.9 7.8 7.7 7.6 7.5 7.4 7.3
0.71
0.84 1.00 1.92 2.14 2.18 2.19 1.19 1.16 2.38
3.14 4.29 9.16 3.89 5.40
2.06 2.08
15.0 14.0 13.0 12.0 11.0 10.0 9.0 8.0 7.0 6.0 5.0 4.0 3.0 2.0
OH
O
N
F
N
N
N
N
N
O
F

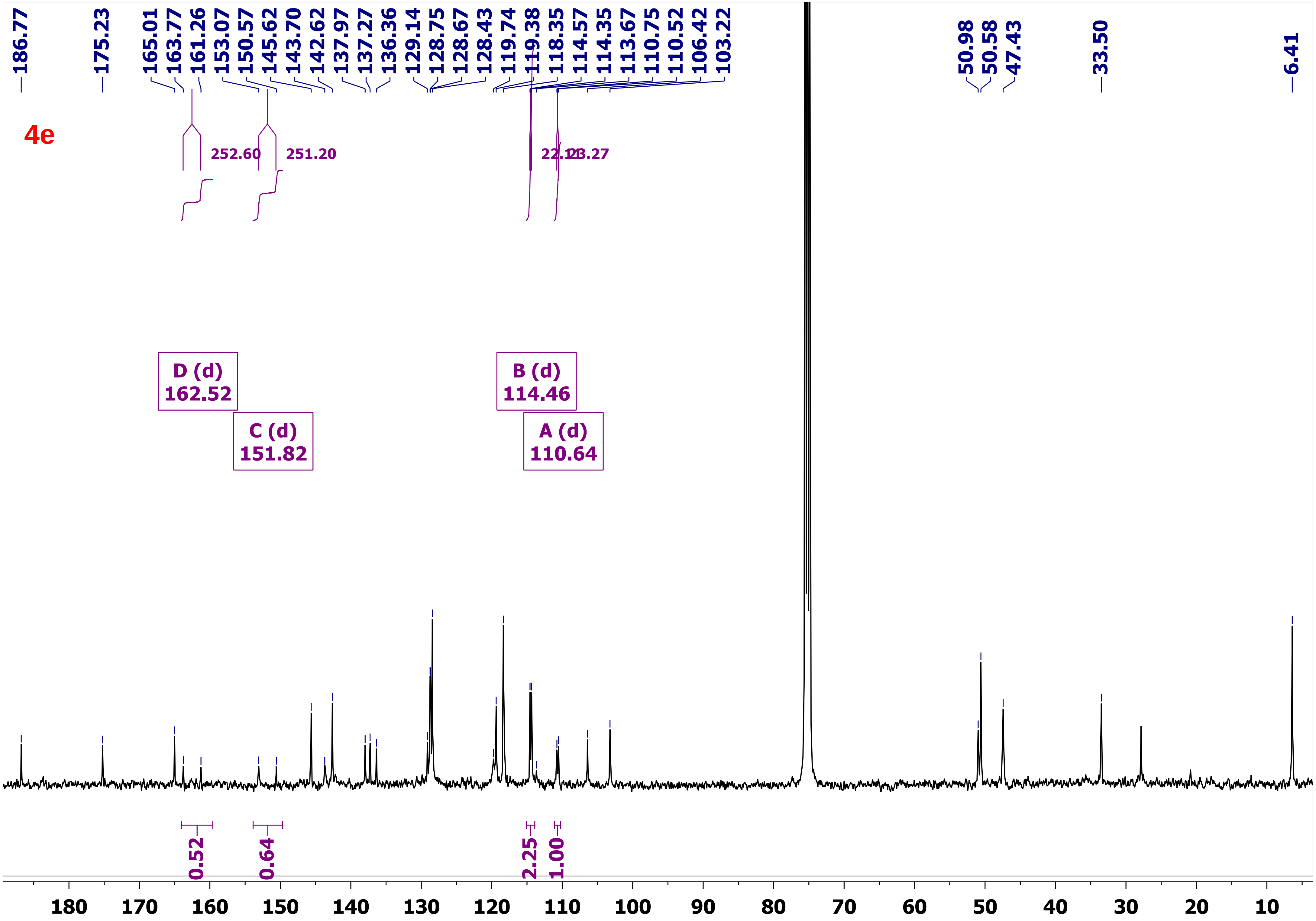

4e
186.77
175.23
165.01
163.77
161.26
153.07
150.57
145.62
143.70
142.62
137.97
137.27
136.36
129.14
128.75
128.67
128.43
119.74
119.38
118.35
114.57
114.35
113.67
110.75
110.52
106.42
103.22
50.98
50.58
47.43
33.50
6.41
252.60
251.20
D (d)
162.52
C (d)
151.82
B (d)
114.46
A (d)
110.64
0.52
0.64
2.25
1.00
180
170
160
150
140
130
120
110
100
90
80
70
60
50
40
30
20
10

4e

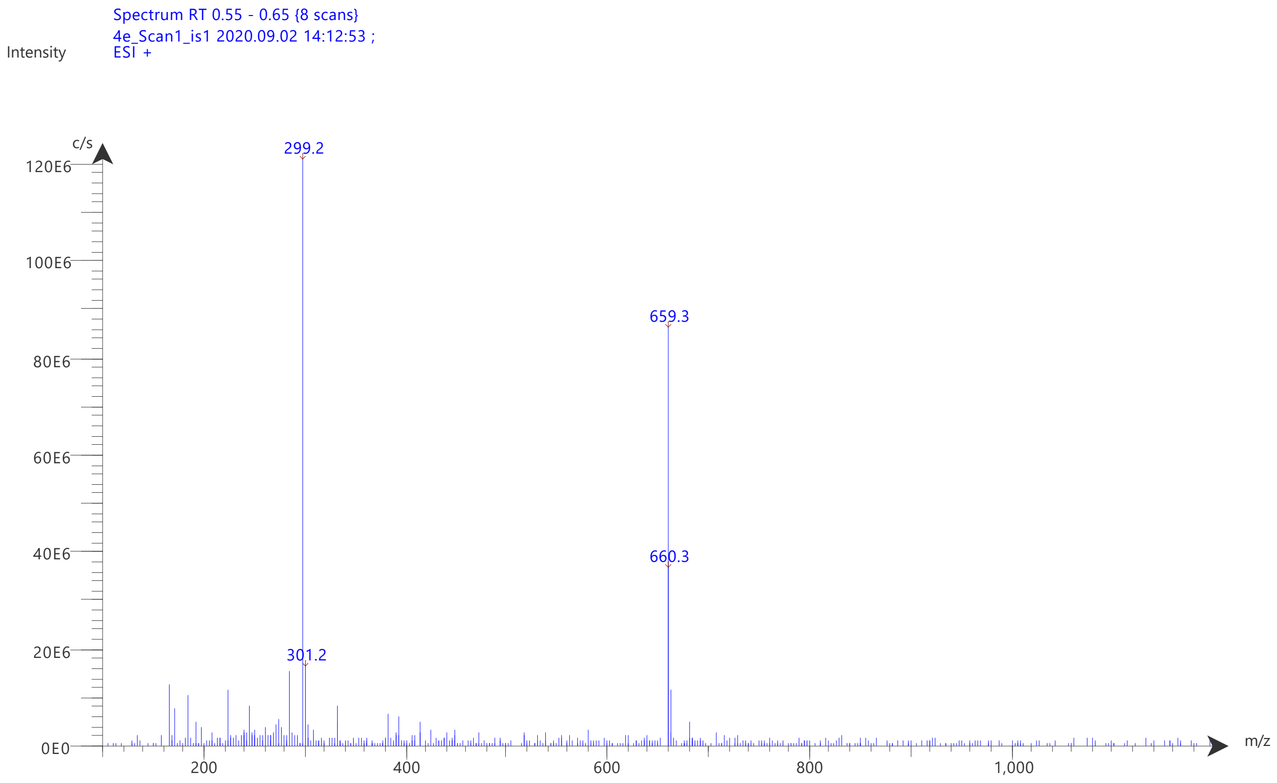
Spectrum RT 0.55 - 0.65 {8 scans}
4e_Scan1_is1 2020.09.02 14:12:53 ;
ESI +
Intensity
c/s
120E6
100E6
80E6
60E6
40E6
20E6
0E0
299.2
659.3
660.3
301.2
200
400
600
800
1,000
m/z

Developmental Therapeutics Program

One Dose Mean Graph

NSC: D-810702 / 1	Conc: 1.00E-5 Molar	Test Date: Dec 10, 2018
Experiment ID: 1812OS42		Report Date: Jan 04, 2019

4e

Panel/Cell Line	Growth Percent
Leukemia	
CCRF-CEM	20.02
HL-60(TB)	84.59
K-562	21.93
MOLT-4	31.84
RPMI-8226	-24.56
SR	7.11
Non-Small Cell Lung Cancer	
A549/ATCC	82.20
EKVX	87.55
HOP-62	67.26
HOP-92	50.26
NCI-H226	81.69
NCI-H23	80.31
NCI-H322M	77.50
NCI-H460	32.47
NCI-H522	48.51
Colon Cancer	
COLO 205	104.91
HCC-2998	79.29
HCT-116	-10.26
HCT-15	63.44
HT29	28.02
KM12	32.89
SW-620	43.24
CNS Cancer	
SF-268	35.69
SF-295	81.21
SF-539	27.95
SNB-19	31.59
SNB-75	68.09
U251	16.55
Melanoma	
LOX IMVI	20.34
MALME-3M	89.20
M14	60.87
MDA-MB-435	64.66
SK-MEL-2	88.96
SK-MEL-28	67.99
SK-MEL-5	79.01
UACC-257	88.30
UACC-62	55.80
Ovarian Cancer	
IGROV1	39.56
OVCAR-3	46.63
OVCAR-4	93.13
OVCAR-5	84.15
OVCAR-8	23.34
NCI/ADR-RES	92.11
SK-OV-3	71.61
Renal Cancer	
786-0	33.25
A498	61.14
ACHN	88.48
CAKI-1	36.86
RXF 393	44.97
SN12C	8.77
TK-10	85.15
UO-31	62.79
Prostate Cancer	
PC-3	57.38
DU-145	51.65
Breast Cancer	
MCF7	24.23
MDA-MB-231/ATCC	65.28
HS 578T	57.45
BT-549	31.99
T-47D	76.76
MDA-MB-468	32.87
Mean	53.93
Delta	78.49
Range	129.47

Mean Growth Percent - Growth Percent

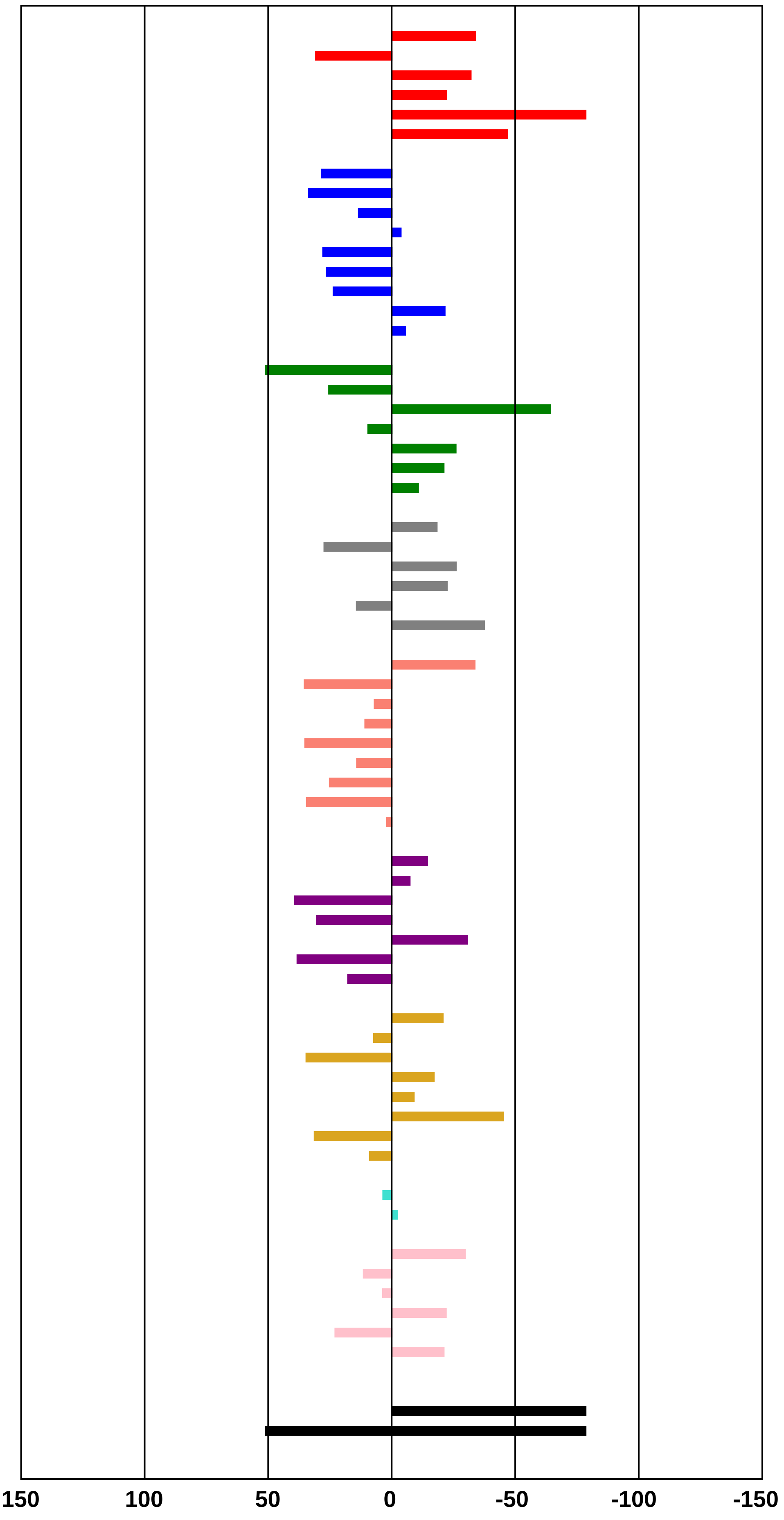

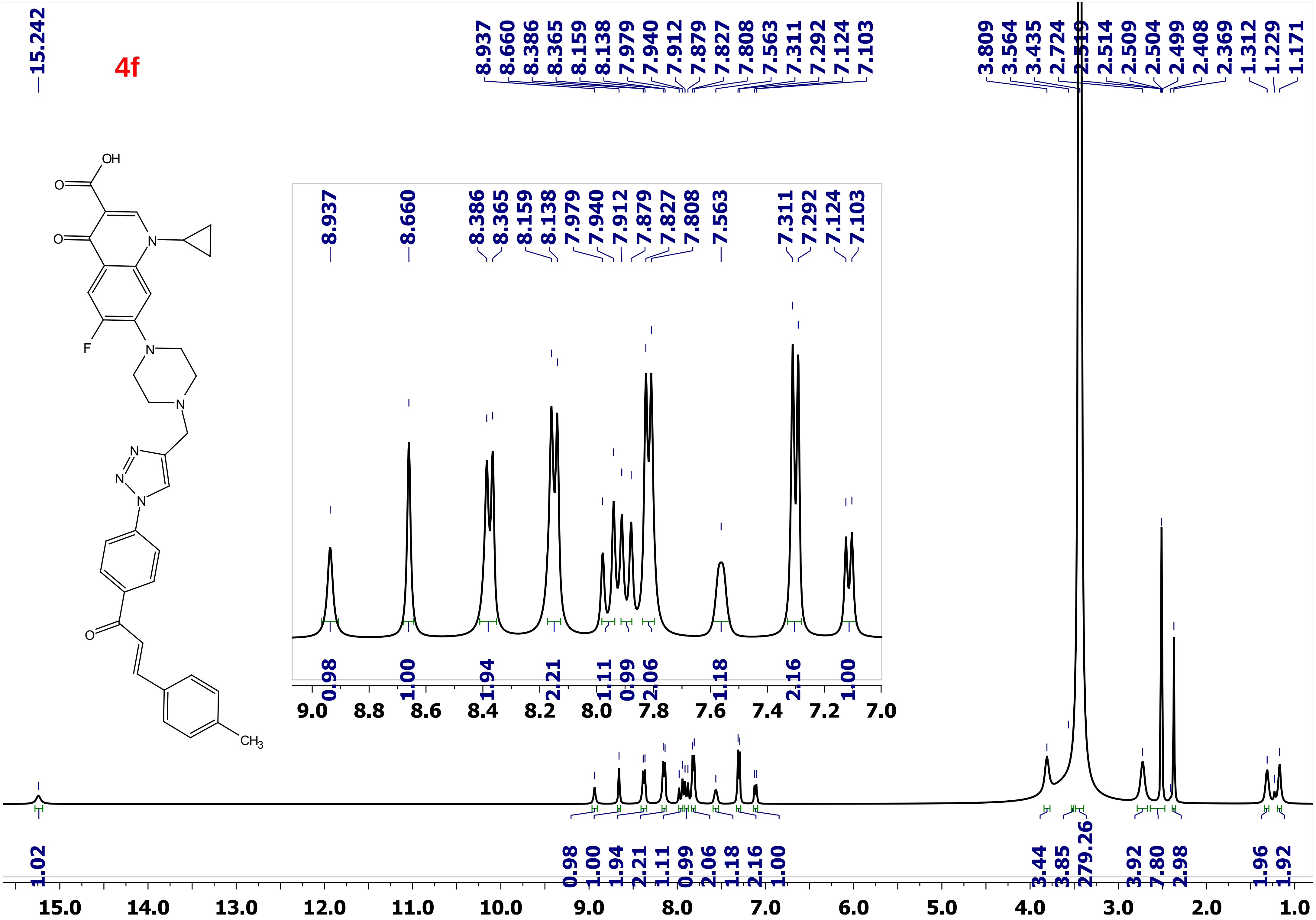

4f
15.242
8.937 8.660 8.386 8.365 8.159 8.138 7.979 7.940 7.912 7.879 7.827 7.808 7.563 7.311 7.292 7.124 7.103
3.809 3.564 3.435 2.724 2.519 2.514 2.509 2.504 2.499 2.408 2.369 1.312 1.229 1.171
OH
O
O
N
F
N
N
N
N
N
O
CH3
0.98 1.00 1.94 2.21 1.11 0.99 2.06 1.18 2.16 1.00
9.0 8.8 8.6 8.4 8.2 8.0 7.8 7.6 7.4 7.2 7.0
1.02
0.98 1.00 1.94 2.21 1.11 0.99 2.06 1.18 2.16 1.00
3.44 3.85 279.26 3.92 7.80 2.98 1.96 1.92
15.0 14.0 13.0 12.0 11.0 10.0 9.0 8.0 7.0 6.0 5.0 4.0 3.0 2.0 1.0

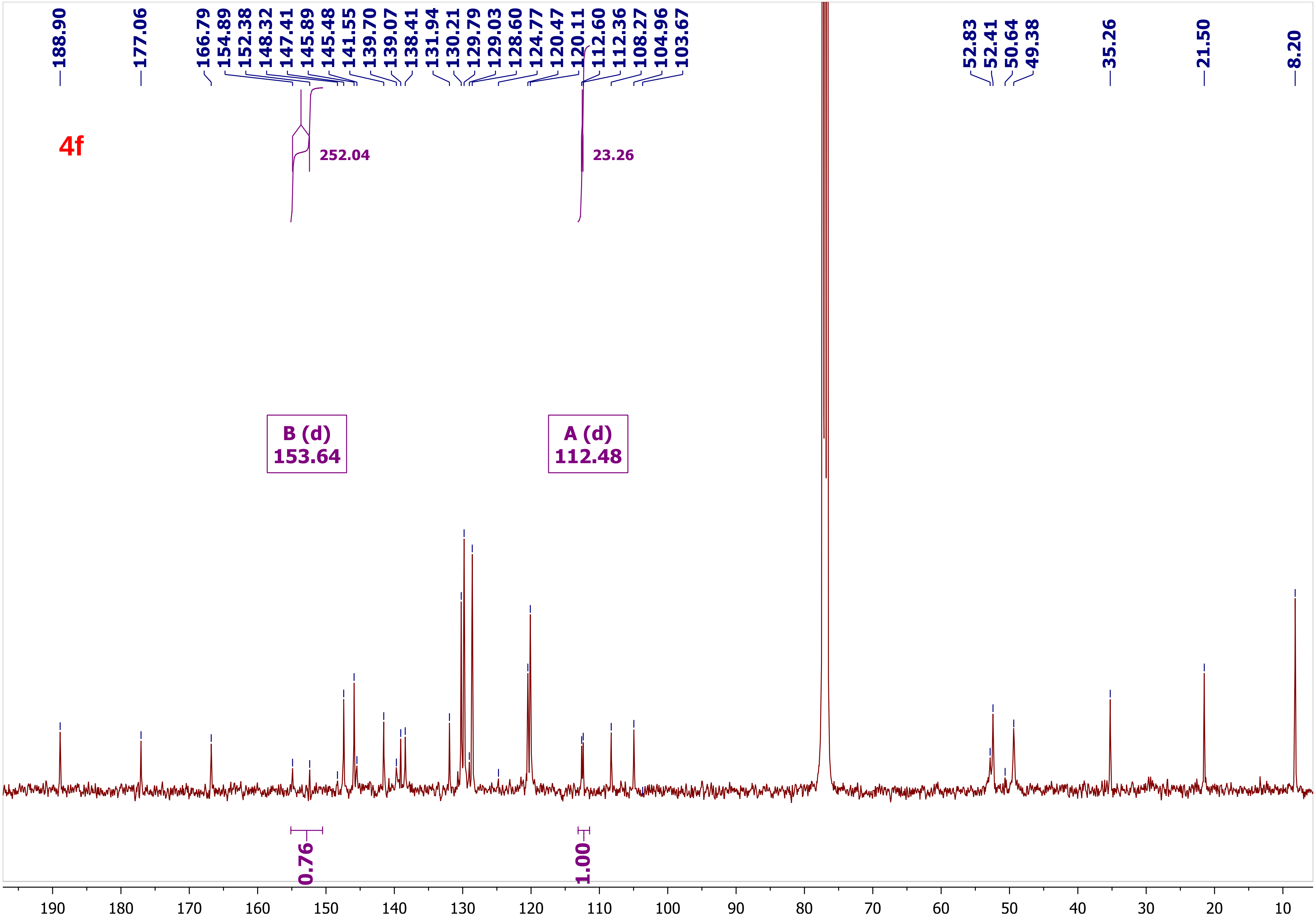
4f
188.90
177.06
166.79
154.89
152.38
148.32
147.41
145.89
145.48
141.55
139.70
139.07
138.41
131.94
130.21
129.79
129.03
128.60
124.77
120.47
120.11
112.60
112.36
108.27
104.96
103.67
52.83
52.41
50.64
49.38
35.26
21.50
8.20
252.04
23.26
B (d)
153.64
A (d)
112.48
0.76
1.00
190
180
170
160
150
140
130
120
110
100
90
80
70
60
50
40
30
20
10

4f

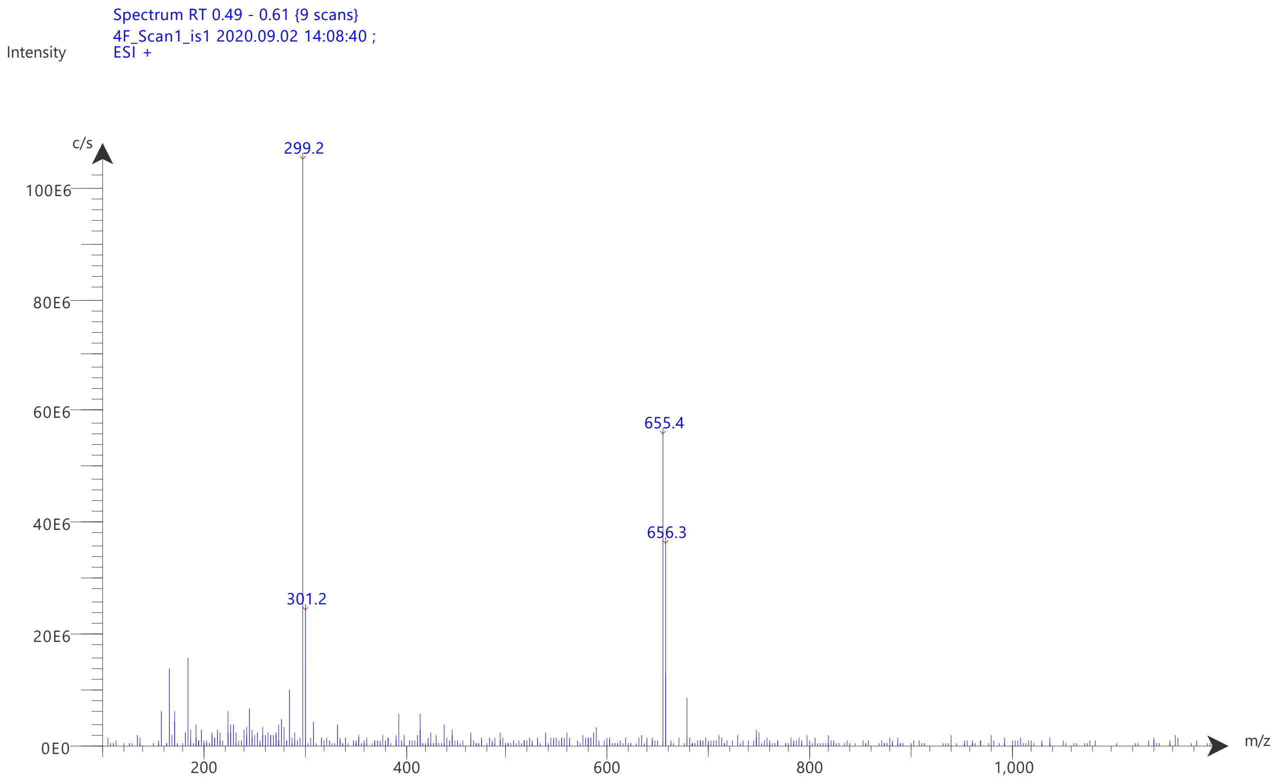
Spectrum RT 0.49 - 0.61 {9 scans}
4F_Scan1_is1 2020.09.02 14:08:40 ;
ESI +
Intensity
c/s
100E6
80E6
60E6
40E6
20E6
0E0
299.2
301.2
655.4
656.3
200
400
600
800
1,000
m/z

Developmental Therapeutics Program
One Dose Mean Graph

**NSC:** D-810700 / 1	**Conc:** 1.00E-5 Molar	**Test Date:** Dec 10, 2018
**Experiment ID:** 1812OS42		**Report Date:** Jan 04, 2019

4f

Panel/Cell Line	Growth Percent
Leukemia	
CCRF-CEM	56.33
HL-60(TB)	94.34
K-562	51.49
MOLT-4	67.73
RPMI-8226	34.22
SR	16.62
Non-Small Cell Lung Cancer	
A549/ATCC	87.32
EKVX	90.67
HOP-62	87.41
HOP-92	84.59
NCI-H226	84.49
NCI-H23	85.40
NCI-H322M	87.07
NCI-H460	85.85
NCI-H522	78.28
Colon Cancer	
COLO 205	107.12
HCC-2998	98.53
HCT-116	7.82
HCT-15	95.02
HT29	91.70
KM12	88.83
SW-620	95.46
CNS Cancer	
SF-268	72.58
SF-295	87.58
SF-539	83.03
SNB-19	86.94
SNB-75	93.56
U251	71.20
Melanoma	
LOX IMVI	50.37
MALME-3M	102.37
M14	87.00
MDA-MB-435	86.56
SK-MEL-2	97.22
SK-MEL-28	106.69
SK-MEL-5	91.14
UACC-257	95.84
UACC-62	83.03
Ovarian Cancer	
IGROV1	74.19
OVCAR-3	95.90
OVCAR-4	91.41
OVCAR-5	99.48
OVCAR-8	78.98
NCI/ADR-RES	101.68
SK-OV-3	78.36
Renal Cancer	
786-0	76.66
A498	57.39
ACHN	93.70
CAKI-1	64.40
RXF 393	88.43
SN12C	44.91
TK-10	91.36
UO-31	65.72
Prostate Cancer	
PC-3	82.02
DU-145	78.89
Breast Cancer	
MCF7	64.78
MDA-MB-231/ATCC	80.10
HS 578T	83.58
BT-549	81.26
T-47D	80.03
MDA-MB-468	92.24
Mean	80.28
Delta	72.46
Range	99.30

Mean Growth Percent - Growth Percent

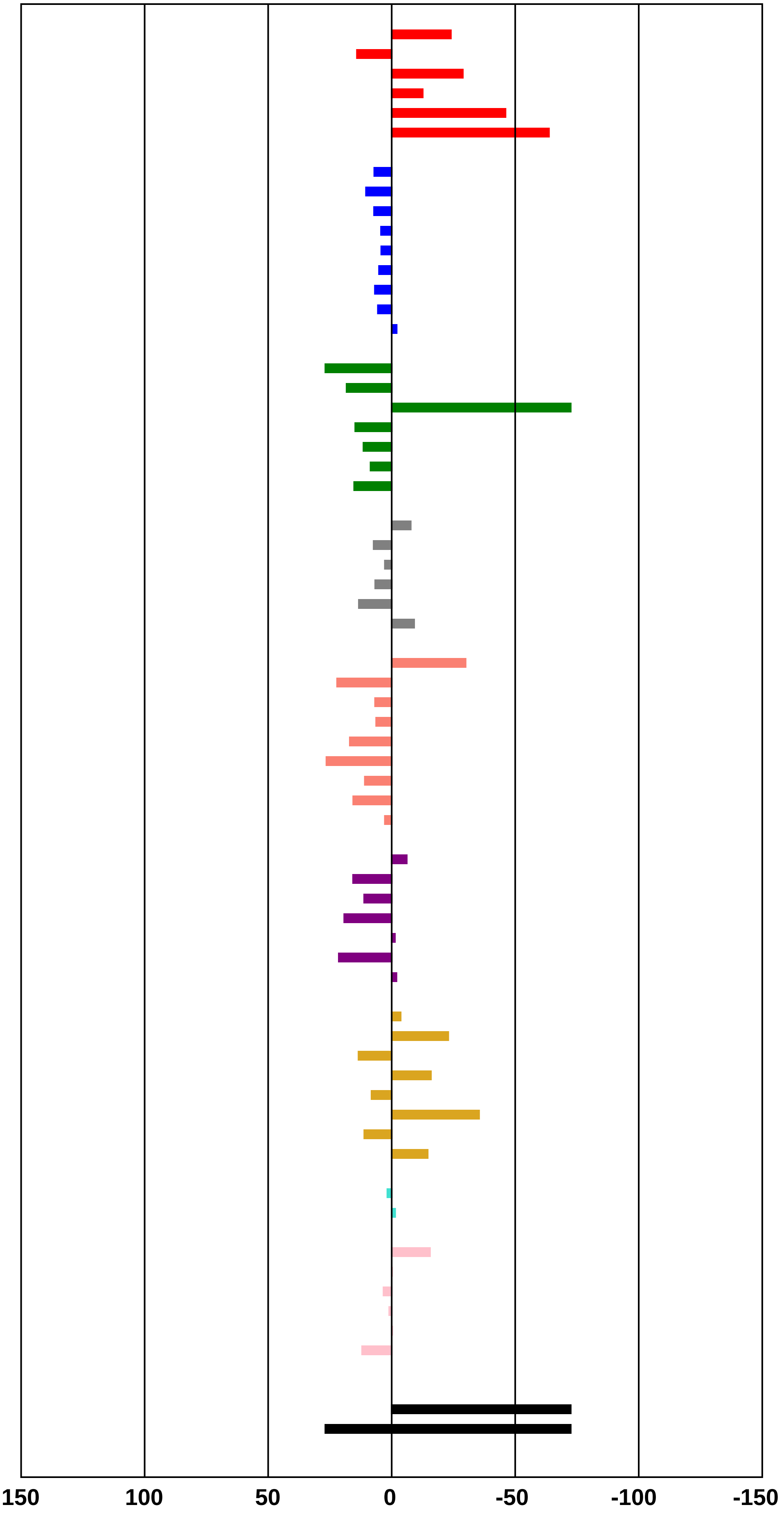

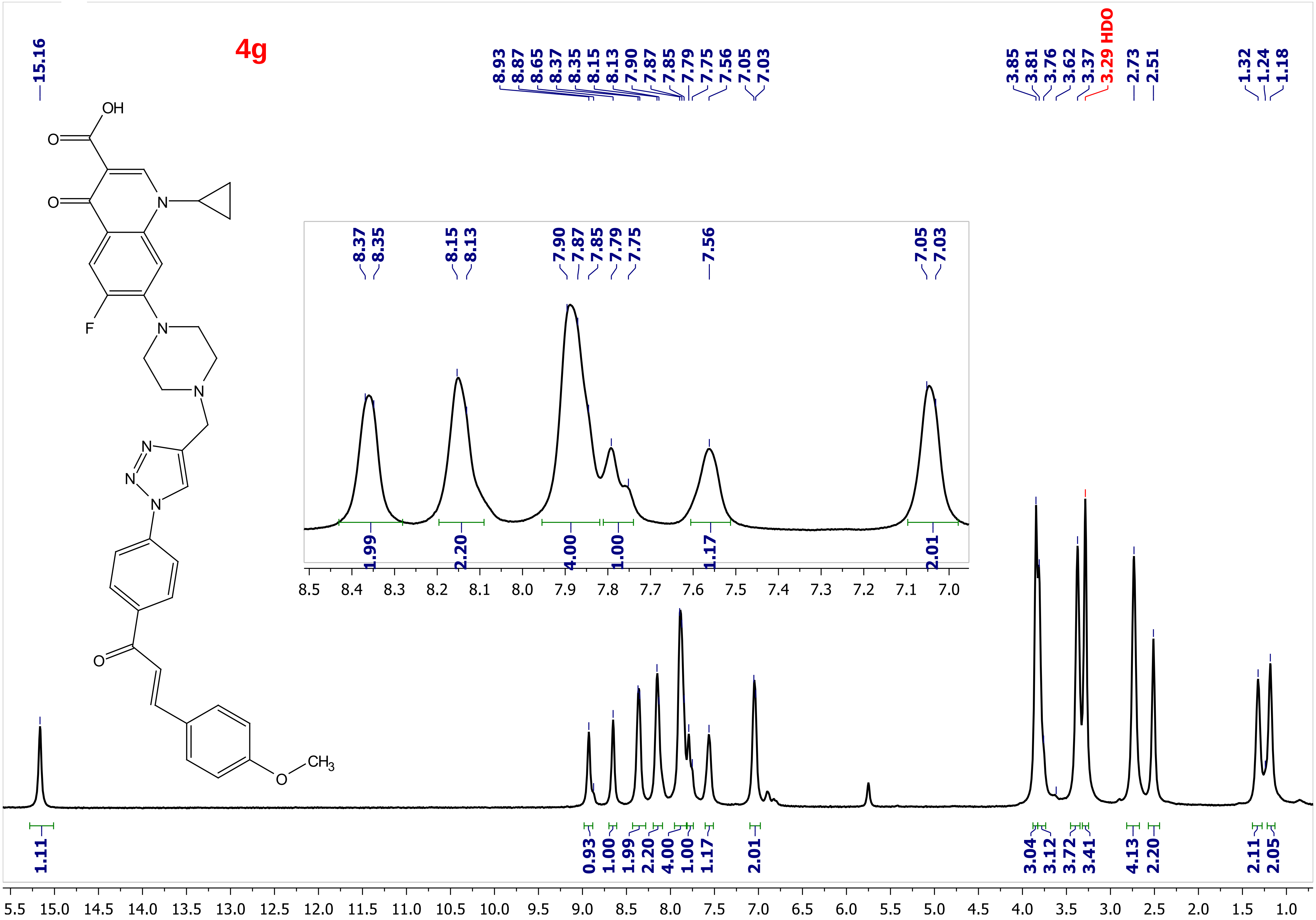
4g
15.16
8.93 8.87 8.65 8.37 8.35 8.15 8.13 7.90 7.87 7.85 7.79 7.75 7.56 7.05 7.03
3.85 3.81 3.76 3.62 3.37 3.29 HDO 2.73 2.51
1.32 1.24 1.18
OH
O
O
N
F
N
N
N
N
N
O
O
CH3
8.37 8.35 8.15 8.13 7.90 7.87 7.85 7.79 7.75 7.56 7.05 7.03
1.99 2.20 4.00 1.00 1.17 2.01
8.5 8.4 8.3 8.2 8.1 8.0 7.9 7.8 7.7 7.6 7.5 7.4 7.3 7.2 7.1 7.0
1.11 0.93 1.00 1.99 2.20 4.00 1.00 1.17 2.01 3.04 3.12 3.72 3.41 4.13 2.20 2.11 2.05
15.5 15.0 14.5 14.0 13.5 13.0 12.5 12.0 11.5 11.0 10.5 10.0 9.5 9.0 8.5 8.0 7.5 7.0 6.5 6.0 5.5 5.0 4.5 4.0 3.5 3.0 2.5 2.0 1.5 1.0

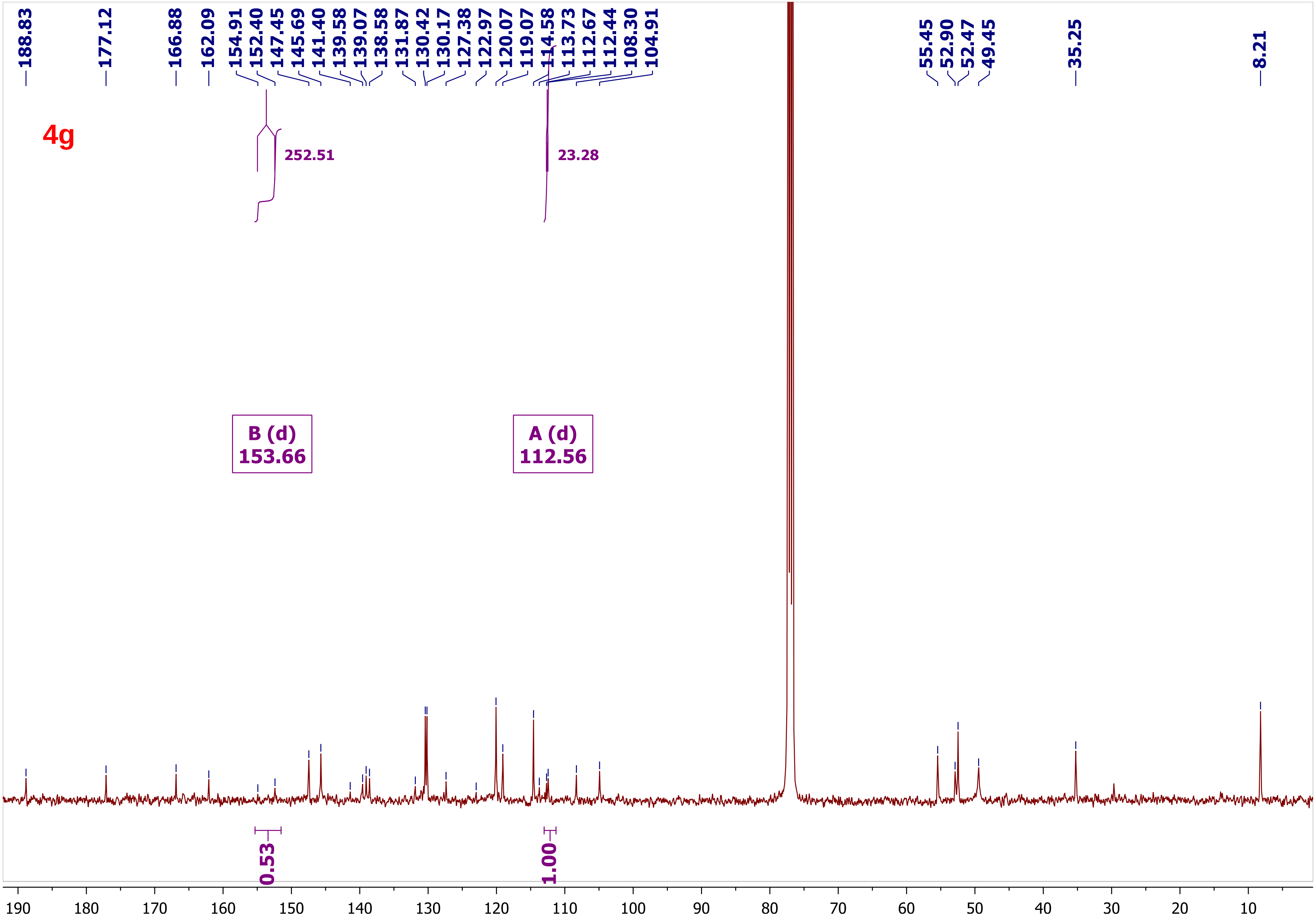
4g
188.83
177.12
166.88
162.09
154.91
152.40
147.45
145.69
141.40
139.58
139.07
138.58
131.87
130.42
130.17
127.38
122.97
120.07
119.07
114.58
113.73
112.67
112.44
108.30
104.91
55.45
52.90
52.47
49.45
35.25
8.21
252.51
23.28
B (d)
153.66
A (d)
112.56
0.53
1.00
190
180
170
160
150
140
130
120
110
100
90
80
70
60
50
40
30
20
10

4g

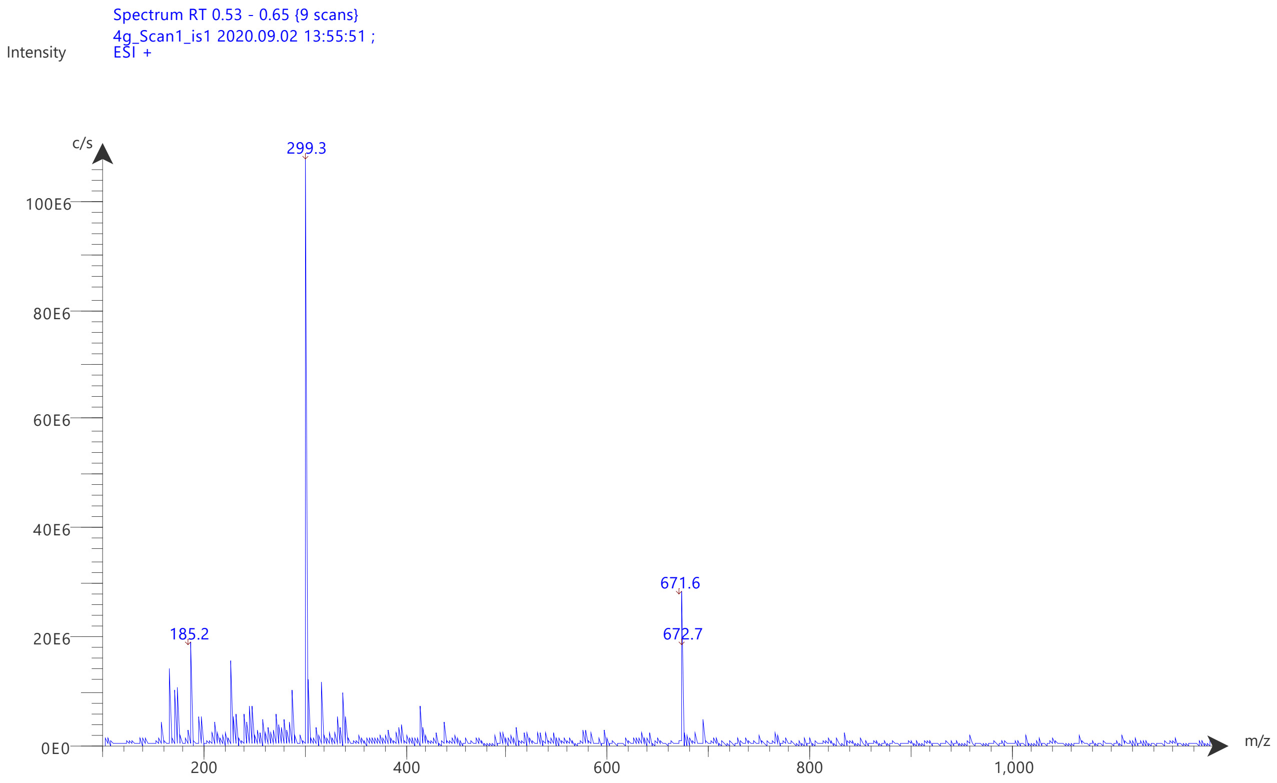
Spectrum RT 0.53 - 0.65 {9 scans}
4g_Scan1_is1 2020.09.02 13:55:51 ;
ESI +
Intensity
c/s
100E6
80E6
60E6
40E6
20E6
0E0
299.3
185.2
671.6
672.7
200
400
600
800
1,000
m/z

Developmental Therapeutics Program
One Dose Mean Graph

**NSC:** D-810696 / 1	**Conc:** 1.00E-5 Molar	**Test Date:** Dec 10, 2018
**Experiment ID:** 1812OS42		**Report Date:** Jan 04, 2019

4g

Panel/Cell Line	Growth Percent
**Leukemia**	
CCRF-CEM	74.56
HL-60(TB)	99.02
K-562	67.48
MOLT-4	81.65
RPMI-8226	11.54
SR	13.06
**Non-Small Cell Lung Cancer**	
A549/ATCC	88.99
EKVX	96.58
HOP-62	85.45
HOP-92	84.76
NCI-H226	76.34
NCI-H23	85.37
NCI-H322M	78.09
NCI-H460	82.22
NCI-H522	79.11
**Colon Cancer**	
COLO 205	97.11
HCC-2998	90.71
HCT-116	2.68
HCT-15	95.83
HT29	78.25
KM12	86.40
SW-620	81.73
**CNS Cancer**	
SF-268	77.31
SF-295	90.71
SF-539	92.76
SNB-19	84.12
SNB-75	91.63
U251	68.56
**Melanoma**	
LOX IMVI	56.86
MALME-3M	84.80
M14	88.65
MDA-MB-435	82.24
SK-MEL-2	99.09
SK-MEL-28	85.35
SK-MEL-5	81.61
UACC-257	91.74
UACC-62	74.64
**Ovarian Cancer**	
IGROV1	59.04
OVCAR-3	90.49
OVCAR-4	94.38
OVCAR-5	100.37
OVCAR-8	83.59
NCI/ADR-RES	94.75
SK-OV-3	84.63
**Renal Cancer**	
786-0	78.10
A498	60.47
ACHN	89.94
CAKI-1	75.47
RXF 393	75.01
SN12C	58.21
TK-10	92.42
UO-31	65.07
**Prostate Cancer**	
PC-3	88.96
DU-145	90.85
**Breast Cancer**	
MCF7	51.17
MDA-MB-231/ATCC	88.10
HS 578T	65.26
BT-549	90.03
T-47D	93.43
MDA-MB-468	74.40
Mean	78.85
Delta	76.17
Range	97.69

Mean Growth Percent - Growth Percent

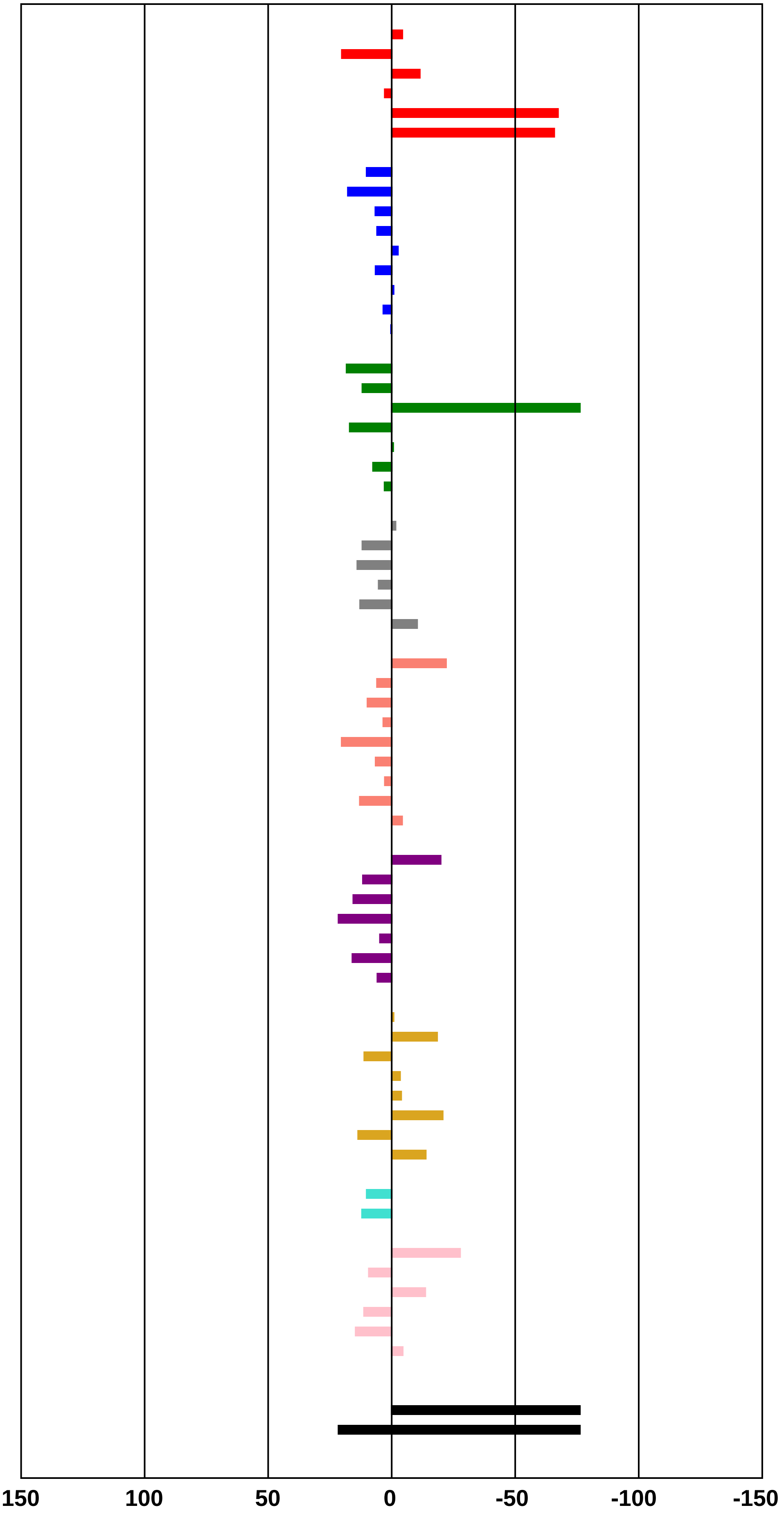

4h

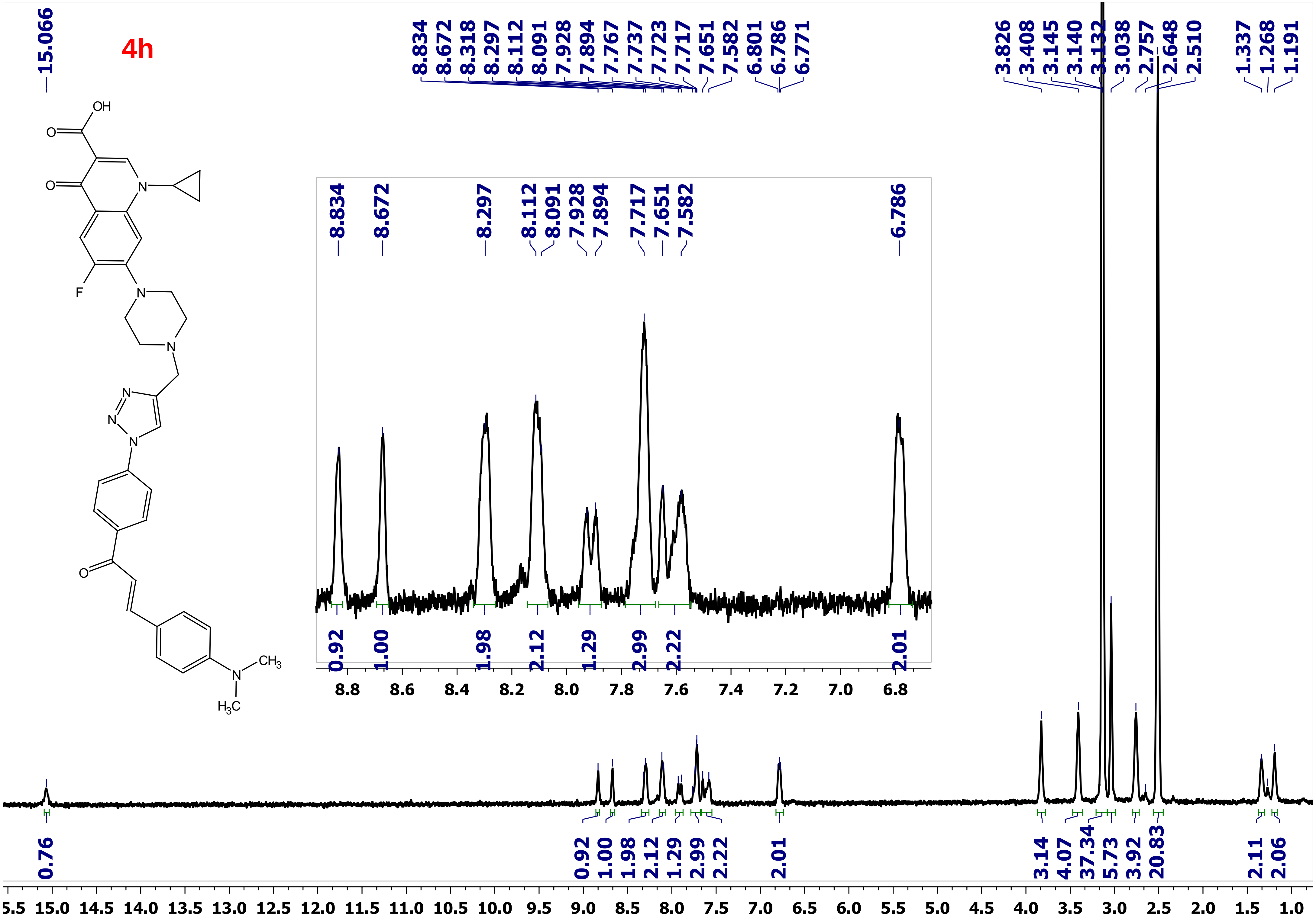

15.066
8.834
8.672
8.318
8.297
8.112
8.091
7.928
7.894
7.767
7.737
7.723
7.717
7.651
7.582
6.801
6.786
6.771
3.826
3.408
3.145
3.140
3.132
3.038
2.757
2.648
2.510
1.337
1.268
1.191
OH
O
O
N
F
N
N
N
N
N
O
N
CH3
H3C
0.92
1.00
1.98
2.12
1.29
2.99
2.22
2.01
8.8
8.6
8.4
8.2
8.0
7.8
7.6
7.4
7.2
7.0
6.8
0.76
3.14
4.07
37.34
5.73
3.92
20.83
2.11
2.06
15.5
15.0
14.5
14.0
13.5
13.0
12.5
12.0
11.5
11.0
10.5
10.0
9.5
9.0
8.5
8.0
7.5
7.0
6.5
6.0
5.5
5.0
4.5
4.0
3.5
3.0
2.5
2.0
1.5
1.0

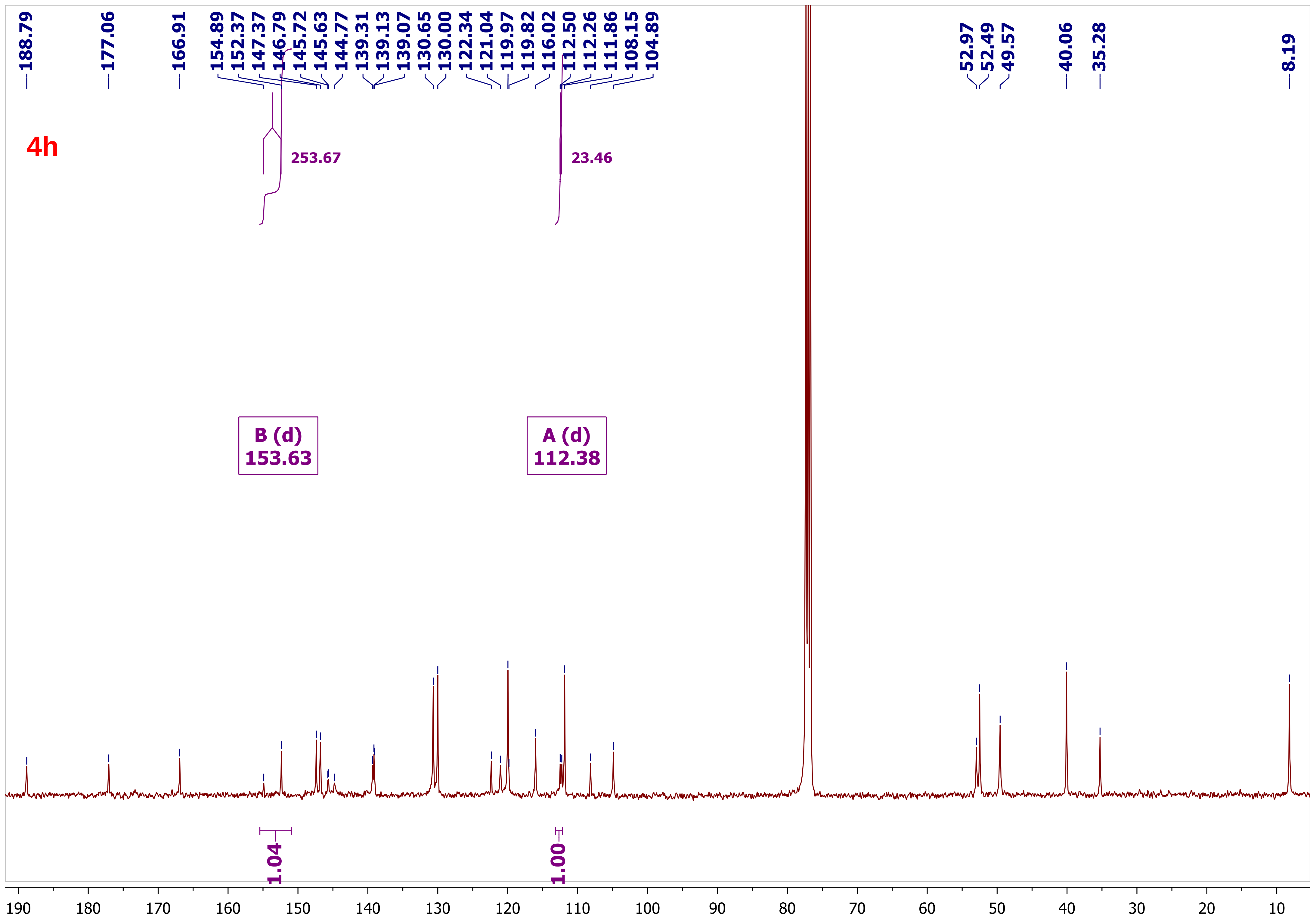
4h
188.79
177.06
166.91
154.89
152.37
147.37
146.79
145.72
145.63
144.77
139.31
139.13
139.07
130.65
130.00
122.34
121.04
119.97
119.82
116.02
112.50
112.26
111.86
108.15
104.89
52.97
52.49
49.57
40.06
35.28
8.19
253.67
23.46
B (d)
153.63
A (d)
112.38
1.04
1.00
190
180
170
160
150
140
130
120
110
100
90
80
70
60
50
40
30
20
10

4h

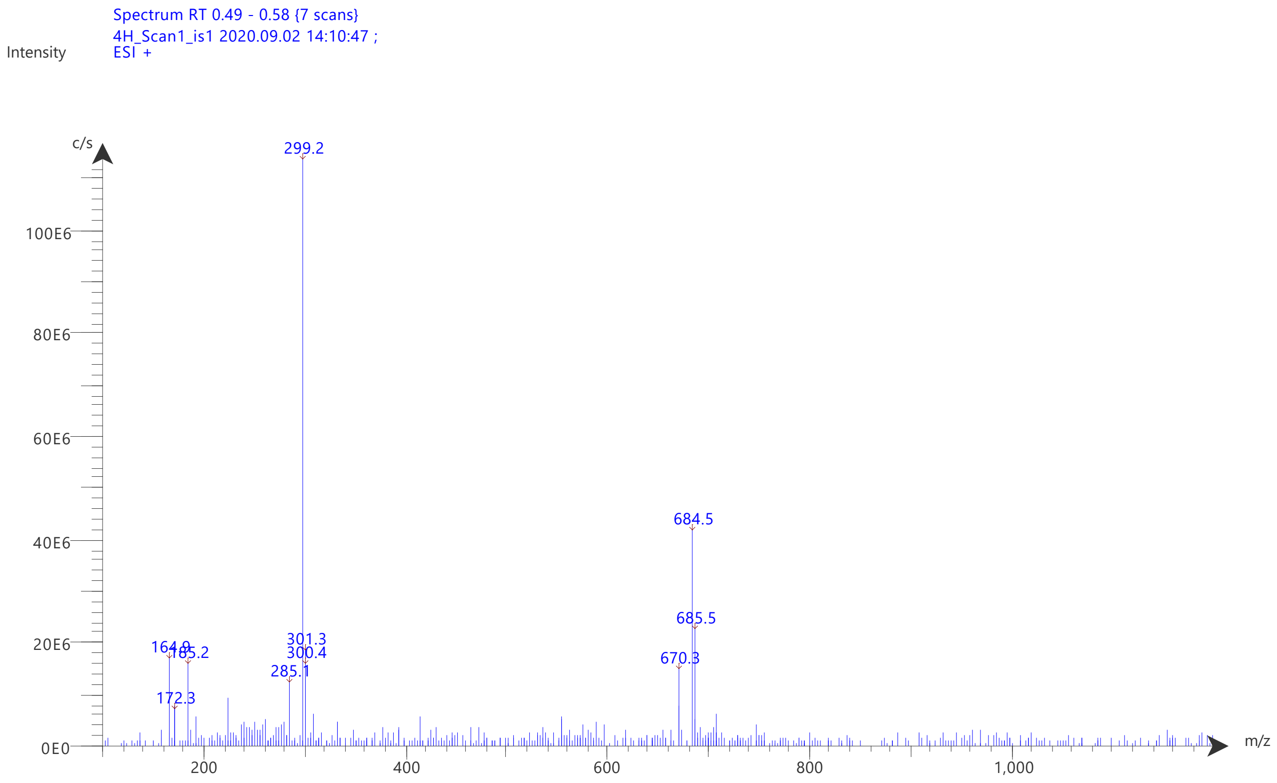
Spectrum RT 0.49 - 0.58 {7 scans}
4H_Scan1_is1 2020.09.02 14:10:47 ;
ESI +
Intensity
c/s
100E6
80E6
60E6
40E6
20E6
0E0
299.2
684.5
685.5
301.3
300.4
670.3
285.1
164.9
165.2
172.3
200
400
600
800
1,000
m/z

Developmental Therapeutics Program

One Dose Mean Graph

NSC: D-810701 / 1	Conc: 1.00E-5 Molar	Test Date: Dec 10, 2018
Experiment ID: 1812OS42		Report Date: Jan 04, 2019

4h

Panel/Cell Line	Growth Percent
Leukemia	
CCRF-CEM	84.32
HL-60(TB)	98.80
K-562	85.96
MOLT-4	85.99
RPMI-8226	44.33
SR	63.16
Non-Small Cell Lung Cancer	
A549/ATCC	93.49
EKVX	96.01
HOP-62	81.27
HOP-92	76.20
NCI-H226	84.58
NCI-H23	81.46
NCI-H322M	94.30
NCI-H460	89.65
NCI-H522	88.60
Colon Cancer	
COLO 205	101.17
HCC-2998	92.39
HCT-116	8.04
HCT-15	97.63
HT29	98.88
KM12	84.07
SW-620	98.45
CNS Cancer	
SF-268	71.51
SF-295	86.92
SF-539	73.63
SNB-19	86.43
SNB-75	70.77
U251	73.05
Melanoma	
LOX IMVI	56.93
MALME-3M	95.99
M14	87.41
MDA-MB-435	77.38
SK-MEL-2	95.46
SK-MEL-28	100.45
SK-MEL-5	86.23
UACC-257	100.56
UACC-62	77.64
Ovarian Cancer	
IGROV1	69.86
OVCAR-3	95.95
OVCAR-4	94.83
OVCAR-5	109.22
OVCAR-8	62.53
NCI/ADR-RES	102.77
SK-OV-3	84.74
Renal Cancer	
786-0	64.95
A498	64.47
ACHN	89.79
CAKI-1	69.80
RXF 393	70.94
SN12C	67.69
TK-10	104.26
UO-31	61.85
Prostate Cancer	
PC-3	89.62
DU-145	96.34
Breast Cancer	
MCF7	59.16
MDA-MB-231/ATCC	75.46
HS 578T	80.53
BT-549	73.70
T-47D	86.68
MDA-MB-468	77.12
Mean	82.02
Delta	73.98
Range	101.18

Mean Growth Percent - Growth Percent

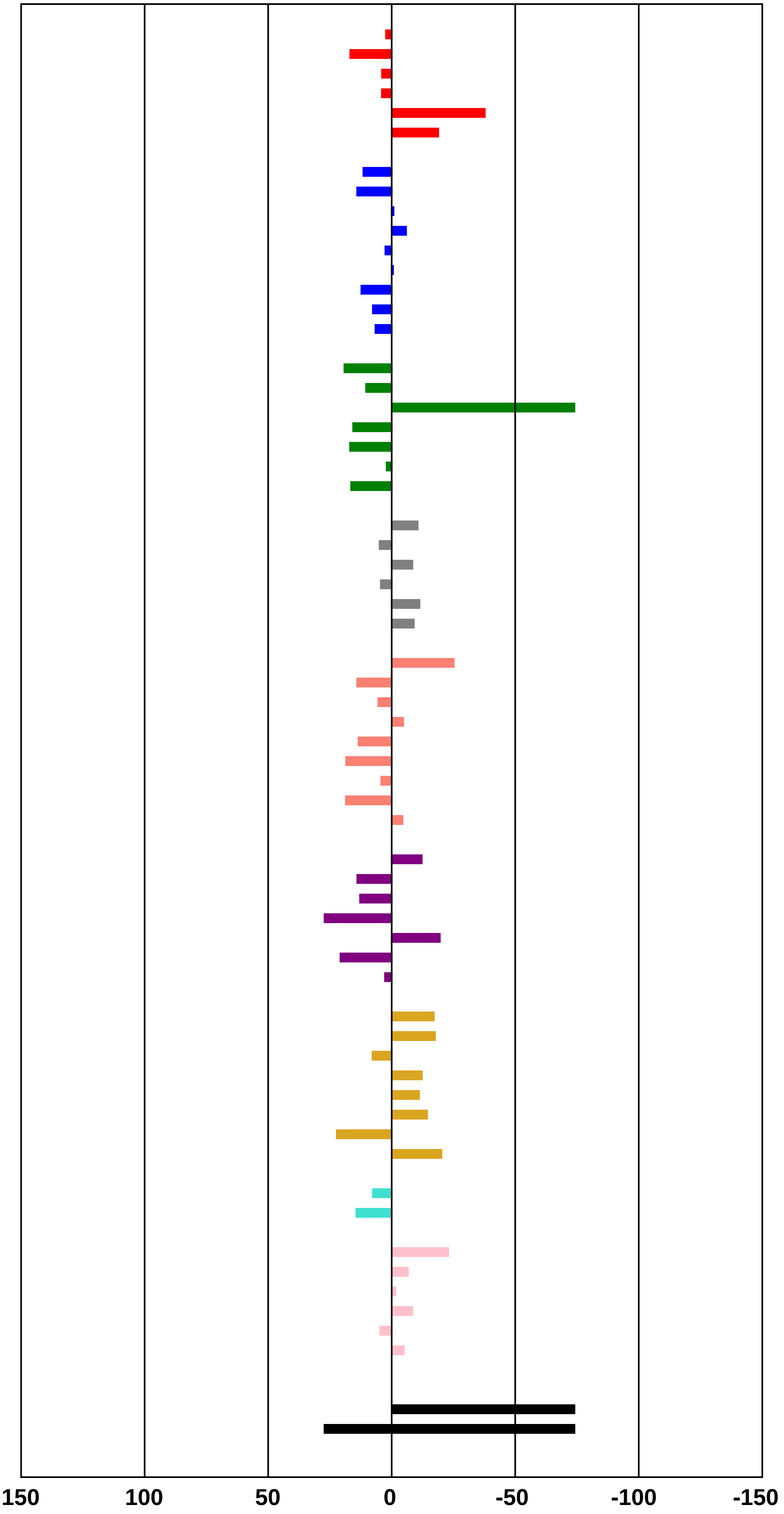

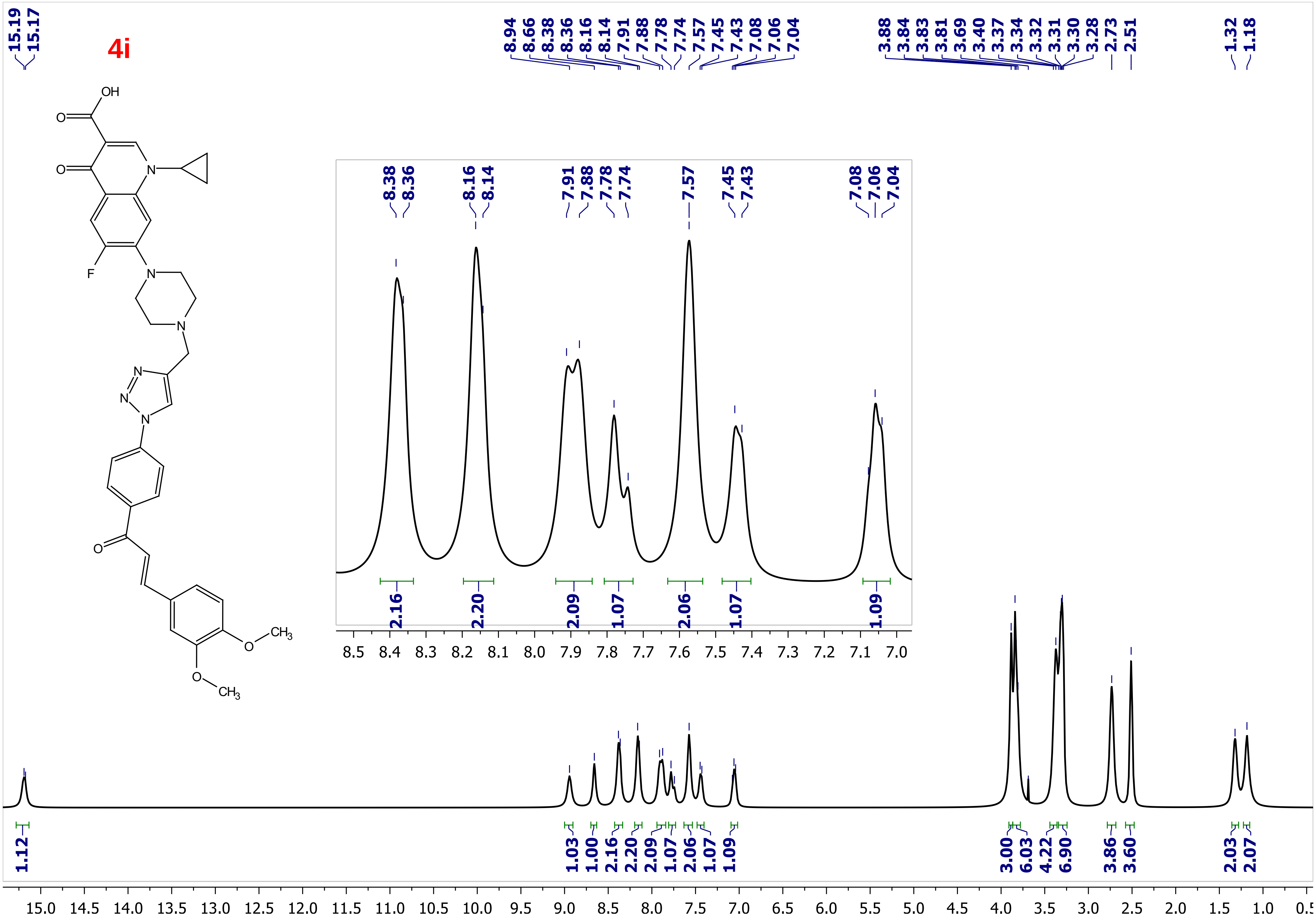
4i
15.19
15.17
8.94
8.66
8.38
8.36
8.16
8.14
7.91
7.88
7.78
7.74
7.57
7.45
7.43
7.08
7.06
7.04
3.88
3.84
3.83
3.81
3.69
3.40
3.37
3.34
3.32
3.31
3.30
3.28
2.73
2.51
1.32
1.18
2.16
2.20
2.09
1.07
2.06
1.07
1.09
1.12
1.03
1.00
3.00
6.03
4.22
6.90
3.86
3.60
2.03
2.07

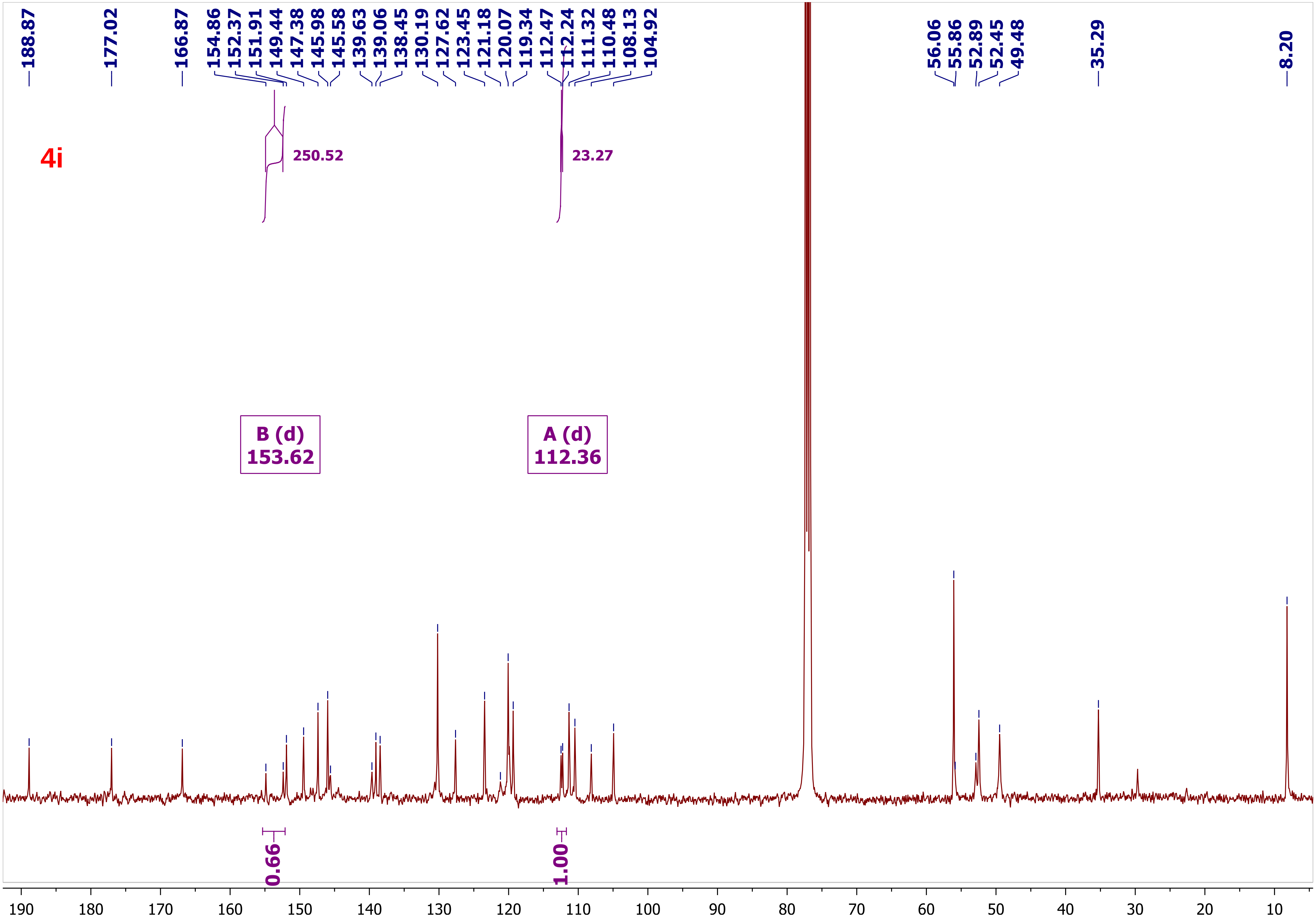

4i
188.87
177.02
166.87
154.86
152.37
151.91
149.44
147.38
145.98
145.58
139.63
139.06
138.45
130.19
127.62
123.45
121.18
120.07
119.34
112.47
112.24
111.32
110.48
108.13
104.92
56.06
55.86
52.89
52.45
49.48
35.29
8.20
250.52
23.27
B (d)
153.62
A (d)
112.36
0.66
1.00
190
180
170
160
150
140
130
120
110
100
90
80
70
60
50
40
30
20
10

4i

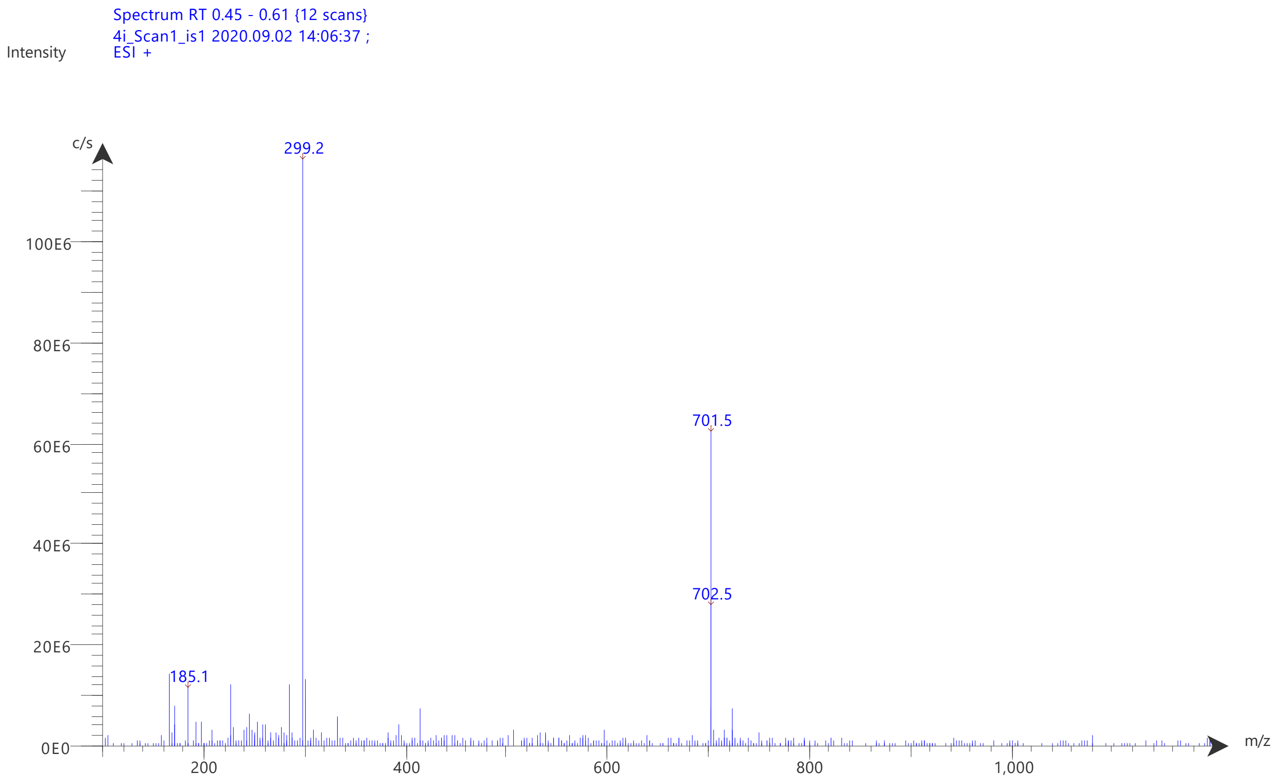
Spectrum RT 0.45 - 0.61 {12 scans}
4i_Scan1_is1 2020.09.02 14:06:37 ;
ESI +
Intensity
c/s
100E6
80E6
60E6
40E6
20E6
0E0
299.2
701.5
702.5
185.1
200
400
600
800
1,000
m/z

Developmental Therapeutics Program
One Dose Mean Graph

NSC: D-810697 / 1	Conc: 1.00E-5 Molar	Test Date: Dec 10, 2018
Experiment ID: 1812OS42		Report Date: Jan 04, 2019

Panel/Cell Line 4i	Growth Percent
**Leukemia**	
CCRF-CEM	44.02
HL-60(TB)	85.86
K-562	42.55
MOLT-4	64.18
RPMI-8226	-24.42
SR	-2.25
**Non-Small Cell Lung Cancer**	
A549/ATCC	66.12
EKVX	96.56
HOP-62	75.60
HOP-92	66.55
NCI-H226	67.29
NCI-H23	78.68
NCI-H322M	72.80
NCI-H460	77.86
NCI-H522	67.62
**Colon Cancer**	
COLO 205	98.21
HCC-2998	43.98
HCT-116	-31.29
HCT-15	80.58
HT29	29.62
KM12	42.58
SW-620	32.84
**CNS Cancer**	
SF-268	55.99
SF-295	94.87
SF-539	50.79
SNB-19	67.68
SNB-75	58.33
U251	41.44
**Melanoma**	
LOX IMVI	27.88
MALME-3M	66.19
M14	59.01
MDA-MB-435	22.17
SK-MEL-2	96.97
SK-MEL-28	79.75
SK-MEL-5	69.04
UACC-257	78.71
UACC-62	53.92
**Ovarian Cancer**	
IGROV1	26.64
OVCAR-3	39.34
OVCAR-4	88.04
OVCAR-5	92.14
OVCAR-8	67.44
NCI/ADR-RES	90.68
SK-OV-3	76.86
**Renal Cancer**	
786-0	71.90
A498	68.18
ACHN	85.01
CAKI-1	67.80
RXF 393	64.15
SN12C	32.63
TK-10	103.74
UO-31	54.92
**Prostate Cancer**	
PC-3	56.27
DU-145	83.20
**Breast Cancer**	
MCF7	13.23
MDA-MB-231/ATCC	67.15
HS 578T	52.81
BT-549	65.79
T-47D	78.64
MDA-MB-468	26.27
Mean	59.52
Delta	90.81
Range	135.03

Mean Growth Percent - Growth Percent

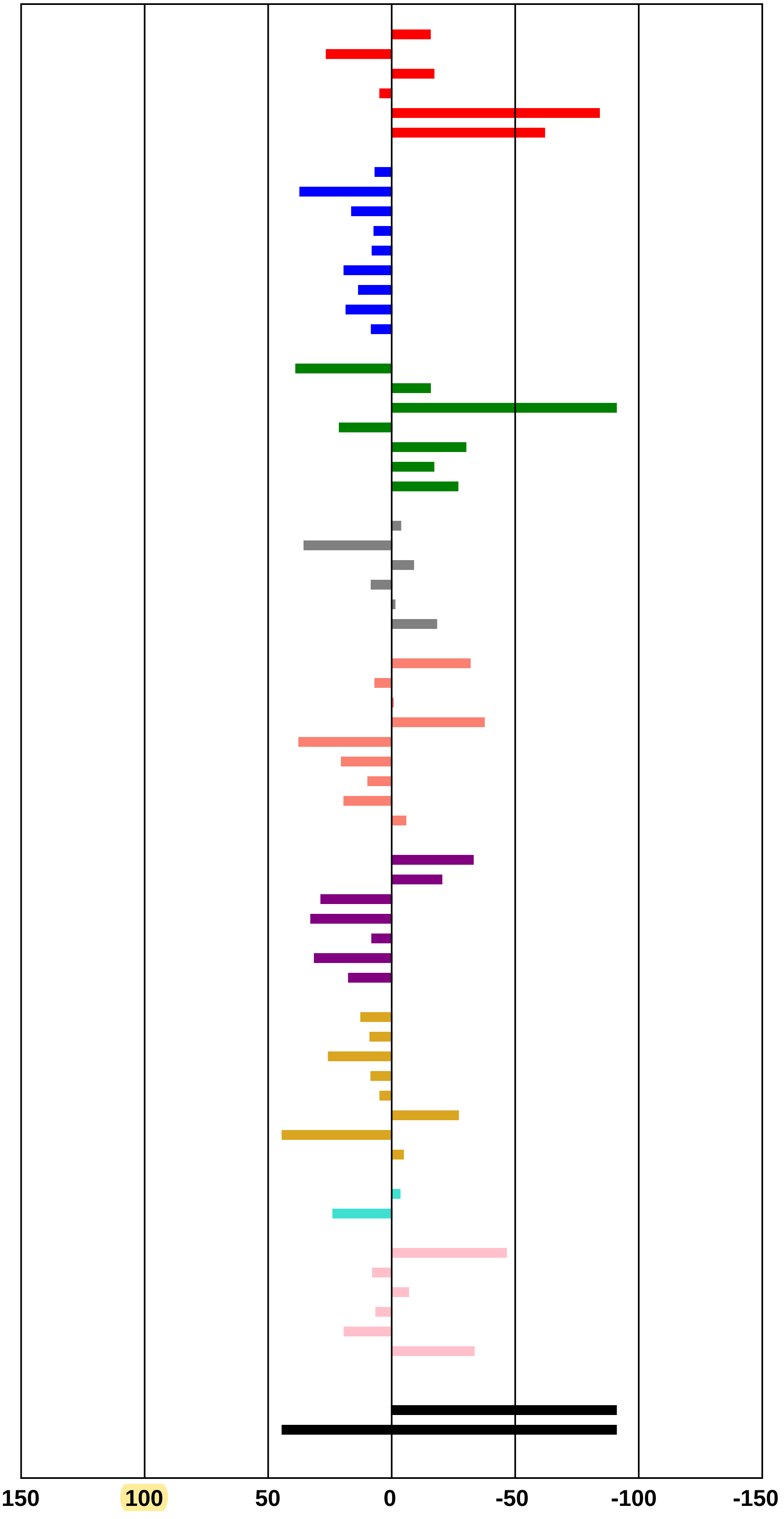

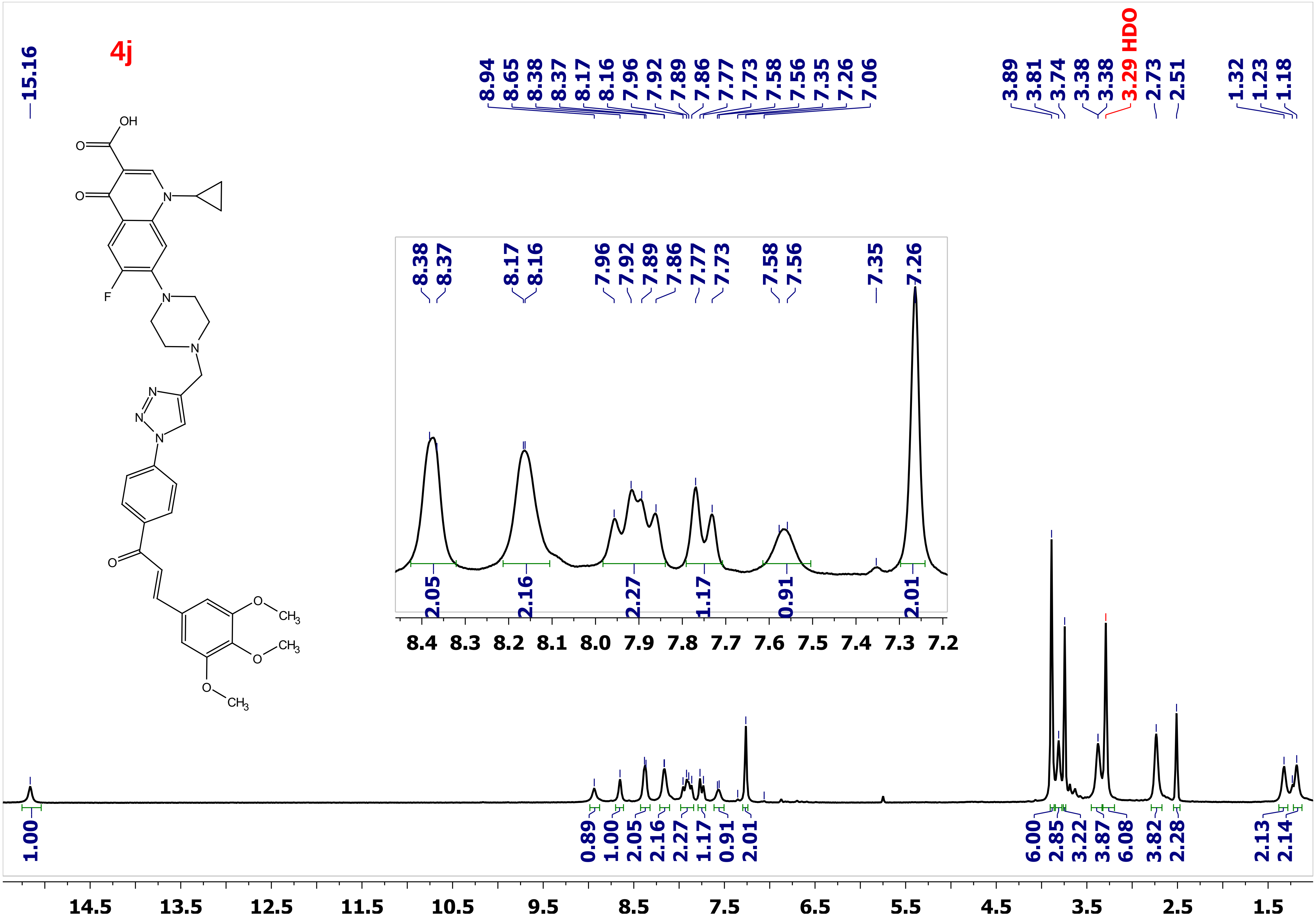
4j
15.16
8.94 8.65 8.38 8.37 8.17 8.16 7.96 7.92 7.89 7.86 7.77 7.73 7.58 7.56 7.35 7.26 7.06
3.89 3.81 3.74 3.38 3.38 3.29 HDO 2.73 2.51
1.32 1.23 1.18
OH
O
N
F
N
N
N
N
N
O
O
CH3
O
CH3
O
CH3
8.38 8.37 8.17 8.16 7.96 7.92 7.89 7.86 7.77 7.73 7.58 7.56 7.35 7.26
2.05 2.16 2.27 1.17 0.91 2.01
8.4 8.3 8.2 8.1 8.0 7.9 7.8 7.7 7.6 7.5 7.4 7.3 7.2
1.00
0.89 1.00 2.05 2.16 2.27 1.17 0.91 2.01
6.00 2.85 3.22 3.87 6.08 3.82 2.28
2.13 2.14
14.5 13.5 12.5 11.5 10.5 9.5 8.5 7.5 6.5 5.5 4.5 3.5 2.5 1.5

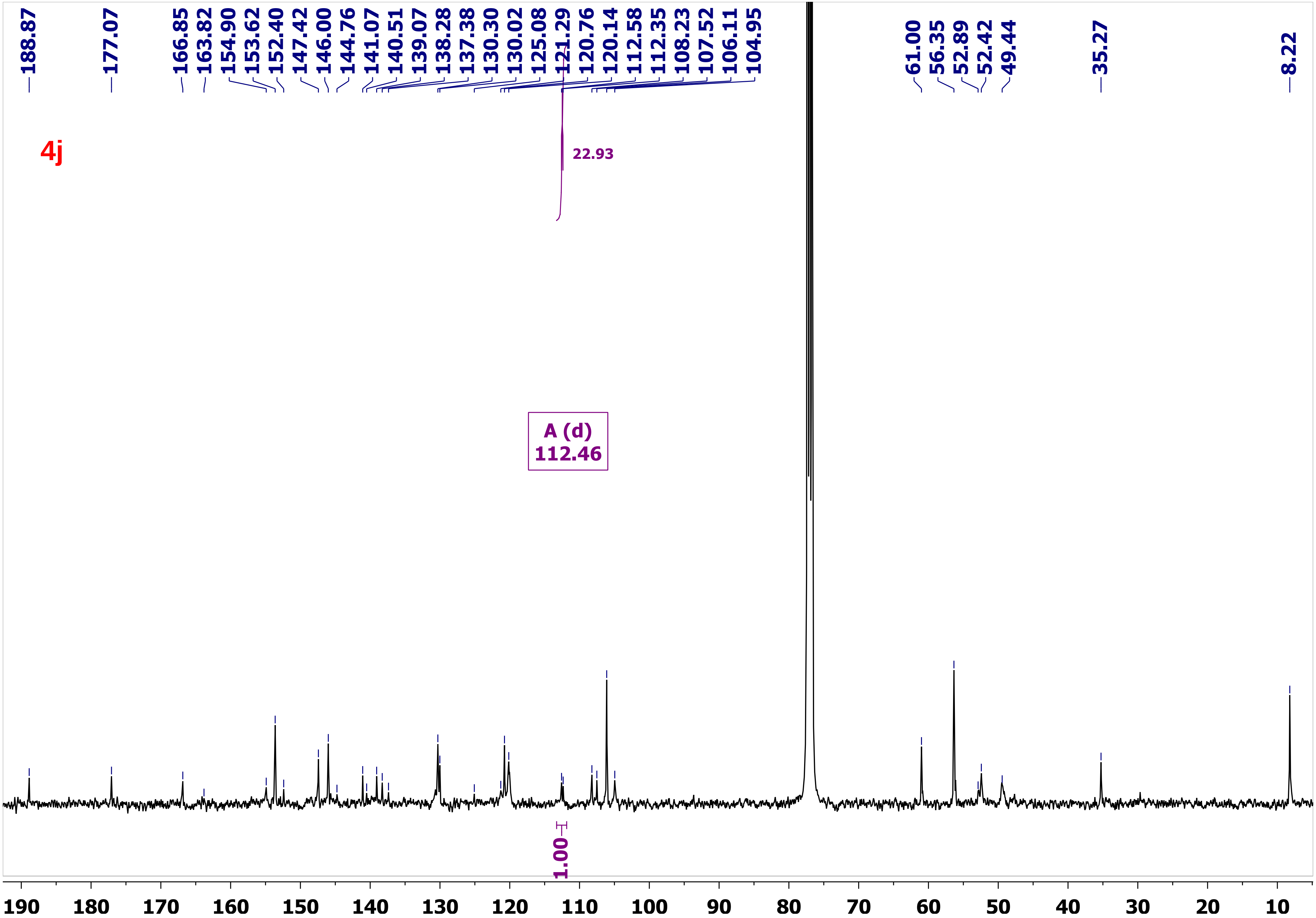
4j
188.87
177.07
166.85
163.82
154.90
153.62
152.40
147.42
146.00
144.76
141.07
140.51
139.07
138.28
137.38
130.30
130.02
125.08
121.29
120.76
120.14
112.58
112.35
108.23
107.52
106.11
104.95
61.00
56.35
52.89
52.42
49.44
35.27
8.22
22.93
A (d)
112.46
1.00
190
180
170
160
150
140
130
120
110
100
90
80
70
60
50
40
30
20
10

4j

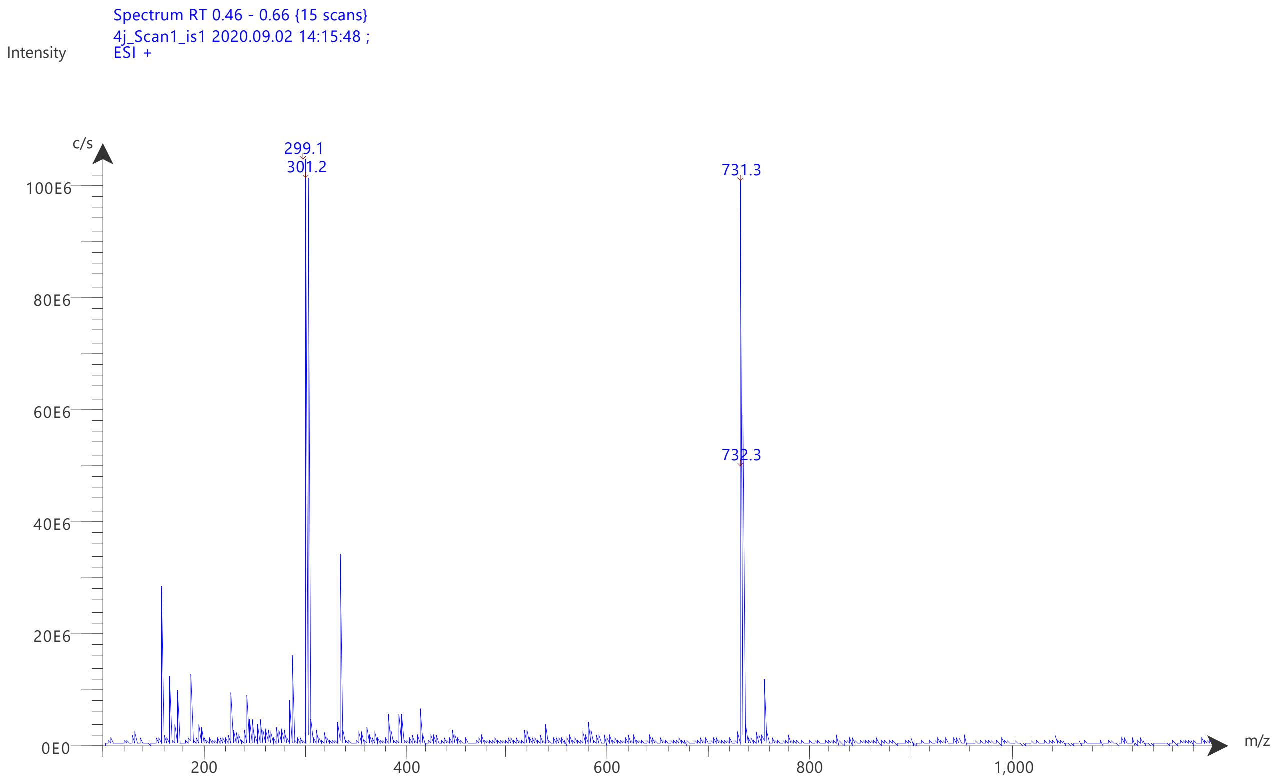
Spectrum RT 0.46 - 0.66 {15 scans}
4j_Scan1_is1 2020.09.02 14:15:48 ;
ESI +
Intensity
c/s
100E6
80E6
60E6
40E6
20E6
0E0
299.1
301.2
731.3
732.3
200
400
600
800
1,000
m/z

HPLC of 4j

Sample Purity - Detailed Report

Agilent Technologies

Peak Details (Area Percent at least 1%)

Signal Description : DAD1A,Sig=240,4 Ref=off

Spectra overlay at peak apex, up and down slopes

Peak RT : 2.004 min **Area % :** 94.39%

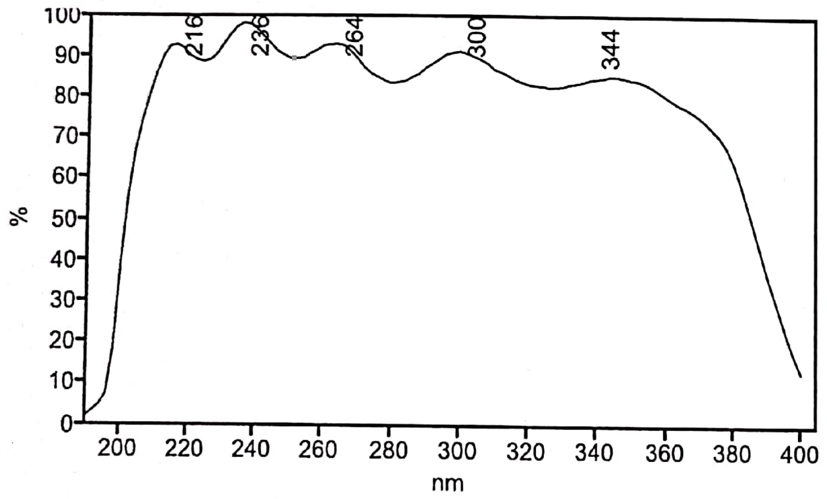

Peak RT : 2.291 min **Area % :** 4.41%

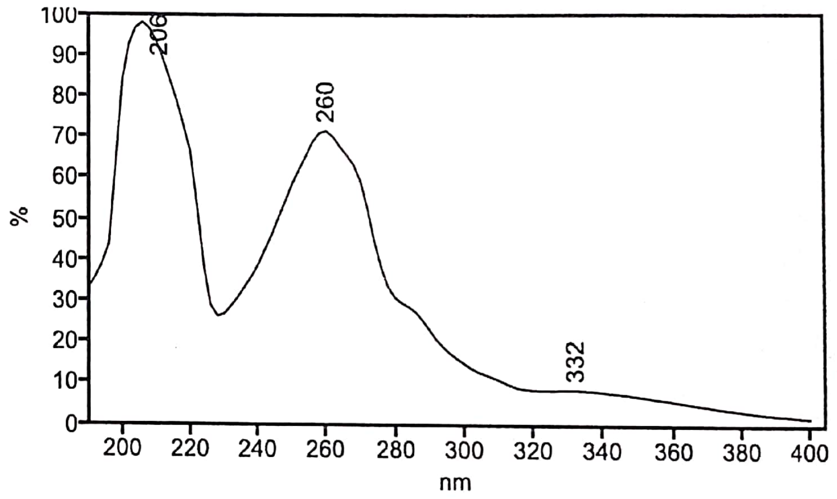

HPLC of 4j

Sample Purity - Detailed Report

Agilent Technologies

**Sequence Name:**	SingleSample
**Data file:**	Agilent HPLC-2021-03-10 11-45-37+02-00.dx
**Project Name:**	Fatma
**Sample name:**	Hamada 4j
**Operator :**	fatma
**Instrument:**	Agilent HPLC
**Acquired on:**	2021-03-10 11:47:47+02:00
**Inj. volume:**	80.000
**Location:**	P1-A2
**Acq. method:**	Hamada 10-3-21.amx
**Processing method:**	3D UV Quantitative_DefaultMethod.pmx

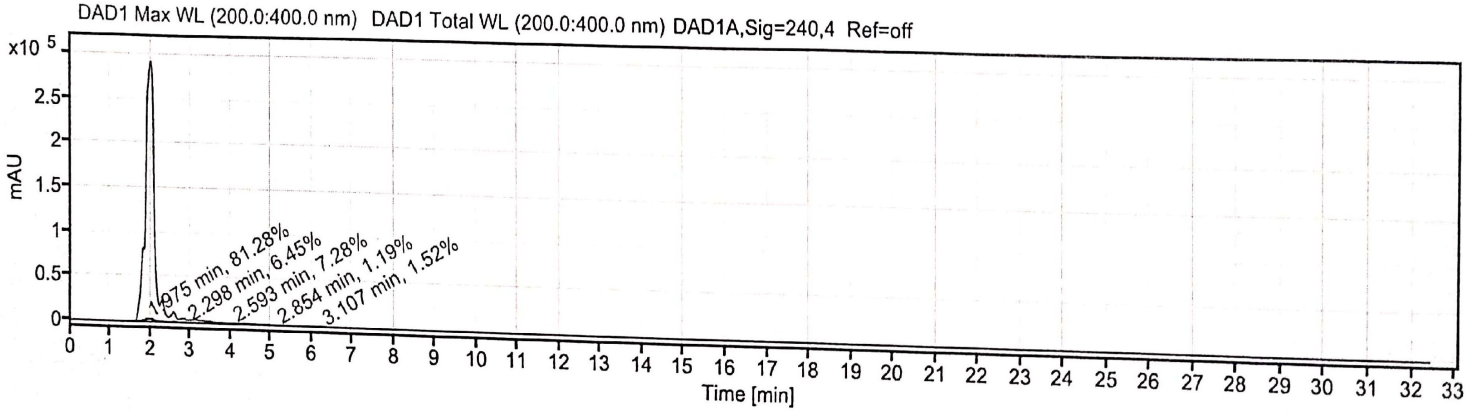

Peak Results (Area Percent at least 1%)

RT (min)	Signal Description	Width (min)	Area	Height	Area%
2.004	DAD1A,Sig=240,4 Ref=off	0.644	48027.6	3372.6	94.39
2.291	DAD1A,Sig=240,4 Ref=off	0.290	2242.4	284.1	4.41

Sample Purity Results

No "Target" had been typed in the Injection list

Developmental Therapeutics Program
One Dose Mean Graph

NSC: D-810698 / 1	Conc: 1.00E-5 Molar	Test Date: Dec 10, 2018
Experiment ID: 1812OS42		Report Date: Jan 04, 2019

4j

Panel/Cell Line	Growth Percent
**Leukemia**	
CCRF-CEM	3.05
HL-60(TB)	45.05
K-562	7.81
MOLT-4	10.52
RPMI-8226	-15.84
SR	-12.37
**Non-Small Cell Lung Cancer**	
A549/ATCC	15.71
EKVX	79.98
HOP-62	38.69
HOP-92	81.61
NCI-H226	32.41
NCI-H23	50.59
NCI-H322M	52.61
NCI-H460	53.12
NCI-H522	49.50
**Colon Cancer**	
COLO 205	60.94
HCC-2998	-39.69
HCT-116	-39.51
HCT-15	33.79
HT29	4.76
KM12	8.81
SW-620	9.41
**CNS Cancer**	
SF-268	59.55
SF-295	67.46
SF-539	7.09
SNB-19	44.71
SNB-75	65.47
U251	10.59
**Melanoma**	
LOX IMVI	-1.51
MALME-3M	57.53
M14	28.24
MDA-MB-435	5.40
SK-MEL-2	97.37
SK-MEL-28	34.56
SK-MEL-5	50.91
UACC-257	61.99
UACC-62	54.08
**Ovarian Cancer**	
IGROV1	-24.76
OVCAR-3	-16.10
OVCAR-4	73.74
OVCAR-5	72.31
OVCAR-8	39.72
NCI/ADR-RES	82.01
SK-OV-3	67.46
**Renal Cancer**	
786-0	43.65
A498	54.38
CAKI-1	59.95
RXF 393	4.51
SN12C	14.92
TK-10	79.98
UO-31	43.49
**Prostate Cancer**	
PC-3	9.95
DU-145	45.78
**Breast Cancer**	
MCF7	16.10
MDA-MB-231/ATCC	55.36
HS 578T	21.49
BT-549	50.99
T-47D	28.08
MDA-MB-468	-7.19
Mean	33.73
Delta	73.42
Range	137.06

Mean Growth Percent - Growth Percent

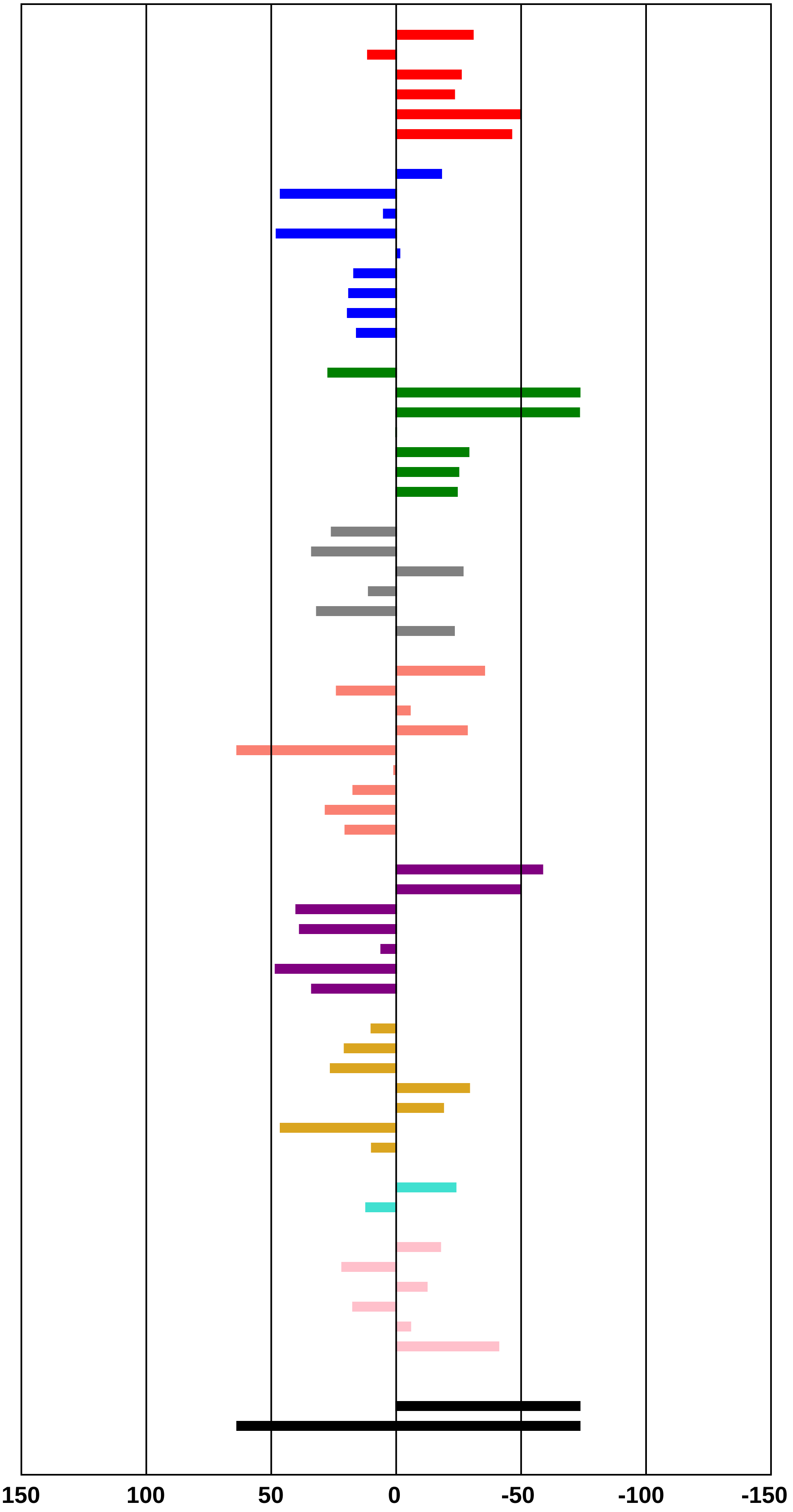

National Cancer Institute Developmental Therapeutics Program Dose Response Curves	NSC: D - 810698 / 1	SSPL: 0ZVW	EXP. ID: 1901NS56
five dose 4j	Report Date: February 18, 2019	Test Date: January 28, 2019	

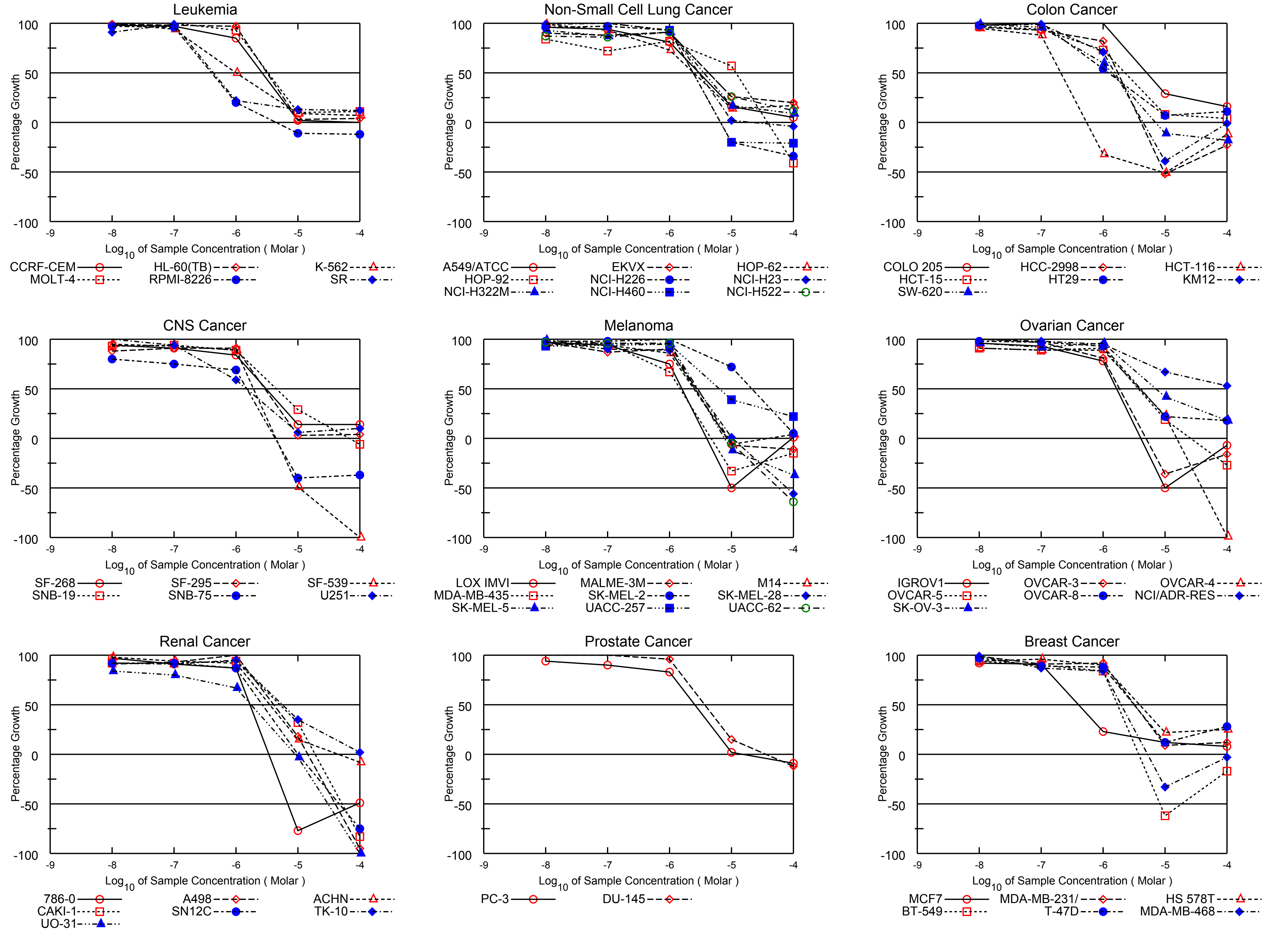

National Cancer Institute Developmental Therapeutics Program
In-Vitro Testing Results

NSC : D - 810698 / 1	Experiment ID : 1901NS56	Test Type : 08	Units : Molar
Report Date : February 18, 2019	Test Date : January 28, 2019	QNS :	MC :
COMI : NCT4	Stain Reagent : SRB Dual-Pass Related	SSPL : 0ZVW	

			Log10 Concentration												
	Time		Mean Optical Densities					Percent Growth							
Panel/Cell Line	Zero	Ctrl	-8.0	-7.0	-6.0	-5.0	-4.0	-8.0	-7.0	-6.0	-5.0	-4.0	GI50	TGI	LC50
Leukemia															
CCRF-CEM	0.580	3.110	3.071	3.029	2.726	0.628	0.582	98	97	85	2	.	2.63E-6	> 1.00E-4	> 1.00E-4
HL-60(TB)	0.877	3.308	3.282	3.265	3.239	0.955	0.974	99	98	97	3	4	3.18E-6	> 1.00E-4	> 1.00E-4
K-562	0.214	2.678	2.778	2.540	1.435	0.434	0.386	104	94	50	9	7	9.77E-7	> 1.00E-4	> 1.00E-4
MOLT-4	0.550	2.987	3.046	2.978	2.825	0.800	0.809	102	100	93	10	11	3.32E-6	> 1.00E-4	> 1.00E-4
RPMI-8226	1.128	3.138	3.080	3.057	1.527	1.007	0.992	97	96	20	-11	-12	4.01E-7	4.46E-6	> 1.00E-4
SR	0.281	1.723	1.594	1.711	0.599	0.468	0.459	91	99	22	13	12	4.34E-7	> 1.00E-4	> 1.00E-4
Non-Small Cell Lung Cancer															
A549/ATCC	0.373	2.307	2.226	2.187	1.942	0.674	0.470	96	94	81	16	5	2.98E-6	> 1.00E-4	> 1.00E-4
EKVX	0.679	1.985	1.855	1.832	1.866	1.015	0.938	90	88	91	26	20	4.24E-6	> 1.00E-4	> 1.00E-4
HOP-62	0.451	2.006	1.987	1.904	1.592	0.674	0.711	99	93	73	14	17	2.49E-6	> 1.00E-4	> 1.00E-4
HOP-92	1.205	1.998	1.872	1.777	1.854	1.656	0.715	84	72	82	57	-41	1.17E-5	3.83E-5	> 1.00E-4
NCI-H226	1.465	3.101	3.039	3.044	2.979	1.171	0.966	96	97	93	-20	-34	2.39E-6	6.63E-6	> 1.00E-4
NCI-H23	0.538	1.702	1.691	1.705	1.617	0.564	0.516	99	100	93	2	-4	2.97E-6	2.23E-5	> 1.00E-4
NCI-H322M	0.694	2.020	1.926	1.850	1.908	0.915	0.817	93	87	91	17	9	3.58E-6	> 1.00E-4	> 1.00E-4
NCI-H460	0.284	3.025	3.104	3.130	2.827	0.228	0.226	103	104	93	-20	-21	2.40E-6	6.66E-6	> 1.00E-4
NCI-H522	0.911	2.813	2.569	2.551	2.642	1.402	1.144	87	86	91	26	12	4.25E-6	> 1.00E-4	> 1.00E-4
Colon Cancer															
COLO 205	0.443	2.016	1.989	2.042	2.025	0.899	0.692	98	102	101	29	16	5.08E-6	> 1.00E-4	> 1.00E-4
HCC-2998	0.652	2.522	2.447	2.391	2.185	0.311	0.500	96	93	82	-52	-23	1.73E-6	4.08E-6	.
HCT-116	0.242	2.582	2.475	2.293	0.165	0.120	0.214	95	88	-32	-51	-12	2.06E-7	5.40E-7	.
HCT-15	0.355	2.616	2.555	2.470	2.008	0.528	0.449	97	94	73	8	4	2.25E-6	> 1.00E-4	> 1.00E-4
HT29	0.240	2.144	2.108	2.171	1.275	0.378	0.448	98	101	54	7	11	1.24E-6	> 1.00E-4	> 1.00E-4
KM12	0.467	2.980	2.934	2.950	2.262	0.287	0.461	98	99	71	-39	-1	1.57E-6	4.46E-6	> 1.00E-4
SW-620	0.200	1.533	1.514	1.482	0.994	0.178	0.164	99	96	60	-11	-18	1.37E-6	6.98E-6	> 1.00E-4
CNS Cancer															
SF-268	0.759	2.576	2.467	2.405	2.294	1.015	1.019	94	91	84	14	14	3.09E-6	> 1.00E-4	> 1.00E-4
SF-295	0.484	1.746	1.597	1.637	1.629	0.522	0.537	88	91	91	3	4	2.91E-6	> 1.00E-4	> 1.00E-4
SF-539	0.884	2.840	2.741	2.704	2.617	0.448	-0.049	95	93	89	-49	-100	1.91E-6	4.39E-6	1.03E-5
SNB-19	0.749	2.687	2.549	2.578	2.476	1.320	0.702	93	94	89	29	-6	4.52E-6	6.67E-5	> 1.00E-4
SNB-75	0.890	1.792	1.610	1.566	1.510	0.535	0.557	80	75	69	-40	-37	1.49E-6	4.29E-6	> 1.00E-4
U251	0.256	1.738	1.749	1.654	1.129	0.352	0.401	101	94	59	6	10	1.48E-6	> 1.00E-4	> 1.00E-4
Melanoma															
LOX IMVI	0.406	3.043	2.952	2.894	2.371	0.203	0.421	97	94	75	-50	1	1.57E-6	.	> 1.00E-4
MALME-3M	0.679	1.759	1.726	1.614	1.656	0.629	0.606	97	87	90	-7	-11	2.59E-6	8.41E-6	> 1.00E-4
M14	0.388	1.827	1.766	1.743	1.632	0.366	0.441	96	94	86	-6	4	2.49E-6	.	> 1.00E-4
MDA-MB-435	0.443	2.281	2.163	2.202	1.684	0.295	0.378	94	96	67	-33	-15	1.49E-6	4.67E-6	> 1.00E-4
SK-MEL-2	1.315	3.004	2.974	2.971	3.013	2.527	1.392	98	98	101	72	5	2.11E-5	> 1.00E-4	> 1.00E-4
SK-MEL-28	0.607	2.348	2.317	2.174	2.157	0.622	0.268	98	90	89	1	-56	2.77E-6	1.04E-5	7.89E-5
SK-MEL-5	0.752	3.208	3.179	3.107	3.090	0.663	0.477	99	96	95	-12	-37	2.64E-6	7.74E-6	> 1.00E-4
UACC-257	1.097	2.653	2.539	2.567	2.580	1.701	1.443	93	94	95	39	22	6.34E-6	> 1.00E-4	> 1.00E-4
UACC-62	1.057	3.020	2.952	2.950	2.938	1.000	0.381	97	96	96	-5	-64	2.83E-6	8.84E-6	5.77E-5
Ovarian Cancer															
IGROV1	0.358	1.916	1.852	1.801	1.567	0.178	0.332	96	93	78	-50	-7	1.64E-6	4.04E-6	.
OVCAR-3	0.547	2.169	2.184	2.128	1.859	0.351	0.459	101	97	81	-36	-16	1.84E-6	4.93E-6	> 1.00E-4
OVCAR-4	0.555	1.333	1.266	1.244	1.250	0.736	0.004	91	89	89	23	-99	3.94E-6	1.55E-5	3.96E-5
OVCAR-5	0.528	1.778	1.661	1.638	1.661	0.767	0.383	91	89	91	19	-27	3.70E-6	2.57E-5	> 1.00E-4
OVCAR-8	0.418	2.365	2.329	2.301	2.238	0.839	0.774	98	97	93	22	18	4.03E-6	> 1.00E-4	> 1.00E-4
NCI/ADR-RES	0.621	2.279	2.279	2.250	2.192	1.734	1.508	100	98	95	67	53	> 1.00E-4	> 1.00E-4	> 1.00E-4
SK-OV-3	0.927	2.341	2.281	2.225	2.280	1.521	1.186	96	92	96	42	18	7.09E-6	> 1.00E-4	> 1.00E-4
Renal Cancer															
786-0	0.623	2.647	2.578	2.466	2.376	0.144	0.319	97	91	87	-77	-49	1.67E-6	3.39E-6	.
A498	1.828	2.859	2.770	2.784	2.887	2.013	0.087	91	93	103	18	-95	4.18E-6	1.44E-5	3.98E-5
ACHN	0.320	1.787	1.757	1.702	1.675	0.533	0.296	98	94	92	15	-8	3.50E-6	4.52E-5	> 1.00E-4
CAKI-1	0.613	2.558	2.403	2.404	2.437	1.234	0.105	92	92	94	32	-83	5.10E-6	1.90E-5	5.17E-5
SN12C	0.664	2.704	2.547	2.549	2.436	0.666	0.164	92	92	87	.	-75	2.66E-6	1.00E-5	4.62E-5
TK-10	0.733	2.318	2.190	2.173	2.239	1.287	0.773	92	91	95	35	2	5.61E-6	> 1.00E-4	> 1.00E-4
UO-31	0.630	2.080	1.843	1.785	1.596	0.610	-0.077	84	80	67	-3	-100	1.73E-6	9.01E-6	3.05E-5
Prostate Cancer															
PC-3	0.511	2.432	2.314	2.250	2.097	0.552	0.466	94	90	83	2	-9	2.54E-6	1.57E-5	> 1.00E-4
DU-145	0.346	1.777	1.819	1.794	1.716	0.557	0.303	103	101	96	15	-12	3.67E-6	3.49E-5	> 1.00E-4
Breast Cancer															
MCF7	0.309	1.876	1.754	1.730	0.664	0.491	0.429	92	91	23	12	8	3.96E-7	> 1.00E-4	> 1.00E-4
MDA-MB-231/ATCC	0.656	1.686	1.671	1.592	1.606	0.746	0.783	99	91	92	9	12	3.20E-6	> 1.00E-4	> 1.00E-4
HS 578T	0.915	2.023	1.962	1.982	1.915	1.163	1.197	94	96	90	22	25	3.91E-6	> 1.00E-4	> 1.00E-4
BT-549	1.174	2.532	2.447	2.394	2.317	0.443	0.975	94	90	84	-62	-17	1.71E-6	3.76E-6	.
T-47D	1.075	2.782	2.727	2.595	2.580	1.275	1.552	97	89	88	12	28	3.16E-6	> 1.00E-4	> 1.00E-4
MDA-MB-468	0.915	1.611	1.601	1.523	1.501	0.614	0.885	99	87	84	-33	-3	1.96E-6	5.24E-6	> 1.00E-4

National Cancer Institute Developmental Therapeutics Program
Mean Graphs

NSC : D - 810698/1 | Units :Molar | SSPL :0ZVW | EXP. ID :1901NS56

Report Date :February 18, 2019 | Test Date :January 28, 2019

Panel/Cell Line	Log_{10} GI50	Log_{10} TGI	Log_{10} LC50
**Leukemia**			
CCRF-CEM	-5.58	> -4.00	> -4.00
HL-60(TB)	-5.50	> -4.00	> -4.00
K-562	-6.01	> -4.00	> -4.00
MOLT-4	-5.48	> -4.00	> -4.00
RPMI-8226	-6.40	-5.35	> -4.00
SR	-6.36	> -4.00	> -4.00
**Non-Small Cell Lung Cancer**			
A549/ATCC	-5.53	> -4.00	> -4.00
EKVX	-5.37	> -4.00	> -4.00
HOP-62	-5.60	> -4.00	> -4.00
HOP-92	-4.93	-4.42	> -4.00
NCI-H226	-5.62	-5.18	> -4.00
NCI-H23	-5.53	-4.65	> -4.00
NCI-H322M	-5.45	> -4.00	> -4.00
NCI-H460	-5.62	-5.18	> -4.00
NCI-H522	-5.37	> -4.00	> -4.00
**Colon Cancer**			
COLO 205	-5.29	> -4.00	> -4.00
HCC-2998	-5.76	-5.39	
HCT-116	-6.69	-6.27	
HCT-15	-5.65	> -4.00	> -4.00
HT29	-5.91	> -4.00	> -4.00
KM12	-5.81	-5.35	> -4.00
SW-620	-5.86	-5.16	> -4.00
**CNS Cancer**			
SF-268	-5.51	> -4.00	> -4.00
SF-295	-5.54	> -4.00	> -4.00
SF-539	-5.72	-5.36	-4.99
SNB-19	-5.34	-4.18	> -4.00
SNB-75	-5.83	-5.37	> -4.00
U251	-5.83	> -4.00	> -4.00
**Melanoma**			
LOX IMVI	-5.80		> -4.00
MALME-3M	-5.59	-5.08	> -4.00
M14	-5.60		> -4.00
MDA-MB-435	-5.83	-5.33	> -4.00
SK-MEL-2	-4.68	> -4.00	> -4.00
SK-MEL-28	-5.56	-4.98	-4.10
SK-MEL-5	-5.58	-5.11	> -4.00
UACC-257	-5.20	> -4.00	> -4.00
UACC-62	-5.55	-5.05	-4.24
**Ovarian Cancer**			
IGROV1	-5.78	-5.39	
OVCAR-3	-5.74	-5.31	> -4.00
OVCAR-4	-5.40	-4.81	-4.40
OVCAR-5	-5.43	-4.59	> -4.00
OVCAR-8	-5.40	> -4.00	> -4.00
NCI/ADR-RES	> -4.00	> -4.00	> -4.00
SK-OV-3	-5.15	> -4.00	> -4.00
**Renal Cancer**			
786-0	-5.78	-5.47	
A498	-5.38	-4.84	-4.40
ACHN	-5.46	-4.35	> -4.00
CAKI-1	-5.29	-4.72	-4.29
SN12C	-5.58	-5.00	-4.34
TK-10	-5.25	> -4.00	> -4.00
UO-31	-5.76	-5.05	-4.52
**Prostate Cancer**			
PC-3	-5.60	-4.80	> -4.00
DU-145	-5.44	-4.46	> -4.00
**Breast Cancer**			
MCF7	-6.40	> -4.00	> -4.00
MDA-MB-231/ATCC	-5.49	> -4.00	> -4.00
HS 578T	-5.41	> -4.00	> -4.00
BT-549	-5.77	-5.43	
T-47D	-5.50	> -4.00	> -4.00
MDA-MB-468	-5.71	-5.28	> -4.00
_MID	-5.58	-4.58	-4.06
Delta	1.11	1.69	0.93
Range	2.69	2.27	0.99

National Cancer Institute Developmental Therapeutics Program Dose Response Curves	NSC : D - 810698/1	SSPL:0ZVW	EXP. ID:1901NS56
	Report Date:February 18, 2019	Test Date:January 28, 2019	

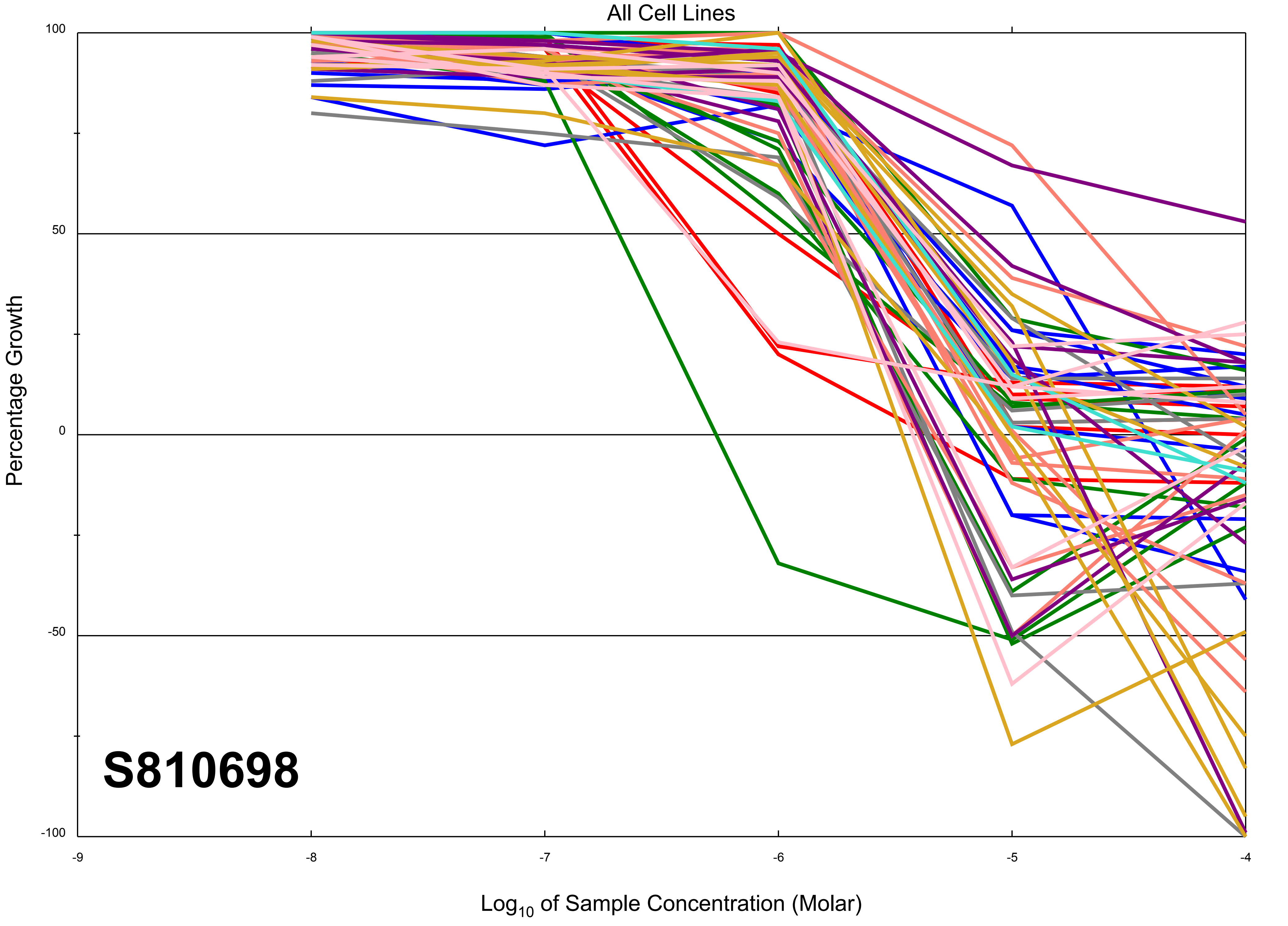

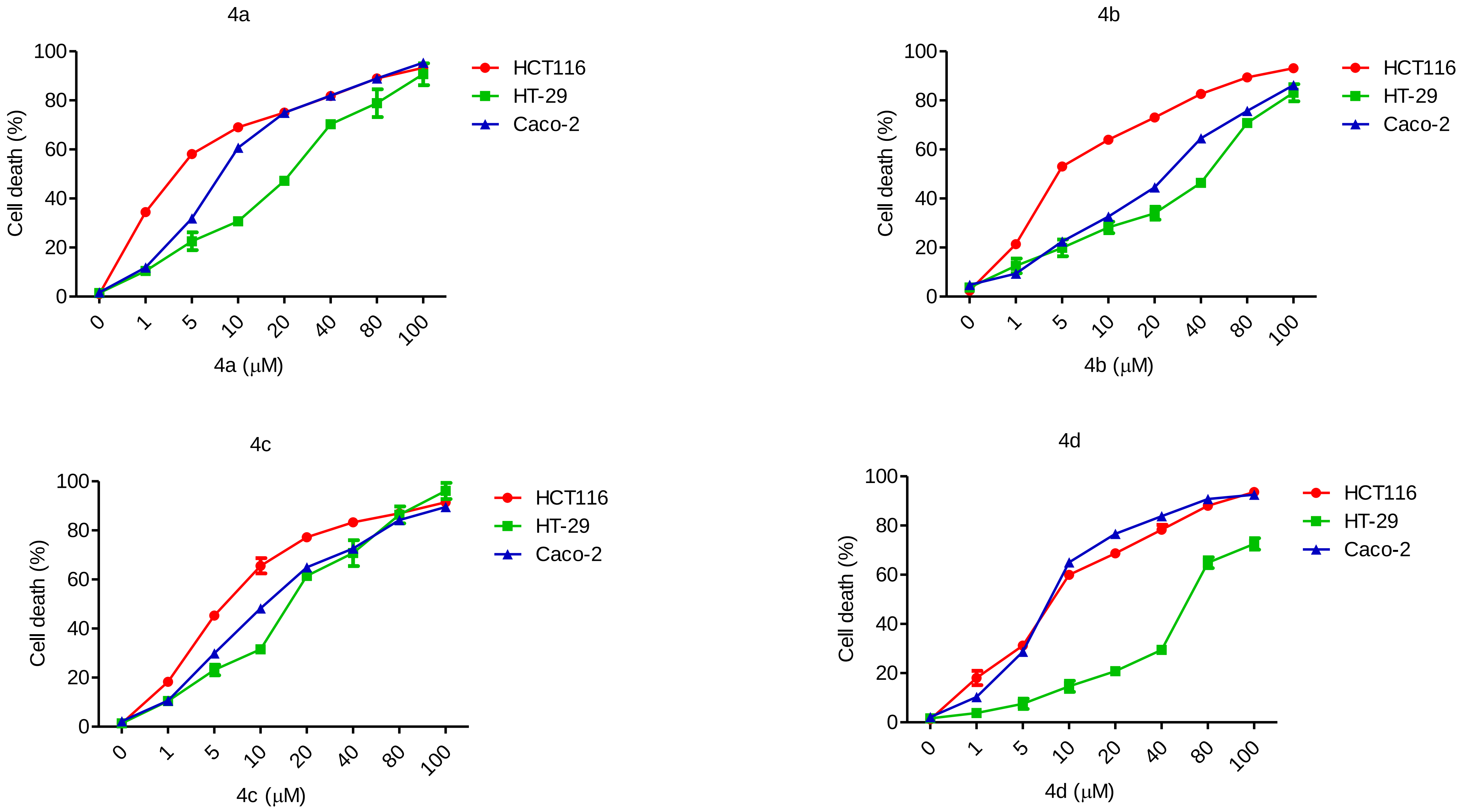
4a
HCT116
HT-29
Caco-2
Cell death (%)
4a (μM)
4b
4b (μM)
4c
4c (μM)
4d
4d (μM)
0
1
5
10
20
40
80
100

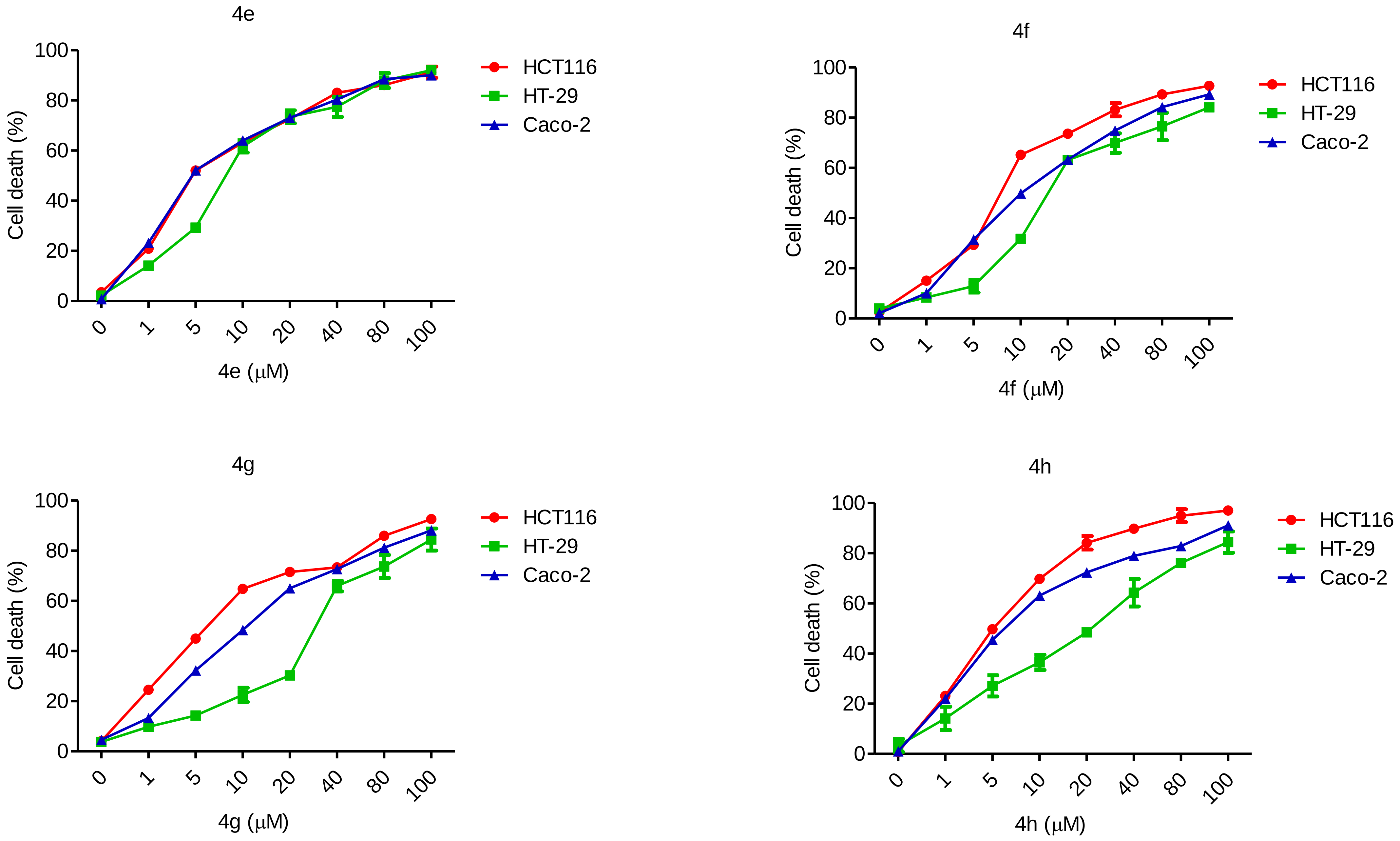
4e
Cell death (%)
0
20
40
60
80
100
0
1
5
10
20
40
80
100
4e (μM)
HCT116
HT-29
Caco-2
4f
Cell death (%)
4f (μM)
HCT116
HT-29
Caco-2
4g
Cell death (%)
4g (μM)
HCT116
HT-29
Caco-2
4h
Cell death (%)
4h (μM)
HCT116
HT-29
Caco-2

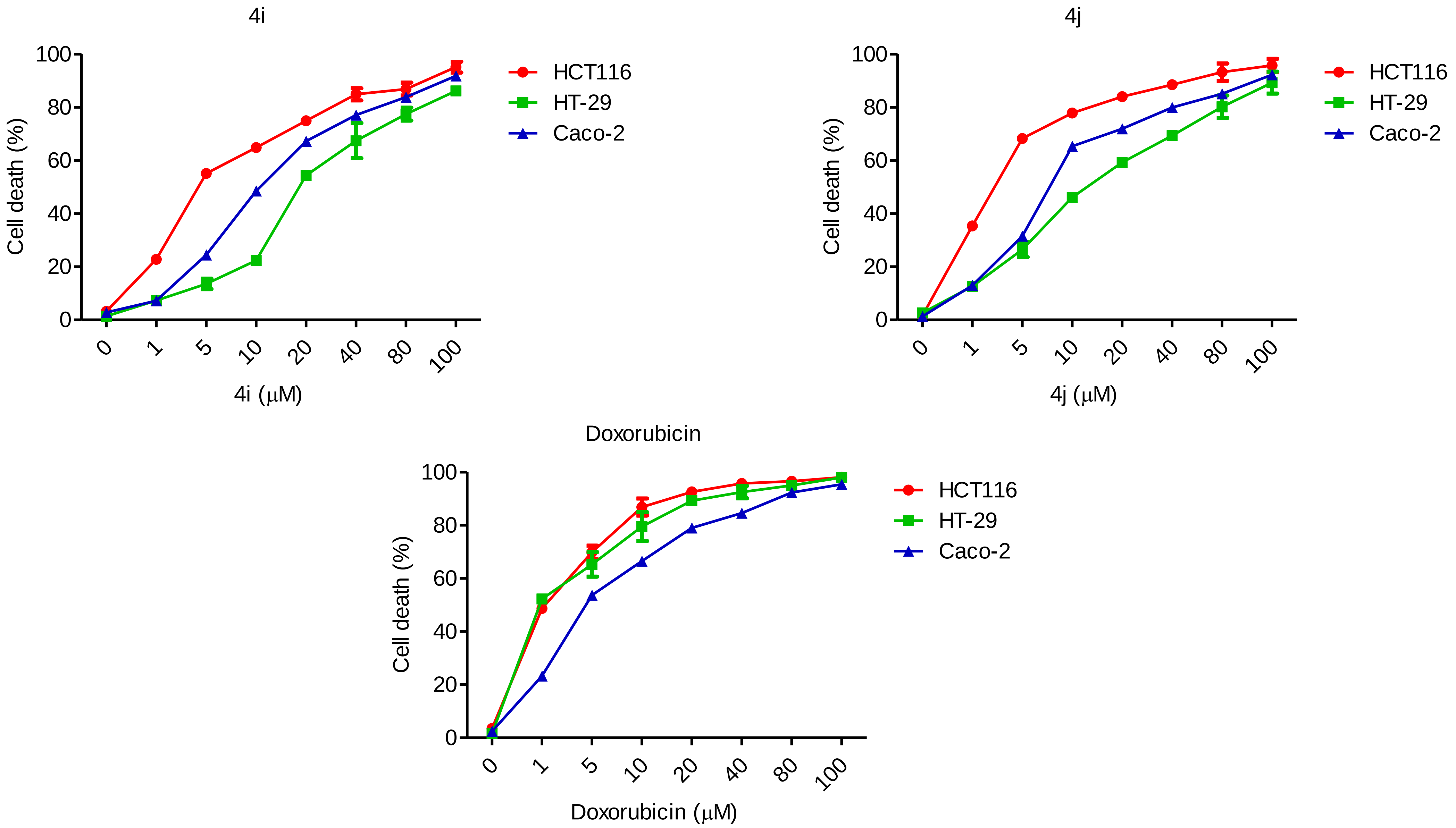
4i
Cell death (%)
4i (μM)
HCT116
HT-29
Caco-2
4j
Cell death (%)
4j (μM)
HCT116
HT-29
Caco-2
Doxorubicin
Cell death (%)
Doxorubicin (μM)
HCT116
HT-29
Caco-2

Table 1: Anticancer screening data at single dose assay (10^{-5} M) as percent cell growth inhibition of ciprofloxacin hybrids **4a-j**

**Cell line**	**% of Growth Inhibition**									
	**4a**	**4b**	**4c**	**4d**	**4e**	**4f**	**4g**	**4h**	**4i**	**4j**
**Leukemia**										
CCRF-CEM	--[a]	70.13	35.09	65.35	79.98	43.67	--	--	55.98	96.95
HL-60(TB)	--	--	--	--	--	--	--	--	--	54.95
K-562	--	59.47	44.05	39.3	78.07	48.51	32.52	--	57.45	92.19
MOLT-4	--	31.81	--	--	68.16	32.27	--	--	35.82	89.48
RPMI-8226	52.13	114.59	100.51	93.98	124.65	65.78	88.46	55.67	124.42	115.84
SR	--	85.88	75.95	78.73	92.89	83.38	86.94	36.84	102.25	112.37
**Non-Small Cell Lung Cancer**										
A549/ATCC	--	--	--	--	--	--	--	--	33.88	84.29
EKVX	--	--	--	--	--	--	--	--	--	--
HOP-62	--	32.35	--	--	32.74	--	--	--	--	61.31
HOP-92	--	--	--	--	49.74	--	--	--	33.45	--
NCI-H226	30.36	--	--	--	--	--	--	--	32.71	67.59
NCI-H23	--	--	--	--	--	--	--	--	--	49.41
NCI-H322M	--	--	--	--	--	--	--	--	--	47.39
NCI-H460	--	33.35	--	--	67.53	--	--	--	--	46.88
NCI-H522	--	--	--	--	51.49	--	--	--	32.38	50.5
**Colon Cancer**										
COLO 205	--	--	--	--	--	--	--	--	--	39.06
HCC-2998	--	40.69	--	--	--	--	--	--	56.02	139.69
HCT-116	109.74	153.36	93.05	82.69	110.26	92.18	97.32	91.96	131.29	139.51
HCT-15	--	--	--	--	36.56	--	--	--	--	66.21
HT29	--	46.69	--	--	71.98	--	--	--	70.38	95.24
KM12	--	70.09	--	--	67.11	--	--	--	57.42	91.19

SW-620	--	--	--	--	56.76	--	--	--	67.16	90.59
**CNS Cancer**										
SF-260	--	43.63	--	--	64.31	--	--	--	44.01	40.45
SF-295	--		--	--	--	--	--	--		32.54
SF-539	--	39.88	31.03	--	72.05	--	--	--	49.21	92.91
SNB-19	--	46.16	--	--	68.41	--	--	--	32.32	55.29
SNB-75	--	--	--	--	31.91	--	--	--	41.67	34.53
U251	--	65.68	57.85	31.56	83.45	--	31.44	--	58.56	89.41
**Melanoma**										
LOX IMVI	44.65	63.08	63.76	44.28	79.66	49.63	43.14	43.07	72.12	101.51
MALME-3M	--	--	--	--	--	--	--	--	33.81	42.47
M14	--	--	--	--	39.13	--	--	--	40.99	71.76
MDA-MB-435	--	--	--	--	35.34	--	--	--	77.83	--
SK-MEL-2	--	--	--	--		--	--	--	--	--
SK-MEL-28	--	--	--	--	32.01	--	--	--	--	65.44
SK-MEL-5	--	--	--	--	--	--	--	--	30.96	49.09
UACC-257	--	--	--	--	--	--	--	--	--	38.01
UACC-62	--	38.84	--	--	44.2	--	--	--	46.08	45.92
**Ovarian Cancer**										
IGROV1	--	48.16	--	--	60.44	--	40.96	30.14	73.36	124.76
OVCAR-3	--	42.66	--	--	53.37	--	--	--	60.66	116.1
OVCAR-4	--	37.12	--	--	--	--	--	--	--	--
OVCAR-5	--	--	--	--	--	--	--	--	--	--
OVCAR-8	--	--	37.96	--	76.66	--	--	37.47	32.56	60.28
NCI/ADR-RES	--	--	--	--	--	--	--	--	--	--
SK-OV-3	--	--	--	--	--	--	--	--	--	--
**Renal Cancer**										
786-0	--	46.1	45.82		66.75	42.61	--	35.05	--	56.35

[a] No inhibition at concentration of 10^{-5} M

A498	--	--	47.82	65.1	38.86	--	39.53	35.53	31.82	45.62
ACHN 1	--	--	--	--	--	35.6	--	--	--	100
CAKI-1	--	47.46	38.6	--	63.14	--	--	30.2	32.2	40.05
RXF 393	--	--	41.7	44.31	55.03				35.85	95.49
SN12C	--	62.37	54.19	41.16	91.23	55.09	41.79	32.31	67.37	85.08
TK-10	--	--	--	--	--	--	--	--	--	--
UO-31	36.86	47.36	37.53	34.6	37.21	34.28	34.93	38.15	45.08	56.51
**Breast Cancer**										
MCF7	--	62.38	41.82	38.57	75.77	35.22	48.83	40.84	86.77	83.9
MDA-MB231/ATCC	--	--	--	33.63	34.72	--	--	--	32.85	44.64
HS 578T	--		32.6	--	42.55	--	34.74	--	47.19	78.51
BT-549	--	--	--	--	68.01	--	--	--	34.21	49.01
T-47D	--	--	--	--	--	--	--	--	--	71.92
MDA-MB-468	--	35.23	33.96	--	67.13	--	--	--	73.73	107.19
MCF7		--	--	--	--	--	--	--	--	--
**Prostate Cancer**										
PC-3 71.03	--	--	--	--	42.62	--	--	--	--	90.05
DU-145	--	--	--	--	48.35	--	--	--	--	54.22

Docking Studies

Table 2: Molecular modeling data for compounds **4a, 4b, 4e, 4i, 4j**, and camptothecin in Topo I (PDB: ID **1T8I**)

Compound	Energy score (Kcal/mol)	H-bond forming residues & types	Function group forming H-bond & bond length (Å)	Other interactions residues & types	Function group of interaction & bond length (Å)
**Camptothecin**	-9.761	Asp 533 (H-donor)<br>Lys 532 (H- acceptor)<br>Arg 364 (H- acceptor)	-OH (2.47 Å)<br>-CO (2.63 Å)<br>-N of pyridine (3.04 Å)	TGP 11 (pi-pi)<br>DA 113 (pi-pi)<br>DA 113 (pi-pi)<br>DC 112 (pi-pi)	Pyridine ring (3.61 Å)<br>Pyridine ring (3.95 Å)<br>Phenyl ring (3.60Å)<br>Phenyl ring (3.72 Å)
**4a**	-10.905	Trp 416 (H- acceptor)	-CO of pyridone (3.03Å)	Lys 425 (H-pi)<br>DA 113 (pi-pi)<br>TGP 11 (pi-pi)<br>DT 10 (pi-pi)	pyridone ring (4.02Å)<br>triazole ring (3.98Å)<br>triazole ring (3.83Å)<br>phenyl ring (4.00 Å)
**4b**	-11.622			TGP 11 (pi-pi)	triazole ring (3.41Å)
**4e**	-11.154	Arg 488 (H- acceptor)<br>His 632 ((H- acceptor)	-CO of pyridone (3.30Å)<br>-CO of COOH (3.36 Å)	Trp 416 (pi-H)<br>DT 10 (H-pi)<br>DG 12 (pi-H)	Phenyl ring (4.53 Å)<br>piperazine ring (3.37 Å)<br>pyridone ring (4.51Å)
**4i**	-11.738	Arg 364 (H- acceptor)<br>Asp 533 (H-donor)	-N2 of triazole ring (3.37 Å)<br>-Piperazine ring (3.21 Å)	DT 10 (H-pi)<br>DC 112 (H-pi)<br>DA 113 (pi-pi)<br>TGP 11 (pi-pi)	piperazine ring (4.18 Å)<br>CH of chalcone (4.44 Å)<br>triazole ring (3.67Å)<br>triazole ring (3.71Å)
**4j**	-13.267			Glu 356 (pi-H)<br>DC 112 (pi-pi)	Trimethoxy Phenyl ring (3.84 Å)<br>Phenyl ring (3.98 Å)

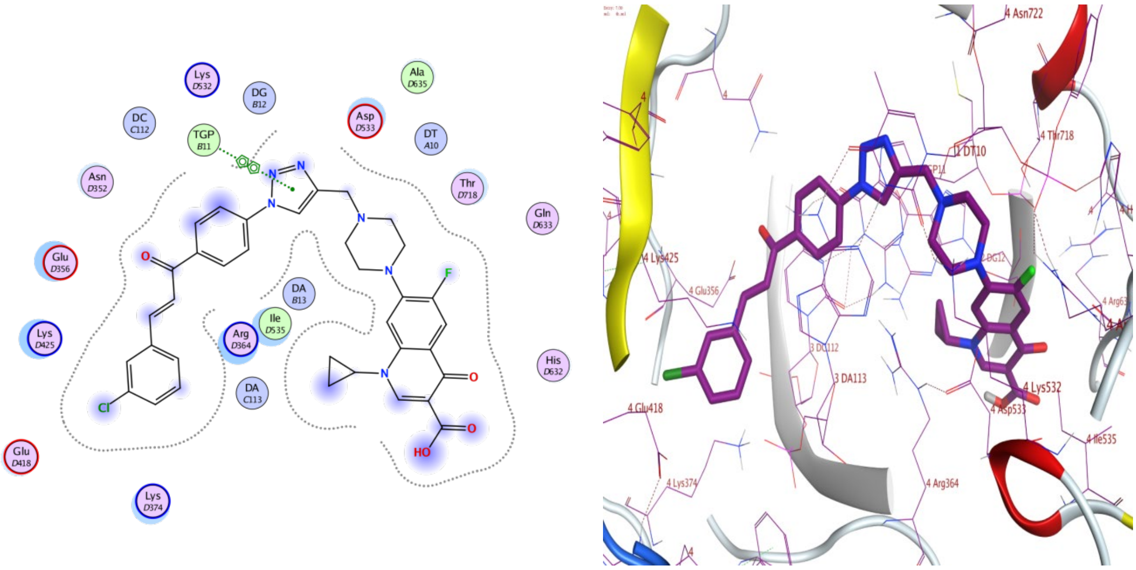

Fig. 1. Binding mode and H-bonds interactions of compound **4b** within **1T8I** active site: (left) 2D interactions (right) 3D structure

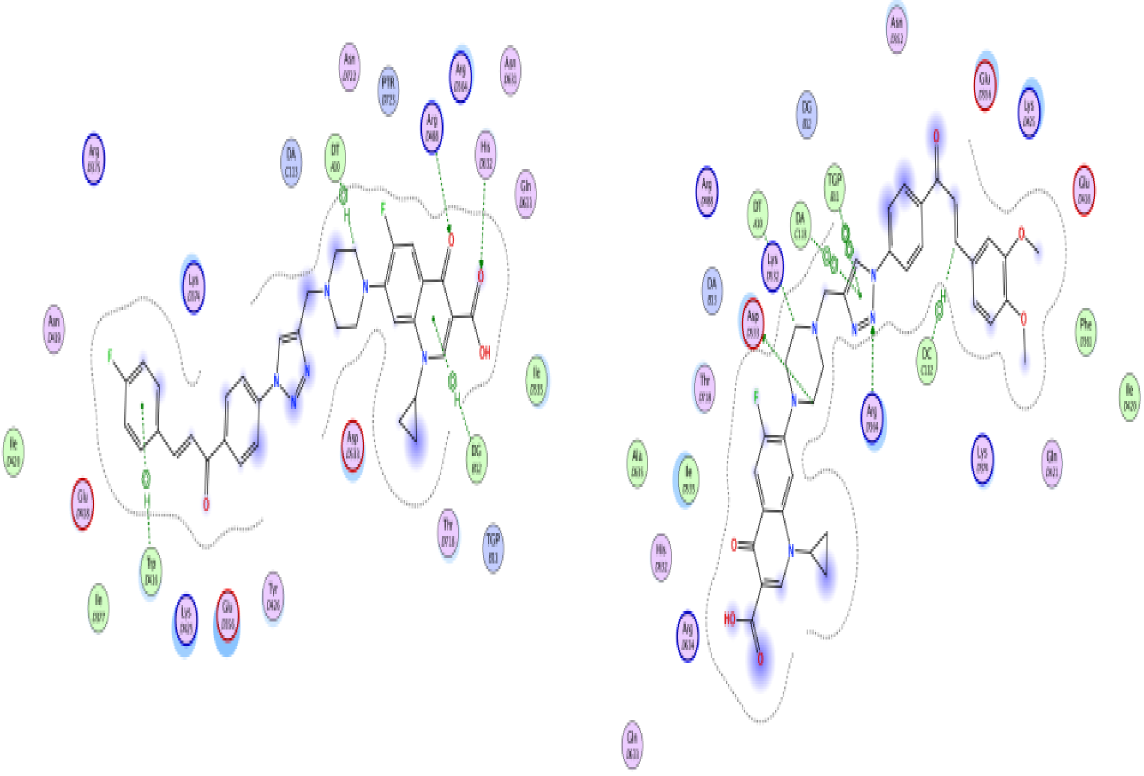

Fig. 2. Binding mode and H-bonds interactions of compound **4e and 4i** within **1T8I** active site: (left) 2D interactions of **4e** (right) 2D interactions of **4i**

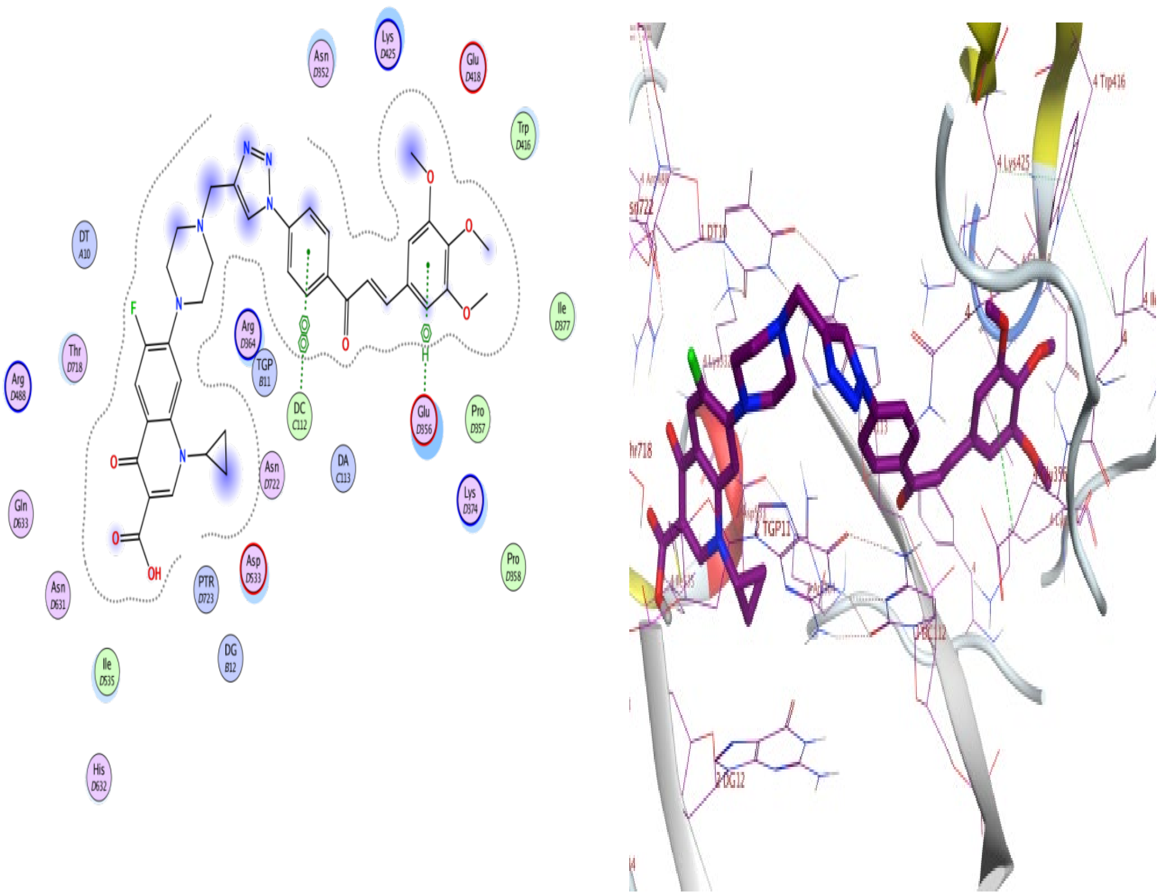

Fig. 3. Binding mode and H-bonds interactions of compound **4j** within **1T8I** active site: (left) 2D interactions (right) 3D structure

Table 3: Molecular modeling data for compounds **4a, 4b, 4e, 4i, 4j**, and camptothecin in Topo II (PDB: ID **6ZY7**)

Compound	Energy score (Kcal/mol)	H-bond forming residues & types	Function group forming H-bond & bond length (Å)	Other interactions residues & types	Function group of interaction & bond length (Å)
**Etoposide**	-10.162	DG 13 (H-donor)<br>DG 5 (H- acceptor)	-OH (2.61 Å)<br>-OH (2.85 Å)	DG 13 (pi-pi)<br>DG 13 (H-pi)	phenyl ring of benzodioxole (3.85 Å)<br>central six member ring (3.61 Å)
**4a**	-9.846	Ala 465 (H- acceptor)<br>Ser 464 (H- acceptor)	-CO of COOH (2.96 Å)<br>-CO of pyridone (2.93Å)		
**4b**	-9.738	Lys 489 (H-donor)	-Cl atom of phenyl ring (3.25Å)		
**4e**	-9.511	Ala 801 (H- donor)<br>Ser 802 (H- acceptor)	-OH of COOH (2.91 Å)<br>-CO of pyridone (3.48Å)	DT 12 (pi-H)<br>DG 13 (pi-H)	Triazole ring (4.50 Å)<br>phenyl ring (4.61 Å)
**4i**	-9.690			DG 13 (pi-H)	Dimethoxy phenyl ring (3.74 Å)
**4j**	-9.906	Ser 800 (H- donor)<br>DC 3 (H- donor)	-N of pyridone (3.11 Å)<br>CH of phenyl ring (2.98 Å)	DC 1 (pi-H)	Trimethoxy Phenyl ring (3.84 Å)<br>Phenyl ring (3.98 Å)

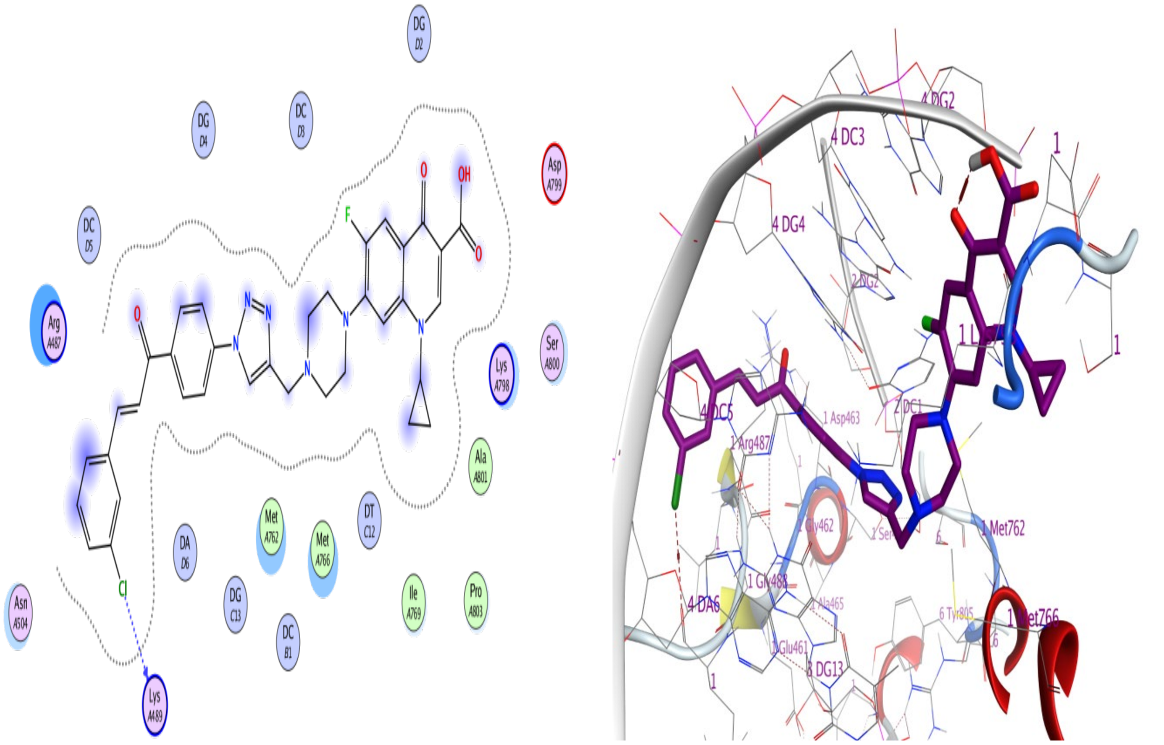

Fig. 4. Binding mode and H-bonds interactions of compound **4b** within **6ZY7** active site: (left) 2D interactions (right) 3D structure

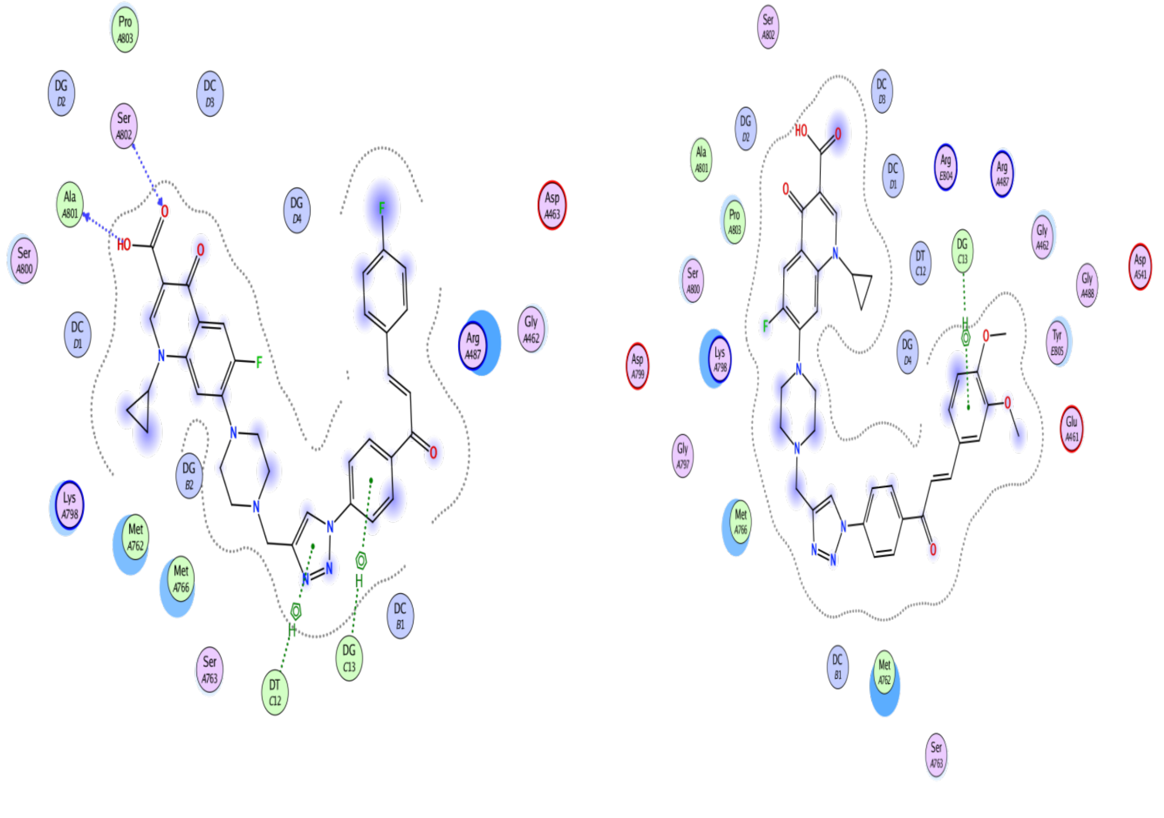

Fig. 5. Binding mode and H-bonds interactions of compound **4e and 4i** within **6ZY7** active site: (left) 2D interactions of **4e** (right) 2D interactions of **4i**

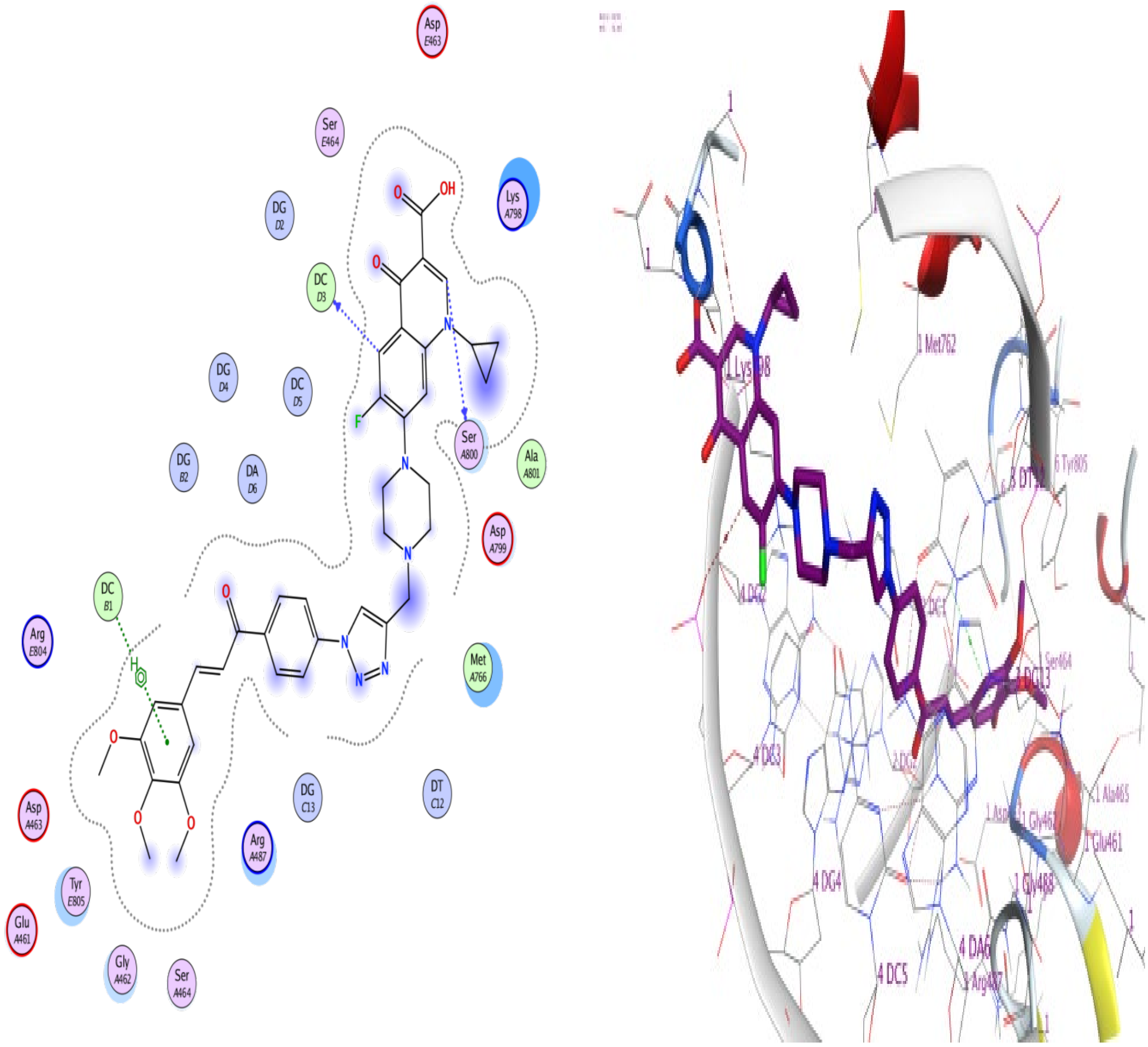

Fig. 6. Binding mode and H-bonds interactions of compound **4j** within **6ZY7** active site: (left) 2D interactions (right) 3D structure
